# Unexpected Nucleophile Masking in Acyl Transfer to Sterically Crowded and Conformationally Restricted Galactosides

**DOI:** 10.1021/acs.joc.3c00878

**Published:** 2023-06-03

**Authors:** Yonatan Sukhran, Israel Alshanski, Ofer Filiba, Megan J. Mackintosh, Igor Schapiro, Mattan Hurevich

**Affiliations:** † The Institute of Chemistry, The Hebrew University of Jerusalem, Jerusalem 91904, Israel; ‡ Fritz Haber Center for Molecular Dynamics, Institute of Chemistry, The Hebrew University of Jerusalem, Jerusalem 91904, Israel

## Abstract

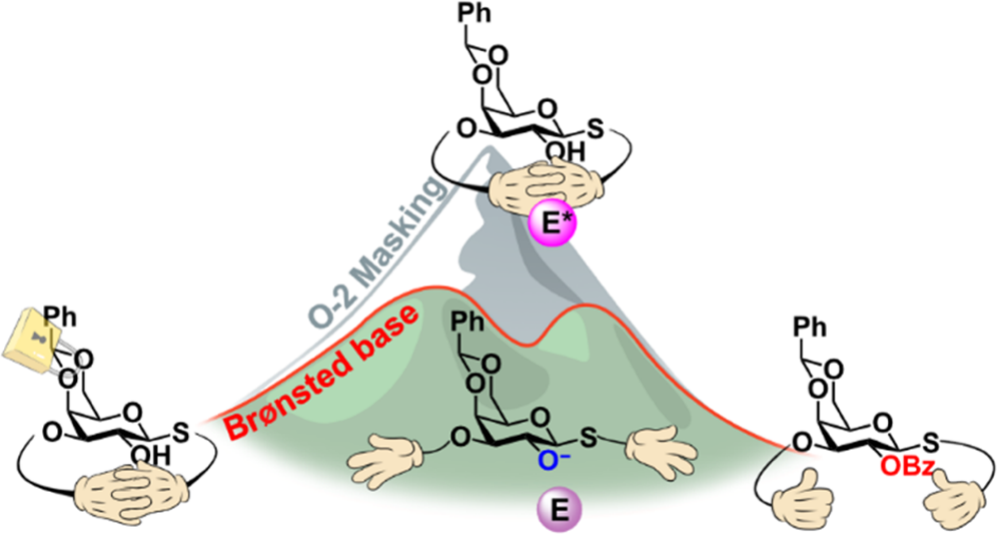

Design and synthesis of orthogonally protected monosaccharide building blocks are crucial for the preparation of well-defined oligosaccharides in a stereo- and regiocontrolled manner. Selective introduction of protecting groups to partially protected monosaccharides is nontrivial due to the often unpredictable electronic, steric, and conformational effects of the substituents. Abolished reactivity toward a commonly used Lewis base-catalyzed acylation of *O*-2 was observed in conformationally restricted 4,6-*O*-benzylidene-3-*O*-Nap galactoside. Investigation of analogous systems, crystallographic characterization, and quantum chemical calculations highlighted the overlooked conformational and steric considerations, the combination of which produces a unique passivity of the 2-*OH* nucleophile. Evaluating the role of electrophile counterion and auxiliary base in the acylation of the sterically crowded and conformationally restricted galactoside system revealed an alternative Brønsted base-driven reaction pathway via nucleophilic activation. Insights gained from this model system were utilized to access the target galactoside intermediate within the envisioned synthetic route. The acylation strategy described herein can be implemented in future syntheses of key monomeric building blocks with unique protecting group hierarchies.

## Introduction

Carbohydrates are bioactive and functionally versatile, making them prime candidates for medical, cosmetic, and sensing applications.^1,2^ Extraction from natural sources yields polysaccharide mixtures with varying stereochemistries, chain lengths, and modification patterns, which display a wide range of bulk properties (e.g., antiviral, anticoagulatory).^3,4^ Their structural diversity and complexity present challenges in obtaining homogeneous oligosaccharides required for the elucidation of biological functions and molecular properties.^5,6^ A bottom-up synthesis of oligosaccharides that relies on the sequential installation of glycosidic bonds with defined stereo- and regiochemistry is crucial for advancing glycochemistry, glycobiology, and related applications.^7^

Synthesis of orthogonally protected glycoside building blocks (BBs) is an essential step toward oligosaccharide assembly.^8^ However, the incredible variety in regiochemistry, stereochemistry, and site-specific modifications requires careful design of the monosaccharide protecting group (PG) hierarchy.^9,10^ Alongside the introduction of new glycosylation strategies, the development of reliable protocols for regioselective installation of PGs is a key part of oligosaccharide synthesis.

**Figure 1:**
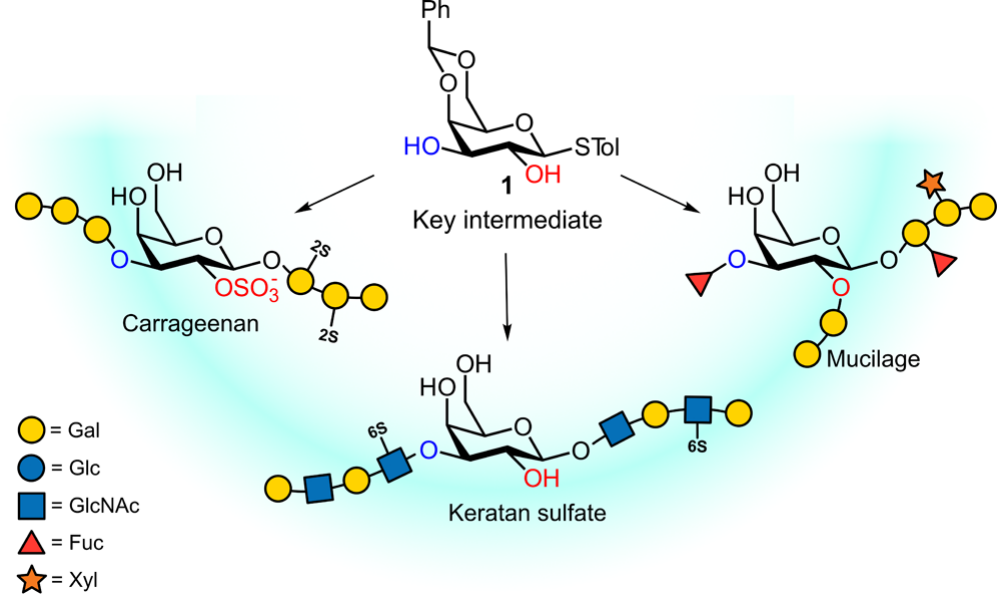
Key intermediate **1** for the synthesis of various oligosaccharides.

Since the emergence of glycoscience, a multitude of strategies for the selective introduction of orthogonal PGs have been established.^11,12^ One common selectivity-providing strategy is the temporary protection of pyranoside *O*-4 and *O*-6 positions with a benzylidene acetal.^13^ The use of conformationally rigid 4,6-*O*-benzylidene glycosides facilitates subsequent manipulation of the free *O*-2 and *O*-3 positions. This makes the 4,6-*O*-benzylidene galactoside **1** a key intermediate in the synthesis of galactose (Gal) BBs of some prominent Gal-containing polysaccharides such as plant mucilage, animal glycosaminoglycan keratan sulfate, and algal carrageenan (Figure 1).^14−16^ We envisioned the selective derivatization of **1** to access BBs for the synthesis of these and other *O*-2- and *O*-3-linked or -modified oligosaccharides.

For the hierarchical PG derivatization of **1**, we wanted to install an *O*-3 benzylic ether 2-naphthylmethyl (*O*-Nap) and an *O*-2 benzoic ester (*O*-Bz). Nap was selected for its mild oxidative deprotection conditions that provide orthogonality to other commonly used PGs.^17^ The acyl-type Bz group was selected for its high stability and stereodirecting properties. Acyl groups at the *O*-2 position provide almost exclusively β stereochemistry of glycosidic bond formation through anchimeric assistance.^11^ Consecutively, the selective introduction of 3-*O*-Nap and 2-*O*-Bz groups to the 2,3-diol **1** and implementation of different glycosylation and deprotection sequences can be used to produce oligosaccharides with varying connectivities and modifications. *En route* to the target BB, we uncovered an unprecedented disfavored reactivity toward a common acylation strategy. We set out to elucidate the origin of this unique behavior.

## Results and Discussion

### Unexpected Failure in Lewis Base-Catalyzed Acylation

**Figure 2:**
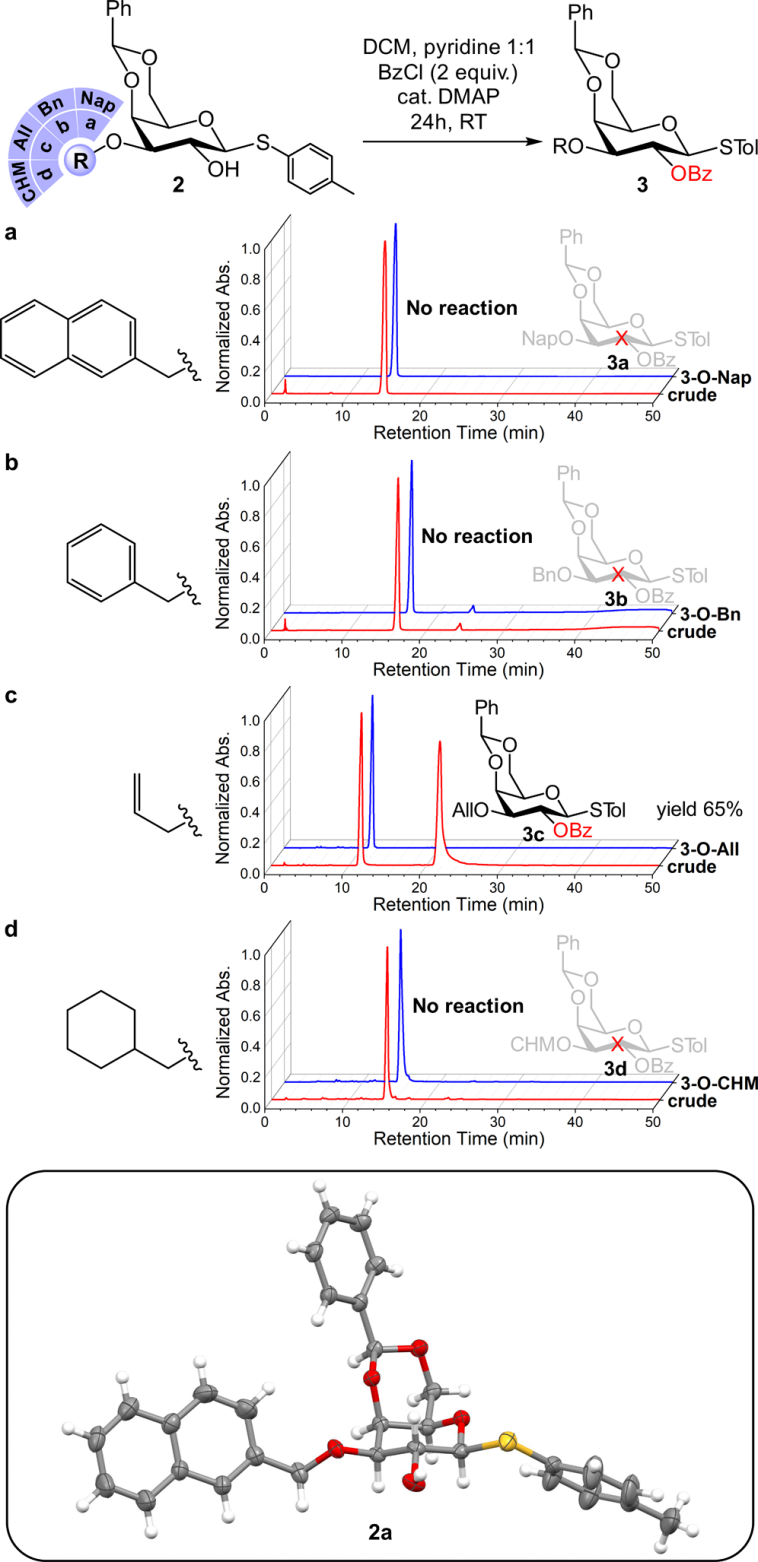
Top: High-performance liquid chromatography (HPLC) trace of crude after DMAP-catalyzed benzoylation of **2a**–**2d**. Bottom: X-ray crystallography-derived thermal ellipsoid plot (*P* = 0.5) of **2a** (CCDC 2173185).

In order to access a β-directing 2-*O*-Bz-protected BB, we chose a stepwise method that relies on selective organotin-mediated *O*-3 etherification, followed by acylation of the free *O*-2 (see the Supporting Information).^18,19^ Strategies for the *O*-3 substitution are more common since *O*-2 substitution strategies in the presence of free *O*-3 generally result in low selectivity at best.^20,21^ Surprisingly, the attempted benzoylation of *O*-2 on the 3-*O*-Nap-substituted derivative **2a** using a common procedure utilizing the pyridine/4-dimethylaminopyridine (DMAP) pair completely failed (Figure 2a). This was unexpected since the DMAP Lewis base–acyl halide combination is widely used for the acylation of partially protected monosaccharide precursors.^22^

We reasoned that given the high reactivity of the DMAP-activated electrophile, masking of the *O*-2 nucleophilic species prevents product formation. The effects of glycan substituents on glycosylation reactivity are well established and extensively studied.^23^ Following similar considerations, substituents in partially protected BBs could modulate the nucleophilicity of unprotected positions. In such a case, the steric bulk and electronic properties of the proximal functional groups could result in unpredictable effects, limiting accessibility to some protected monomers.

### Sterically Driven and *O*-3-Ether-Dependent *O*-2 Masking

To investigate the abolished reactivity of *O*-2 in **2a**, we prepared a library of galactosides **2b–2d** by *O*-3 etherification of **1** (see the Supporting Information). Galactosides **2b** and **2c** were prepared with benzyl (Bn) and allyl (All) *O*-3 ethers, respectively, which represent less sterically demanding, yet electronically analogous substituents to 3-*O*-Nap. While benzoylation of **2b** using the pyridine/DMAP combination resulted in no formation of **3b**, a significant formation of benzoylated **3c** bearing the least bulky 3-*O*-All PG was observed under identical reaction conditions (Figure 2b,c).

It is crucial to consider two geometric aspects of **2a** and **2b**. First, these tolyl thiogalactosides bear bulky aromatic substituents at the positions vicinal to the 2-*OH* nucleophile. Second, the crystallographic structure of **2a** reveals that the 4,6-*O*-benzylidene-protected galactoside assumes a ^4^C_1_ chair conformation (Figure 2, bottom). The geometric constraints of the fused ring system and the bulky substituents hinder chair flip conformational dynamics and likely stabilize an all-adjacent equatorial locked system.^24,25^ The masking of *O*-2 in **2a** and **2b** therefore could stem from steric crowding by adjacent substituents. However, masking through π–π stacking of the tolyl group and the aromatic ether could not be ruled out.

To further elucidate the nature of the masking effect, we prepared the 3-*O*-cyclohexylmethyl (CHM)-protected galactoside **2d**, which is sterically comparable yet electronically dissimilar to **2a** and **2b**. No formation of benzoylated product **3d** was observed under the pyridine/DMAP conditions (Figure 2d). The collective data from benzoylation attempts of **2a**–**2d** indicates that the masking effect in this fused system is steric in nature.

### Quantum Chemical Calculations of the Activation Energies for *O*-2 Benzoylation Reflect *O*-3-Ether Steric Bulk

In order to gain further insight, we performed quantum chemical calculations of the benzoylation of **2a**–**2d** with the activated electrophilic species 1-benzoyl-4-(dimethylamino)-pyridinium (E*) using the B3LYP-D3BJ/6-31G(d,p) level of theory.^26−28^ We theorized that the nucleophilic addition to the carbonyl is the differentiating step between the reactive (**2c**) and nonreactive (**2a**, **2b**, and **2d**) galactosides as the rate-determining step of the acyl transfer cascade.^29^ Importantly, the prerequisite E* formation from the less reactive benzoyl chloride electrophile (**E**) is sugar-independent and identical for the **2a**–**2d** series.

**Figure 3:**
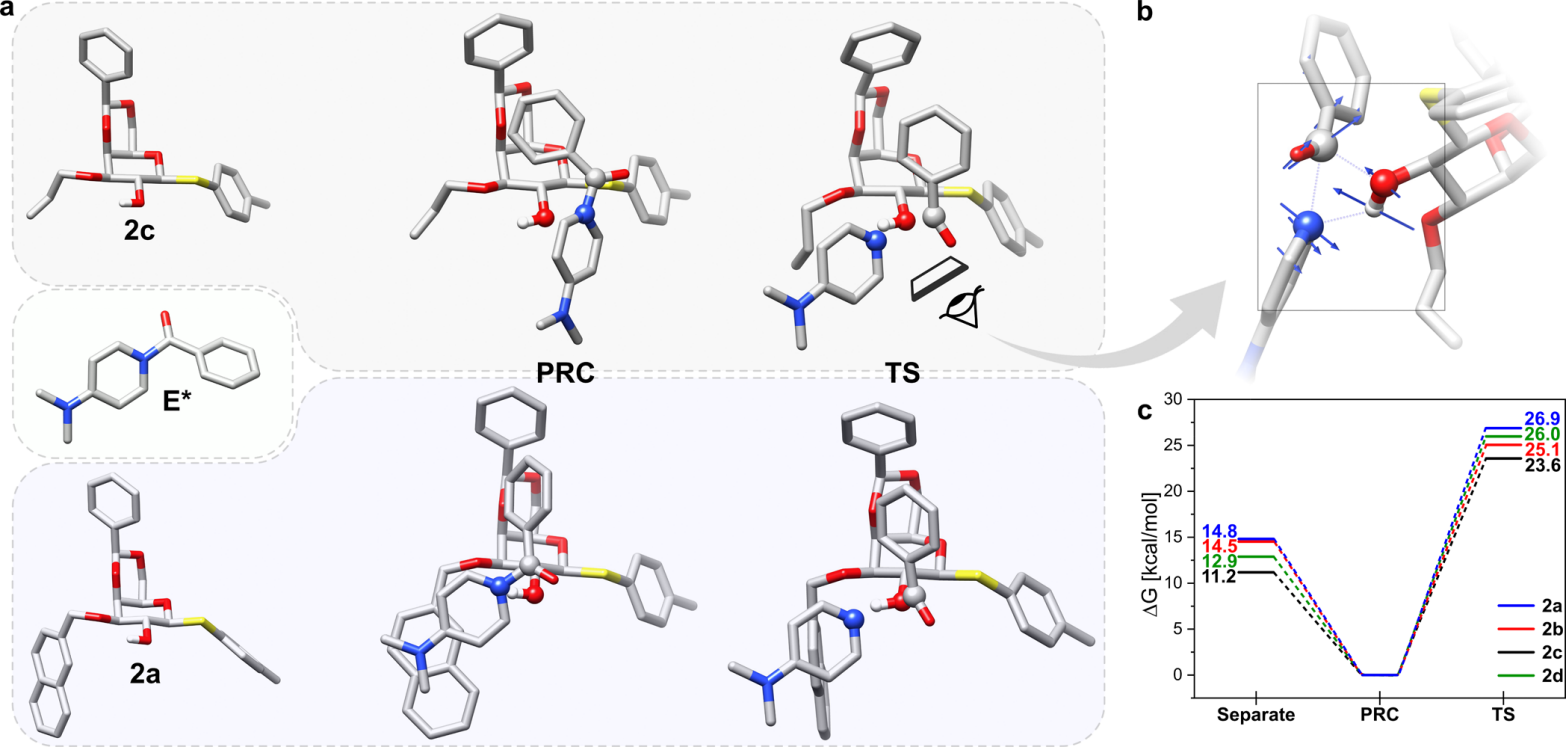
(a) Representative key structures of **2c** (top) and **2a** (bottom) benzoylation (from left to right): (1) separate reactants and E*, (2) prereaction complex (PRC), and (3) transition state (TS). (b) Reaction site of the TS in the acyl substitution of **2c** (vectors corresponding to the motions of atoms represented in blue). (c) Gibbs free-energy profile of the separate reactants, PRC and TS, in the benzoylation of **2a**–**2d** from B3LYP-D3BJ/6-31G(d,p) calculations.

With that in mind, three key structures were obtained: (1) separate galactosides **2a**–**2d** and E*, (2) prereaction complex (PRC), and (3) transition state (TS) of nucleophilic addition to the carbonyl where the TSs were in line with previously reported studies (Figure 3a).^30,31^ Interestingly, a “4-center” TS was obtained that involves acyl substitution and concomitant hydroxyl deprotonation by the pyridyl moiety, which is consistent with the single-stage concerted pathway (Figure 3b).^29,32^ The TS was validated by following the intrinsic reaction coordinate (IRC) to obtain both the separate reactants and the acylation products in a simplified model system (Figure S1).

The Gibbs free energy was calculated for the optimized geometries, with the PRC taken as the reference point for each reaction profile to best reflect the activation energy of the acyl substitution (Figure 3c). The 3-*O*-All-substituted galactoside **2c** that was reactive toward DMAP-catalyzed benzoylation had the lowest activation energy of 23.6 kcal·mol^–1^ for the formation of the TS. A general trend for the activation energy was found where **2c** < **2b** < **2d** < **2a**, which was qualitatively consistent with the steric bulk of the 3-O-ether substituents All < Bn < CHM ∼ Nap. Notably, the PRC was stabilized relative to the infinitely far reactants (“separate”, Figure 3c) for all galactosides. However, the stabilization of **2a** was most significant and may account for the increased activation energy for **2a** relative to **2d** despite the comparable steric bulk of Nap and CHM substituents, respectively. The additional stabilization of the PRC for aryl ether-substituted galactosides can be attributed to π–π interactions of the 4-(dimethylamino)-pyridyl group of E* with 3-*O*-Nap (**2a**) or 3-*O*-Bn (**2b**) as seen from the geometry of the PRC (Figure 3a, bottom, Figure S2).

The differences in the activation energies of the **2a**–**2d** alone do not explain the abolished reactivity of **2a**, **2b**, and **2d**. In solution, other factors and solvent interactions might also play a role. For example, the reaction in solution could proceed through a stepwise mechanism, which involves a solvent-stabilized multicharged tetrahedral intermediate and exhibits even larger sterically driven differences in activation energy than the ones obtained by the gas-phase calculations.^33^ In any case, the calculated TS Gibbs free energies indicate an *O*-3 substituent-dependent steric effect on the acyl transfer, supporting the higher reactivity of the least sterically encumbered **2c** compared to the other galactosides.

### Conformational Restriction of the Fused Ring Galactoside Produces the *O*-2 Masking Effect

**Scheme 1:**
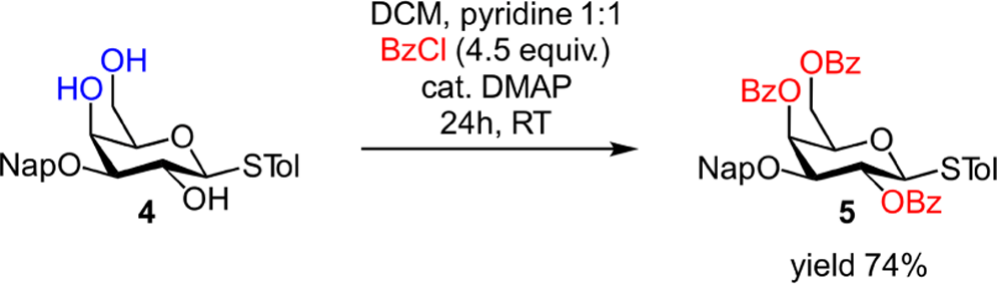
DMAP-Catalyzed Benzoylation of Conformationally Flexible Galactoside **4**

Since benzylidene acetal is commonly used as a temporary PG for a few synthetic steps, the conformational and geometric effects imparted by the fused ring system are often overlooked. The conformationally restricted system could produce unpredicted effects that are related to the conformation-based accessibility of functional groups, exemplified by the steric crowding of adjacent substituents observed in the computationally obtained TS. To evaluate the effect of the conformational restrictions on the masking of *O*-2, the benzylidene acetal was hydrolyzed to yield 3-*O*-Nap-protected 2,4,6-triol **4**, followed by triple benzoylation using the pyridine/DMAP procedure to form the fully protected **5** in good yield (Scheme 1).

The facile benzoylation of **4** but not of conformationally restricted **2a** under analogous conditions indicates that the conformational flexibility of **4** produces a more accessible 2-*OH*, promoting the acylation of *O*-2. It is likely that the benzylidene acetal in **2a** produces additional features that are unique to the galactoside-fused system since *O*-2 masking is not reported for acylation of an analogous conformationally locked thioglucoside.^34^ We suggest that these features can be attributed to the stabilization of different *O*-3-ether orientations in the bicyclic gluco- and galactosides. Additionally, steric crowding of the galactopyranoside face by the benzylidene moiety might be absent in trans-fused bicyclic monosaccharides. Nevertheless, the comparison between **2a** and **4** demonstrates that the passivity of the 2-*OH* nucleophile is linked to the geometric and conformational features of the galactosidefused system. This results in a unique combination of conformational rigidity and steric crowding collectively termed the “*O*-2 masking effect”.

### *O*-2 Masking Results from Synergistic Steric Crowding Surrounding the Reaction Site

Given the geometric aspects of the conformationally constrained fused system, we theorized that the moieties surrounding the reaction site, i.e., the adjacent substituents and the electrophilic species, would produce the most considerable steric effects. To further characterize the individual contributions to the *O*-2 masking effect, we evaluated the impact of these three components on the acylation yields by (1) replacing the *O*-3-Nap with an *O*-3-All substituent, (2) introducing a smaller acetyl chloride/anhydride electrophile, and (3) comparing the reactivity of a thioethyl (SEt) and thiotolyl (STol) galactosides (Table 1).

**Table 1:**
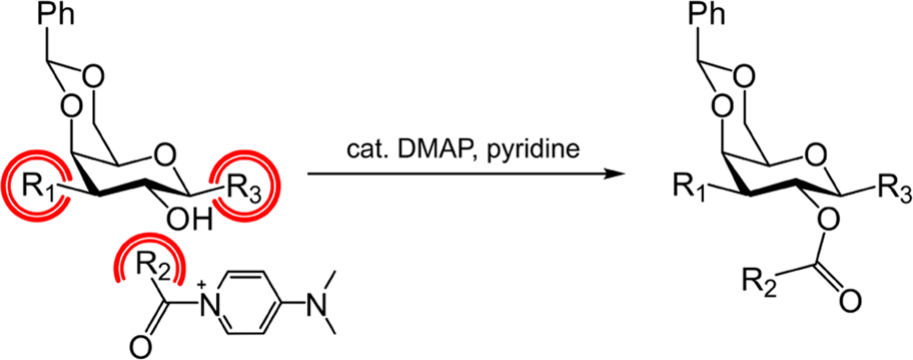
Acylation of *O*-2 in Fused Ring Thiogalactoside Analogues

entry	R_1_	R_2_	R_3_	yield (%)
**3c**^a^	OAll	Ph	STol	65
**6**^b^	ONap	Me	STol	81/100^c^
**7**(35)	ONap	Ph	SEt	96

a 2 equiv of BzCl, cat. DMAP, DCM/pyridine (1:1), 24 h, RT.

b 2 equiv of AcCl/Ac_2_O, cat. DMAP, DCM/pyridine (1:1), overnight, RT.

c Yields for AcCl and Ac_2_O.

Alleviating any one steric factor by the substitution of either vicinal position (R_1_ and R_3_) or electrophile (R_2_) with a less bulky derivative resulted in a dramatic increase in the acylation of *O*-2. This almost binary substituent-dependent reactivity toward pyridine/DMAP-catalyzed acylation in the fused system implies a synergistic crowding effect that results from the collective steric interactions around the reaction site. We suggest that replacing either R_1_, R_2_, or R_3_ with an aliphatic group could mitigate some aromatic dispersion interactions that hinder the approach of E* to the *O*-2 nucleophile in **2a**. Off-note, using either acetic anhydride or acetyl chloride as an electrophile resulted in full conversion. However, the latter proved more challenging to purify, likely due to a known side reaction of acetyl chloride with pyridine (Figure S3).^36^

### Brønsted Base Alleviates the *O*-2 Masking Effect

**Figure 4:**
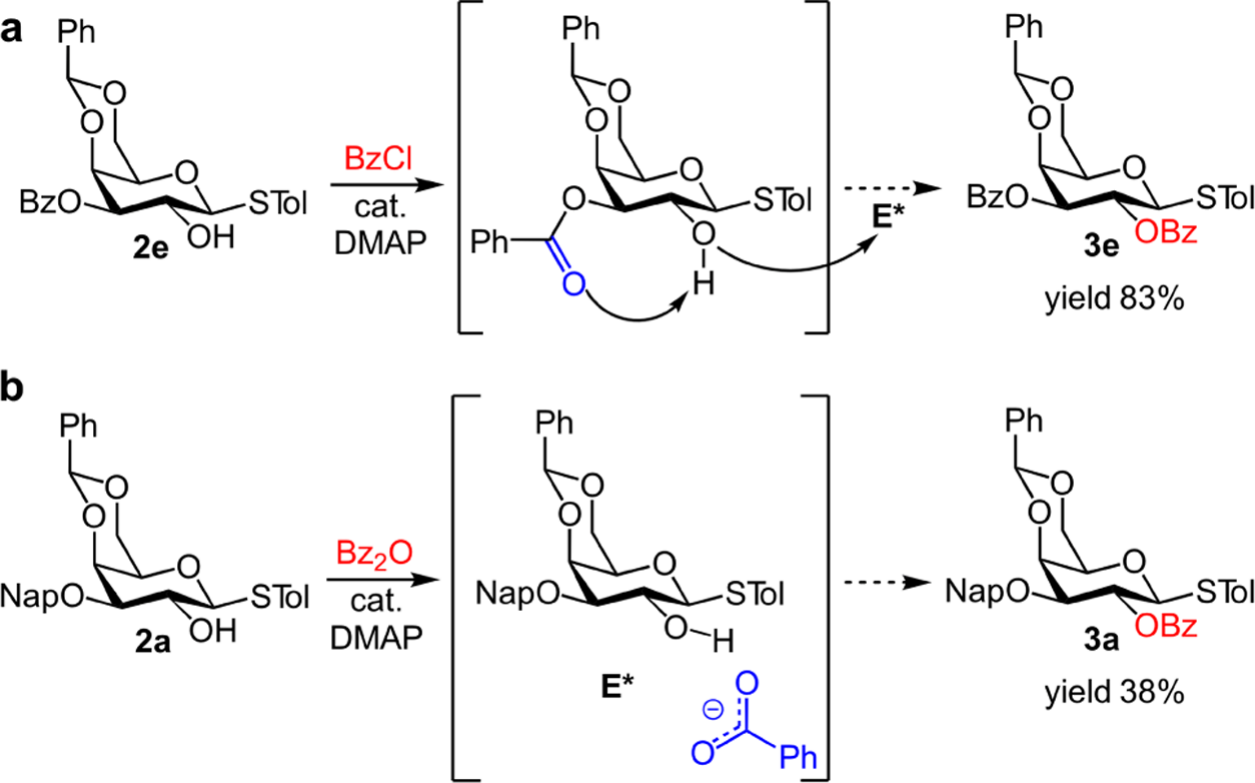
(a) Internal base-catalyzed benzoylation of **2e** in Lewis base-mediated system. (b) Benzoate-assisted benzoylation of **2a**. 2 equiv of BzCl/Bz_2_O, cat. DMAP, DCM/pyridine (1:1), 24 h, RT.

Interestingly, despite the steric considerations described above, the bulky 3-*O*-Bz ester-protected galactoside **2e** readily reacted to form **3e** under pyridine/DMAP-catalyzed benzoylation conditions in excellent yield (Figure 4a). We hypothesized that the reaction was propelled by internal base catalysis of the *O*-3-ester, which is absent in the sterically analogous 3-*O*-Bn **2b** or any other 3-*O*-ether-protected derivatives (**2a**, **2c**, and **2d**). The possibility of an acyl migration-mediated mechanism was ruled out since subjecting **2e** to similar conditions in the absence of an electrophile produced no reaction, in line with previous reports (Figure S4).^22^

Moreover, the reaction of **2a** with benzoic anhydride instead of benzoyl chloride under analogous conditions resulted in significant, albeit incomplete, formation of **3a** (Figure 4b). The decisive role of the carboxylate counterion in DMAP-catalyzed acylation has been reported.^37,38^ It is plausible that the benzoate counterion facilitates the acylation reaction by 2-*OH* proton coordination.^31^ The reaction is unlikely to follow an entirely different pathway that does not involve the formation of E* since a higher amount of DMAP is reported to improve the yield.^39^ We suggest that the benzoate counterion serves as a Brønsted base to promote acylation via nucleophilic activation.

Since our results outlined the importance of a base for *O*-2 benzoylation in the fused Gal system, the use of an external base was envisioned to enable the conversion of **2a** to **3a**. Unlike Lewis base-mediated activation of electrophilic species, Brønsted base has been previously used for the nucleophilic activation of hydroxyl substrates or in combination with a Lewis base catalyst.^40^ The role of triethylamine (TEA) as an auxiliary base for the regeneration of the Lewis base catalyst has been previously indicated in a simple system.^31^ However, we theorized that in our unique fused system, an external base could favor a pathway involving the formation of a strongly nucleophilic alkoxide species, potentially overcoming the *O*-2 masking effect encountered under pyridine/DMAP benzoylation conditions.

**Figure 5:**
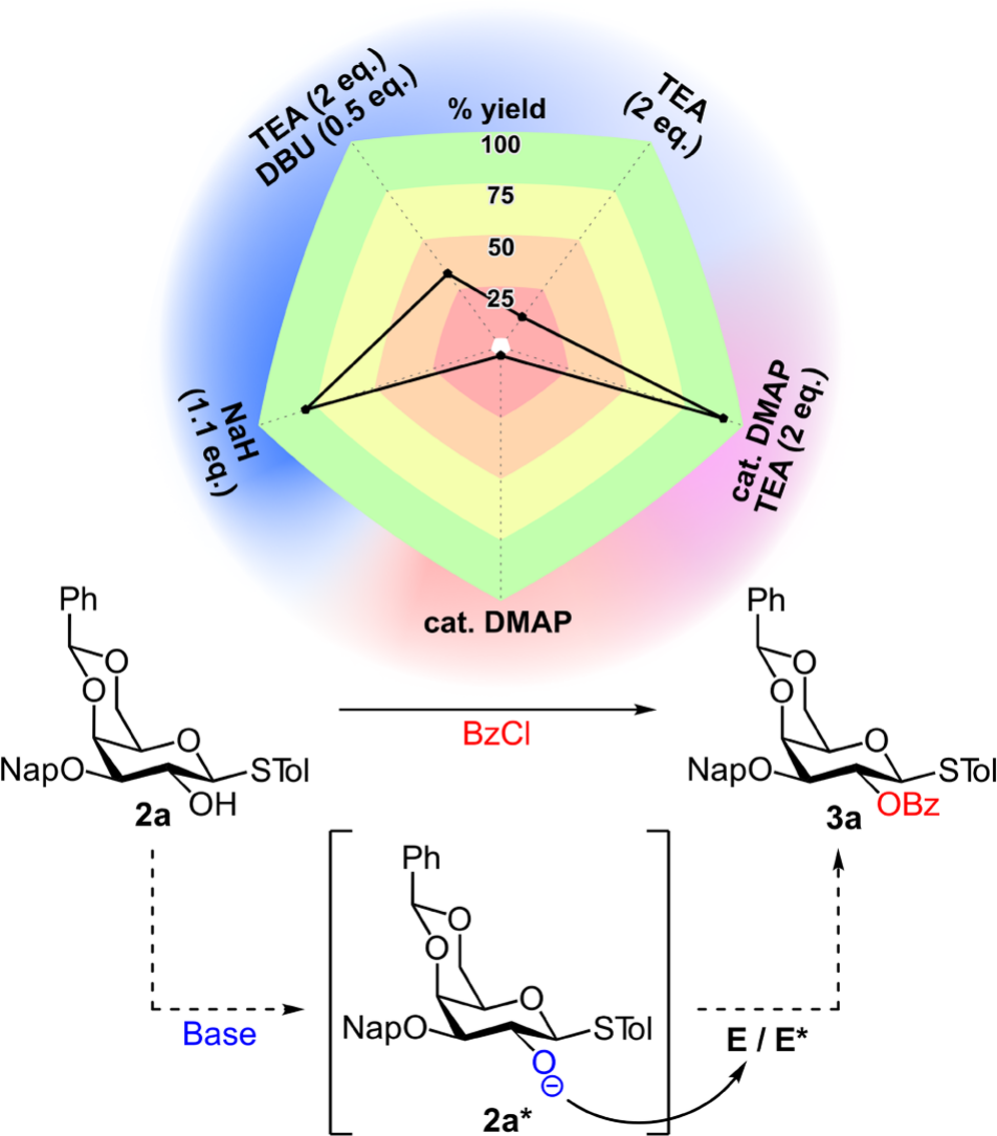
Yields for benzoylation of **2a** with various Lewis/Brønsted bases (counterclockwise): cat. DMAP, cat. DMAP/TEA, TEA, TEA/DBU, NaH (see the Supporting Information). Acylation of **2a** via charged intermediate 2a* in the Brønsted base-driven reaction pathway.

In our initial attempt, galactoside **2a** was first deprotonated with sodium hydride and subsequently reacted with **E**. The benzoylated product **3a** was afforded in good yield by utilizing this procedure (78%, Figure 5). Sodium hydride is an exceptionally strong base (H_2_ p*K*_a_ = 35), which irreversibly deprotonates the 2-*OH* as indicated by hydrogen gas evolution, producing an extremely reactive activated nucleophilic species. Therefore, it is safe to assume that the formation of the anionic intermediate 2a* followed by acyl substitution by an alkoxide are key steps in the reaction pathway under basic conditions.

Using a moderately basic mixture of 1,8-diazabicyclo(5.4.0)undec-7-ene (DBU) and TEA, or mildly basic TEA resulted in significantly lower, yet non-negligible formation of **3a** (33 and 12%, respectively, Figure 5). Since nonconjugated tertiary amines (e.g., DBU, TEA) are poor electrophilic activators, the reaction must proceed through a similar pathway involving proton abstraction to form 2a*.^41^ Conversely, a weakly basic pyridine/DMAP mixture would not be able to promote benzoylation of the masked *O*-2 via a pathway involving proton abstraction for the formation of 2a*. A general trend of increase in the acylation yields for increasingly basic conditions implies a predominant Brønsted base-driven reaction pathway involving the formation of an anionic intermediate 2a* in the fused system.

Since an alkoxide is a strongly nucleophilic species, the bottleneck for the proposed Brønsted base-driven *O*-2 acylation pathway, in the absence of an activated E*, should be the accessibility of the electrophile to the reaction site. Unmasking of *O*-2 by proton abstraction can be rationalized by considering several effects. The formation of the negatively charged species could promote structural reorganization and consecutively alleviate steric crowding around the *O*-2 nucleophile, which could facilitate the approach along the Bürgi–Dunitz angle and enable the benzoylation.^42,43^ The anionic intermediate 2a* is likely destabilized by the presence of multiple electron-donating substituents (4,6-*O*-benzylidene, 3-*O*-Nap). The latter also provides an explanation for the considerable formation of **3a** using near-equimolar amounts of a hydride base as it proceeds via irreversible proton abstraction, while even excess of a tertiary amine base, which acts in equilibrium, leads only to the minor formation of **3a**. In any case, we suggest that the formation of the anionic intermediate effectively results in the unmasking of an otherwise inaccessible *O*-2 position in the fused system, thereby enabling the subsequent benzoylation.

Importantly, neither catalytic amounts of DMAP nor mildly basic conditions with TEA were individually successful in facilitating the benzoylation of **2a**. However, a combination of DMAP Lewis base and TEA Brønsted base resulted in the formation of **3a** in excellent yield (92%, Figure 5). It is possible that a low fraction of the deprotonated unmasked *O*-2 nucleophile, formed in equilibrium under mildly basic conditions, could rapidly react with highly reactive intermediate E* and push the reaction to completion. The reaction could be further propelled by a dual function of TEA that serves as an auxiliary base for catalyst regeneration in addition to its role as a nucleophilic activator. We suggest that the acylation is greatly facilitated by a combination of electrophilic and nucleophilic activation agents, resulting in a reaction pathway involving the concurrent formation of the strongly electrophilic E* and the strongly nucleophilic 2a* species.

The findings exemplify that the reactivity of hydroxyls in partially protected BBs is hard to predict, being heavily influenced by adjacent substituents and conformational constraints. However, a thorough investigation of the *O*-2 masking effect resulted in the identification of benzoylation conditions for the facile synthesis of differentially *O*-2- and *O*-3-protected galactosides via a Brønsted base-driven pathway. This model system with a uniquely masked *O*-2 nucleophile brought forth insight into a seemingly simple acylation reaction and demonstrated an alternative competing reaction pathway that should be taken into consideration in the context of sterically crowded poorly nucleophilic species.

## Conclusions

Development of reliable and efficient protocols for the preparation of protected BBs is the pillar of oligosaccharide synthesis. *En route* to a fully protected galactoside BB, we uncovered an unexpected *O*-2 masking effect in DMAP-catalyzed acylation via electrophilic activation. By combining crystallographic data, quantum chemical calculations, and design of analogous systems, we established that conformational rigidity and steric bulk of vicinal substituents and electrophile are major components in producing the uncharacteristic passivity of the *O*-2 nucleophile toward this seemingly straightforward transformation.

We found that the unique features of the partially protected, conformationally restricted, fused galactoside allowed us to separately explore various aspects of the acylation reaction, which can be easily overlooked in less intricate systems. We demonstrated the role of the benzoate counterion and Brønsted base in promoting benzoylation via nucleophilic activation of the galactoside hydroxyl rather than the commonly used carbonyl electrophile activation. A conformational shift and destabilization of the anionic intermediate are plausible factors in the Brønsted base-driven pathway that effectively results in unmasking of the *O*-2 position.

We conducted a comprehensive investigation of the *O*-2 masking effect and gained insight into the major factors influencing the acylation reaction in the fused system. The study highlights that the preferred acyl installation method should be rationalized mechanistically in the context of the substrate. The understanding of the factors involved in the acylation of the fused galactoside system was utilized to find an optimized strategy for obtaining the target monosaccharide within the envisioned stepwise regioselective introduction of PGs. We intend to implement this scalable method to produce and further derivatize *O*-2 and *O*-3 differentially protected galactosides to access monosaccharides with a unique PG hierarchy that are pivotal to the preparation of complex glycans.

## Data Availability

The data underlying this study are available in the published article and its Supporting information.

## Supporting Information Available

General methods; experimental protocols for the preparation and characterization of all compounds including ^1^H NMR, ^13^C NMR, ^1^H–^1^H COSY, ^1^H–^13^C HSQC, and HRMS; computational methods and additional structures including model system and IRC calculations and structures of **2b** and **2d** (PRC and TS) (PDF)
Coordinates of molecular geometries from DFT calculations. *xyz* coordinates of the optimized structures for separate reactants, PRC and TS of **2a–2d** and **E*** (XYZ)

## Supplementary Material

jo3c00878_si_001.pdf

jo3c00878_si_002.xyz
