# Supplementary material for: Unexpected Nucleophile Masking in Acyl Transfer to Sterically Crowded and Conformationally Restricted Galactosides
Source: J Org Chem. 2023 Jun 3;88(13):9313–20. doi: 10.1021/acs.joc.3c00878 (PMC10337027; doi:10.1021/acs.joc.3c00878)
Supplement: Supplementary file 1 — jo3c00878_si_001.pdf [file jo3c00878_si_001.pdf]

Supplementary information

Unexpected Nucleophile Masking in Acyl Transfer to Sterically Crowded and Conformationally Restricted Galactosides

Yonatan Sukhran‡, Israel Alshanski‡, Ofer Filiba†, Megan J. Mackintosh†, Igor Schapiro†*, Mattan Hurevich‡*.

‡The Institute of Chemistry, The Hebrew University of Jerusalem, Jerusalem 91904, Israel.

†Fritz Haber Center for Molecular Dynamics, Institute of Chemistry, The Hebrew University of Jerusalem, Jerusalem 91904, Israel.

E-mail: Mattan.Hurevich@mail.huji.ac.il

Table of Contents

Experimental Section S-4

General methods S-4

Building block synthesis S-4

***p*-Tolyl 4,6-O-benzylidene-1-thio-β-D-galactopyranoside (1)** S-4

***p*-Tolyl 4,6-O-benzylidene-3-O-(2-naphthalenylmethyl)-1-thio-β-D-galactopyranoside (2a)** S-5

***p*-Tolyl 3-O-benzyl-4,6-O-benzylidene-1-thio-β-D-galactopyranoside (2b)** S-6

***p*-Tolyl 3-O-allyl-4,6-O-benzylidene-1-thio-β-D-galactopyranoside (2c)** S-6

***p*-Tolyl 4,6-O-benzylidene-3-O-(cyclohexylmethyl)-1-thio-β-D-galactopyranoside (2d)** S-7

***p*-Tolyl 3-O-benzoyl-4,6-O-benzylidene-1-thio-β-D-galactopyranoside (2e)** S-7

***p*-Tolyl 2-O-benzoyl-4,6-O-benzylidene-3-O-(2-naphthalenylmethyl)-1-thio-β-D-galactopyranoside (3a)** . S-8

General procedure for DMAP catalyzed benzoylation (3) S-9

***p*-Tolyl 3-O-allyl-2-O-benzoyl-4,6-O-benzylidene-1-thio-β-D-galactopyranoside (3c)** S-10

***p*-Tolyl 2,3-O-dibenzoyl-4,6-O-benzylidene-1-thio-β-D-galactopyranoside (3e)** S-10

***p*-Tolyl 3-O-(2-naphthalenylmethyl)-1-thio-β-D-galactopyranoside (4)** S-11

***p*-Tolyl 2,4,6-O-tribenzoyl-3-O-(2-naphthalenylmethyl)-1-thio-β-D-galactopyranoside (5)** S-11

***p*-Tolyl 2-O-acetyl-4,6-O-benzylidene-3-O-(2-naphthalenylmethyl)-1-thio-β-D-galactopyranoside (6)** S-12

Computational methods S-13

S3. Formation of side products in acetylation of **2a** with AcCl to form Compound **6** S-15

S4. Comparative experiment for Compound **2e** S-15

Spectroscopic data S-16

Compound **1** S-16

^{1}H-NMR S-16

^{1}H-^{1}H COSY S-17

^{1}H-^{13}C HSQC S-18
^{13}C{^{1}H} NMR S-19
Compound **2a** S-20
^{1}H-NMR S-20
^{1}H-^{1}H COSY S-21
^{1}H-^{13}C HSQC S-22
^{13}C{^{1}H} NMR S-23
Compound **2b** S-24
^{1}H-NMR S-24
^{1}H-^{1}H COSY S-25
^{1}H-^{13}C HSQC S-26
^{13}C{^{1}H} NMR S-27
Compound **2c** S-28
^{1}H-NMR S-28
^{1}H-^{1}H COSY S-29
^{1}H-^{13}C HSQC S-30
^{13}C{^{1}H} NMR S-31
Compound **2d** S-32
^{1}H-NMR S-32
^{1}H-^{1}H COSY S-33
^{1}H-^{13}C HSQC S-34
^{13}C{^{1}H} NMR S-35
Compound **2e** S-36
^{1}H-NMR S-36
^{1}H-^{1}H COSY S-37
^{1}H-^{13}C HSQC S-38
^{13}C{^{1}H} NMR S-39
Compound **3a** S-40
^{1}H-NMR S-40
^{1}H-^{1}H COSY S-41
^{1}H-^{13}C HSQC S-42
^{1}H-^{13}C HMBC S-43

$^{13}C\{^{1}H\}$ NMR S-44
Compound **3c** S-45
^{1}H-NMR S-45
^{1}H-^{1}H COSY S-46
^{1}H-^{13}C HSQC S-47
$^{13}C\{^{1}H\}$ NMR S-48
Compound **3e** S-49
^{1}H-NMR S-49
^{1}H-^{1}H COSY S-50
^{1}H-^{13}C HSQC S-51
$^{13}C\{^{1}H\}$ NMR S-52
Compound **4** S-53
^{1}H-NMR S-53
^{1}H-^{1}H COSY S-54
^{1}H-^{13}C HSQC S-55
$^{13}C\{^{1}H\}$ NMR S-56
Compound **5** S-57
^{1}H-NMR S-57
^{1}H-^{1}H COSY S-58
^{1}H-^{13}C HSQC S-59
$^{13}C\{^{1}H\}$ NMR S-60
Compound **6** S-61
^{1}H-NMR S-61
^{1}H-^{1}H COSY S-62
^{1}H-^{13}C HSQC S-63
$^{13}C\{^{1}H\}$ NMR S-64
References S-65

Experimental Section

General methods

Reagent-grade commercial chemicals were used without purification unless mentioned. All solvents were dried if necessary according to the procedure using molecular sieves. Reactions were carried under Ar or N_2 atmosphere. Thin-layer chromatography (TLC) was carried out on aluminum sheets coated with silica gel (Merck). TLC plates were developed by treatment with p-anisaldehyde stain. Flash chromatography was performed in silica gel column with ethyl acetate (EtOAc) hexane gradient, elution is reported for % EtOAc by volume. Prep-HPLC was performed in Phenomenex Luna 5µm C18 column with acetonitrile (ACN) and triply-distilled water (TDW) with 0.1% (*v*/*v*) trifluoroacetic acid (TFA) gradient, elution is reported for % ACN by volume. Analytical HPLC was performed in XTerra 5µm RP-8, 4.6x150mm column with ACN:TDW with 0.1% (*v*/*v*) TFA gradient at 1ml/min flow. NMR spectra were recorded on Bruker Avance-IV-500 (500MHz) or Bruker Avance Nanobay (400MHz) spectrometer. The structural assignments of ^{1}H spectra were made with additional information from gCOSY, gHSQC and gHMBC. Chemical shift reported in ppm, referenced to solvent residual peak (^{1}H-NMR δ 7.26/^{13}C-NMR δ 77.0 for $CDCl_3$, ^{1}H-NMR δ 2.05/^{13}C-NMR δ 29.82 for d_6-acetone, ^{1}H-NMR δ 5.32/^{13}C-NMR δ 53.8 for d_2-dichloromethane).

Building block synthesis

p-Tolyl 4,6-O-benzylidene-1-thio-β-D-galactopyranoside (1)

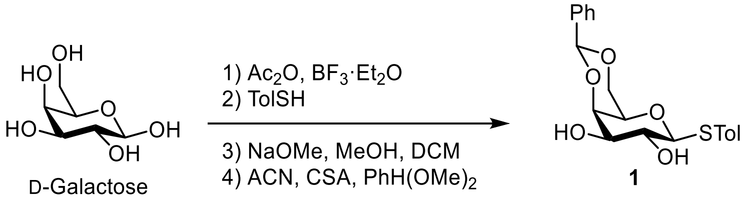

A suspension of D-galactose (10.1 g, 56.1 mmol) in acetic anhydride (Ac_2O) (28 ml, 296 mmol) was placed in an ice bath under Ar atmosphere with continuous stirring. BF_3·Et_2O (12 ml, 97 mmol) was added dropwise over 15 min. An exothermic reaction occurred, the reaction mixture was kept under 30°C with a water bath. After 2h the solution turned clear and per-acetylated galactose formation was complete (as indicated by TLC, 40% EtOAc in hexane Rf=0.44). *p*-Thiocresol (TolSH) (7.6 g, 61 mmol) was added in one portion. The reaction was complete after 24h at 30°C (as indicated by TLC, 40% EtOAc in hexane R_f=0.68). The reaction mixture was diluted with dichloromethane (DCM) (50 ml), then carefully quenched with sat. $NaHCO_3$. The organic layer was separated, washed with sat. $NaHCO_3$ and brine, dried over Na_2SO_4 and gravitationally filtered. The organic layer was concentrated *in vacuo* to yield crude *p*-tolyl-2,3,4,6-tetracetyl-1-thio-β-D-galactopyranoside. The crude product was dissolved in dry DCM and dry MeOH (300 ml, 1:1) and placed in an ice bath. NaOMe (1.59 g, 29.4 mmol) was added and the reaction mixture allowed to reach RT. The reaction was stirred for 3h until completion (as indicated by TLC, 10% MeOH in DCM, R_f=0.48). Ion exchange beads Amberlight® XAD16N were added until neutral pH was reached and reaction mixture turned clear. The reaction mixture was filtered and concentrated *in vacuo* to yield a yellow oil. The residue was triturated with n-hexane repeatedly to remove residual TolSH until the decanted solution was colorless. The oily residue was dissolved in minimal amount of n-butanol and vacuum filtered to remove precipitated D-galactose, then concentrated *in vacuo*. The residue was triturated with n-hexane to yield *p*-tolyl-1-thio-β-D-galactopyranoside as off-white solid in sufficient purity (15.03 g, 3-step yield 94%). *p*-Tolyl-1-thio-β-D-galactopyranoside (11 g, 38 mmol) was suspended in dry ACN (150 ml) and cooled in an ice bath. Benzaldehyde dimethyl acetal (PhH(OMe)$_2$) (6.7 ml, 44 mmol) and camphorsulfonic acid (CSA) (1.7 g, 7.3 mmol) were added. Acidity was confirmed to be pH=1. Reaction was complete after 3h at RT (as indicated by TLC, 70% EtOAc in hexane, R_f=0.35). The reaction was quenched by addition of triethylamine (TEA) until neutral (~1ml) and the solvent was removed *in vacuo*. The crude product was re-dissolved in DCM (150 ml).

The organic layer was washed with $NaHCO_3$ and brine, dried over Na_2SO_4 and gravitationally filtered. The crude solid was triturated with 10% EtOAc in hexane. Sufficiently pure **1** (10.51 g, yield 74%) was obtained and used later without further purification. HRMS (ESI-Q-Orbitrap) m/z: $[M+H]^+$ Calcd for $C_{20}H_{23}O_5S$ 375.1261; found 375.1262. ^{1}H NMR (400MHz, CD_2Cl_2): δ 7.54 (m, 2H, *o*-Tol), δ 7.42-7.37 (m, 5H, *o,m,p*-PhCHOO), δ 7.13 (m, 2H, *m*-Tol), δ 5.52 (s, 1H, PhC***H***OO), δ 4.48 (d, $J_{1,2}$=9.1Hz, 1H, H-1(Gal)), δ 4.31, 4.03 (ABdq, $\Delta\delta_{AB}$=0.28, J_{AB}=12.5Hz, $J_{5,6}$=1.7Hz, 2H, H-6(Gal)), δ 4.21 (dd, $J_{3,4}$=3.4Hz, $J_{4,5}$=1.2Hz, 1H, H-4(Gal)), δ 3.69-3.64 (m, 1H, H-3(Gal)), δ 3.60 (dd, $J_{1,2}$=$J_{2,3}$=9.2Hz, 1H, H-2(Gal)), δ 3.54 (m, 1H, H-5(Gal)), δ 2.52-2.40 (w, 2H, 2,3-OH (Gal)), δ 2.36 (s, 3H, S-Ph-C***H***$_3$), traces of residual EtOAc. ^{13}C{^{1}H} NMR (101MHz, CD_2Cl_2): δ 138.9 (*p*-Tol), δ 138.3 (*i*-Tol), δ 134.1 (*o*-Tol), δ 130.1 (*m*-Tol), δ 129.6 (PhCHOO), δ 128.5 (PhCHOO), δ 127.9 (PhCHOO), δ 126.9 (PhCHOO), δ 101.6 (Ph**C**HOO), δ 88.0 (C-1(Gal)), δ 75.8 (C-4(Gal)), δ 74.2 (C-3(Gal)), δ 70.5 (C-5(Gal)), δ 69.7 (C-6(Gal)), δ 69.4 (C-2(Gal)), δ 21.3 (S-Ph-C***H***$_3$). The ^{1}H-NMR and ^{13}C-NMR spectra are in agreement with the literature.[1]

***p*-Tolyl 4,6-O-benzylidene-3-O-(2-naphthalenylmethyl)-1-thio-β-D-galactopyranoside (2a)**

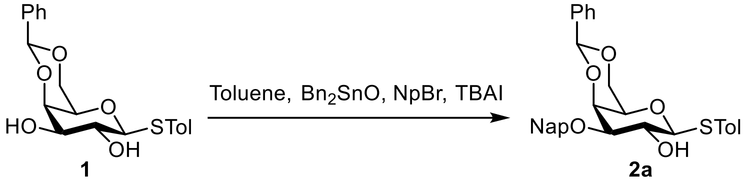

Compound **1** (584 mg, 1.56 mmol) and dibutyltin oxide (408 mg, 1.64 mmol) were suspended in dry toluene (25 ml). The reaction mixture was heated in an oil bath and refluxed for 3h in Dean-Stark apparatus equipped with a drying tube until no further water collection was observed. Reaction mixture was allowed to cool to 70°C and 2-naphthalenylmethyl bromide (NapBr) (0.52 g, 2.4 mmol) and tetrabutylammonium iodide (TBAI) (0.89 g, 2.4 mmol) were added. The reaction was left for overnight in an oil bath at 70°C until completion (as indicated by TLC, 70% EtOAc in hexane, R_f=0.68). The reaction mixture was concentrated *in vacuo* and purified by flash chromatography to obtain **2a** (420 mg, 52% yield, elution at 40% EtOAc in hexane). HRMS (ESI-TOF) m/z: $[M+H]^+$ Calcd for $C_{31}H_{31}O_5S$ 515.1892; found 515.1893. ^{1}H NMR (500MHz, CD_2Cl_2): δ 7.84-7.78 (m, 4H, Nap), δ 7.54 (d, $J_{o,m}$=8.2Hz, 2H, *o*-Tol), δ 7.50-7.46 (m, 3H, Nap), δ 7.42-7.38 (m, 5H, *o,m,p*-PhCHOO), δ 7.11 (d, $J_{o,m}$=7.9Hz, 2H, *m*-Tol), δ 5.45 (s, 1H, PhC***H***OO), δ 4.88, 4.84 (ABq, $\Delta\delta_{AB}$=0.04, J_{AB}=12.2Hz, 2H, Naph-C***H***$_2$-O), δ 4.50 (d, $J_{1,2}$=9.5Hz, 1H, H-1(Gal)), δ 4.28, 3.98 (ABdq, $\Delta\delta_{AB}$=0.30, J_{AB}=12.4Hz, $J_{5,6}$=1.7Hz, 2H, H-6(Gal)), δ 4.24 (dd, $J_{3,4}$=3.4Hz, $J_{4,5}$=0.8Hz, 1H, H-4(Gal)), δ 3.83 (dd, $J_{1,2}$=$J_{2,3}$=9.4Hz, 1H, H-2(Gal)), δ 3.56 (dd, $J_{2,3}$=9.3Hz, $J_{3,4}$=3.4Hz, 1H, H-3(Gal)), δ 3.45 (m, 1H, H-5(Gal)), δ 2.58-2.46 (w, 1H, 2-OH (Gal)), δ 2.35 (s, 3H, S-Ph-C***H***$_3$). ^{13}C{^{1}H} NMR (126MHz, CD_2Cl_2): δ 138.9 (*p*-Tol), δ 138.5 (*i*-Tol), δ 136.1 (Naph), δ 134.2 (*o*-Tol), δ 133.6 (Naph), δ 133.4 (Naph), δ 130.1 (*m*-Tol), δ 129.5 (PhCHOO), δ 128.5 (PhCHOO), δ 128.2 (Naph), δ 128.0 (Naph), δ 127.8 (PhCHOO), δ 126.9 (Naph), δ 126.9 (PhCHOO), δ 126.5 (Naph), δ 126.4 (Naph), δ 126.3 (Naph), δ 101.5 (Ph**C**HOO), δ 88.1 (C-1(Gal)), δ 80.8 (C-3(Gal)), δ 73.7 (C-4(Gal)), δ 72.1 (Naph-**C**H$_2$-O), δ 70.4 (C-5(Gal)), δ 69.8 (C-6(Gal)), δ 68.0 (C-2(Gal)), δ 21.3 (S-Ph-C***H***$_3$). The ^{1}H-NMR and ^{13}C-NMR spectra are in agreement with the literature.[2] Single crystals of **2a** were obtained from acetonitrile by slow evaporation. A suitable crystal was selected and measured on a XtaLAB Synergy, Single source at offset/far, HyPix diffractometer. The crystal was kept at 200.00(10) K during data collection. Using Olex2[5], the structure was solved with the SHELXT[6] structure solution program using Intrinsic Phasing and refined with the SHELXL[7] refinement package using Least Squares minimization. Crystal Data (CDCC 2173185) for $C_{31}H_{32}O_6S$ (M=532.62 g/mol): monoclinic, space group $P2_1$ (no. 4), *a*=15.4743(7) Å, *b*=4.98070(10) Å, *c*=18.4761(8) Å, *β*=112.767(5)°, *V*=1313.06(10) Å^3, Z=2, T=200.00(10) K, μ(Mo K*α*)=0.168 mm^{-1}, *Dcalc*=1.347 g/cm^3, 24847 reflections measured (4.522° ≤ 2Θ ≤ 63.976°), 7025 unique (R_{int}=0.0303, R_{sigma}=0.0324) which were used in all calculations. The final R_1 was 0.0349 ($I > 2\sigma(I)$) and wR_2 was 0.0818 (all data).

p-Tolyl 3-O-benzyl-4,6-O-benzylidene-1-thio-β-D-galactopyranoside (2b)

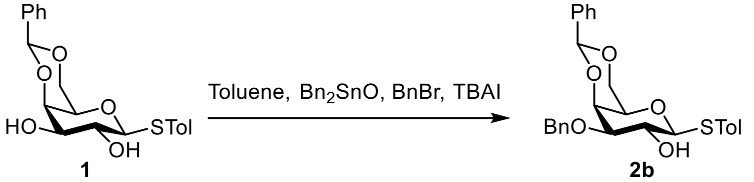

Compound **1** (230 mg, 0.62 mmol) and dibutyltin oxide (162 mg, 0.65 mmol) were suspended in dry toluene (10 ml). The reaction mixture was heated in an oil bath and refluxed for 3h in Dean-Stark apparatus equipped with a drying tube until no further water collection was observed. Reaction mixture was allowed to cool to 70°C and benzyl bromide (BnBr) (0.12 ml, 1.0 mmol) and tetrabutylammonium iodide (TBAI) (0.34 g, 0.93 mmol) were added. The reaction was left for 24 h. in an oil bath at 80°C until completion (as indicated by TLC, 70% EtOAc in hexane, R_f=0.66). The reaction mixture was concentrated *in vacuo* and purified by flash chromatography to obtain **2b** (132 mg, 46% yield, elution at 40% EtOAc in hexane). HRMS (ESI-TOF) m/z: $[M+Na]^+$ Calcd for $C_{27}H_{28}O_5NaS$ 487.1555; found 487.1555. ^{1}H NMR (500MHz, d_6-acetone): δ 7.55 (d, $J_{o,m}$=8.1Hz, 2H, *o*-Tol), δ 7.48-7.46 (m, 2H, *o*-PhCHOO), δ 7.38-7.35 (m, 5H, *o*-Bn and *m,p*-PhCHOO), δ 7.30-7.21 (m, 3H, *m,p*-Bn), δ 7.07 (d, $J_{o,m}$=7.8Hz, 2H, *m*-Tol), δ 5.61 (s, 1H, PhC***H***OO), δ 4.76, 4.69 (ABq, $\Delta\delta_{AB}$=0.07, J_{AB}=12.1Hz, 2H, Ph-C***H***$_2$-O), δ 4.61 (d, $J_{1,2}$=9.5Hz, 1H, H-1(Gal)), δ 4.49 (dd, $J_{3,4}$=3.4Hz, $J_{4,5}$=1.0Hz, 1H, H-4(Gal)), δ 4.27 (d, J_{OH}=4.2Hz, 1H, 2-OH (Gal)), δ 4.21, 4.11 (ABdq, $\Delta\delta_{AB}$=0.10, J_{AB}=12.3Hz, $J_{5,6}$=1.7Hz, 2H, H-6(Gal)), δ 3.82 (ddd, $J_{1,2}$=$J_{2,3}$=9.4Hz, J_{OH}=4.2Hz, 1H, H-2(Gal)), δ 3.66 (m, 1H, H-5(Gal)), δ 3.63 (dd, $J_{2,3}$=9.2Hz, $J_{3,4}$=3.4Hz, 1H, H-3(Gal)), δ 2.30 (s, 3H, S-Ph-C***H***$_3$), traces of residual EtOAc. ^{13}C{^{1}H} NMR (126MHz, d_6-acetone): δ 140.1 (PhCHOO), δ 140.0 (Bn), δ 138.0 (Tol), δ 133.8 (Tol), δ 130.2 (Tol), δ 130.2 (Tol), δ 129.4 (PhCHOO), δ 128.9 (Bn), δ 128.7 (PhCHOO), δ 128.3 (Bn), δ 128.0 (Bn), δ 127.4 (PhCHOO), δ 101.4 (Ph**C**HOO), δ 88.4 (C-1(Gal)), δ 82.0 (C-3(Gal)), δ 74.1 (C-4(Gal)), δ 71.7 (Ph-**C**H$_2$-O), δ 70.7 (C-5(Gal)), δ 70.1 (C-6(Gal)), δ 68.3 (C-2(Gal)), δ 21.1 (S-Ph-C***H***$_3$). The ^{1}H-NMR and ^{13}C-NMR spectra are in agreement with the literature.[3]

p-Tolyl 3-O-allyl-4,6-O-benzylidene-1-thio-β-D-galactopyranoside (2c)

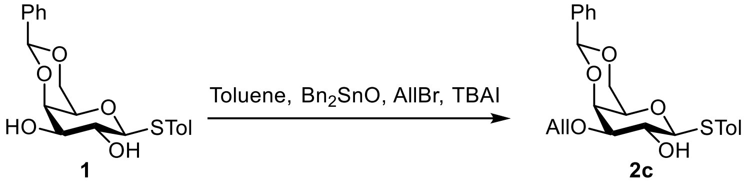

Compound **1** (1.06 g, 2.83 mmol) and dibutyltin oxide (0.73 g, 2.9 mmol) were suspended in dry toluene (30 ml). The reaction mixture was heated in an oil bath and refluxed for 3h in Dean-Stark apparatus equipped with a drying tube until no further water collection was observed. Reaction mixture was allowed to cool to RT and allyl bromide (AllBr) (0.87 ml, 10 mmol) and tetrabutylammonium iodide (TBAI) (1.57 g, 4.25 mmol) were added. The reaction was left for 2 days in an oil bath at 40°C (product formation indicated by TLC, 70% EtOAc in hexane, R_f=0.62). The reaction mixture was concentrated *in vacuo* and purified by flash chromatography to obtain **2c** (240 mg, 20% yield, elution at 50% EtOAc in hexane). HRMS (ESI-TOF) m/z: $[M+Na]^+$ Calcd for $C_{23}H_{26}O_5NaS$ 437.1399; found 437.1400. ^{1}H NMR (500MHz, d_6-acetone): δ 7.55 (d, $J_{o,m}$=8.2Hz, 2H, *o*-Tol), δ 7.46-7.44 (m, 2H, *o*-PhCHOO), δ 7.38-7.36 (m, 3H, *m,p*-PhCHOO), δ 7.07 (d, $J_{o,m}$=7.9Hz, 2H, *m*-Tol), δ 5.90 (ddt, J_{trans}=17.3Hz, J_{cis}=10.6Hz, J_{all}=5.3Hz, 1H, CH$_2$=C***H***-CH$_2$-O), δ 5.61 (s, 1H, PhC***H***OO), δ 5.26 (ddt, J_{trans}=17.3Hz, J_{gem}=1.8Hz, J_{all}=1.9Hz, 1H, C***H***$_2$=CH-CH$_2$-O), δ 5.06 (ddt, J_{cis}=10.5Hz, J_{gem}=2.0Hz, J_{all}=1.5Hz, 1H, C***H***$_2$=CH-CH$_2$-O), δ 4.59 (d, $J_{1,2}$=9.5Hz, 1H, H-1(Gal)), δ 4.44 (dd, $J_{3,4}$=3.4Hz, $J_{4,5}$=1.0Hz, 1H, H-4(Gal)), δ 4.21, 4.11 (ABdq, $\Delta\delta_{AB}$=0.10, J_{AB}=12.3Hz, $J_{5,6}$=1.7Hz, 2H, H-6(Gal)), δ 4.17 (d, J_{OH}=4.1Hz, 1H, 2-OH (Gal)), δ 4.21-4.13 (m, 2H, CH$_2$=CH-

C$\boldsymbol{H}_2$-O), δ 3.76 (ddd, $J_{1,2}$=$J_{2,3}$=9.3Hz, J_{OH}=4.2Hz, 1H, H-2(Gal)), δ 3.65 (m, 1H, H-5(Gal)), δ 3.53 (dd, $J_{2,3}$=9.2Hz, $J_{3,4}$=3.4Hz, 1H, H-3(Gal)), δ 2.31 (s, 3H, S-Ph-C$\boldsymbol{H}_3$). ^{13}C{^{1}H} NMR (126MHz, d_6-acetone): δ 140.0 (PhCHOO), δ 138.0 (Tol), δ 136.6 (CH$_2$=**C**H-CH$_2$-O), δ 133.8 (Tol), δ 130.2 (Tol), δ 130.1 (Tol), δ 129.4 (PhCHOO), δ 128.6 (PhCHOO), δ 127.4 (PhCHOO), δ 116.2 (**C**H$_2$=CH-CH$_2$-O), δ 101.5 (Ph**C**HOO), δ 88.4 (C-1(Gal)), δ 81.7 (C-3(Gal)), δ 74.3 (C-4(Gal)), δ 71.0 (Ph-**C**H$_2$-O), δ 70.8 (C-5(Gal)), δ 70.1 (C-6(Gal)), δ 68.3 (C-2(Gal)), δ 21.1 (S-Ph-C$\boldsymbol{H}_3$).

_p_-Tolyl 4,6-O-benzylidene-3-O-(cyclohexylmethyl)-1-thio-β-D-galactopyranoside (2d)

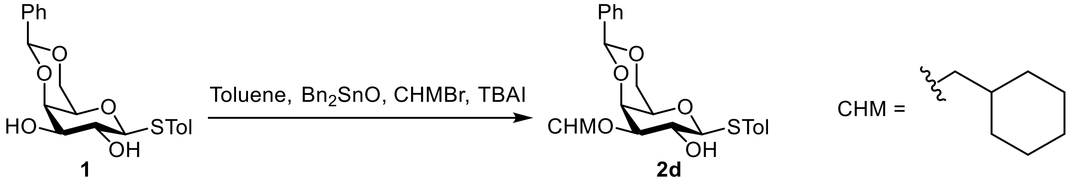

Compound **1** (1.23 g, 3.28 mmol) and dibutyltin oxide (0.86 g, 3.4 mmol) were suspended in dry toluene (43 ml). The reaction mixture was heated in an oil bath and refluxed for 3h in Dean-Stark apparatus equipped with a drying tube until no further water collection was observed. Reaction mixture was allowed to cool to 50°C and cyclohexylmethyl bromide (CHMBr) (0.69 ml, 4.9 mmol) and tetrabutylammonium iodide (TBAI) (1.82 g, 4.92 mmol) were added. The reaction was left overnight in an oil bath at 60°C. The reaction mixture was concentrated *in vacuo* and purified by flash chromatography to obtain **2d** (38 mg, 3% yield, elution at 40% EtOAc in hexane). HRMS (ESI-Q-Orbitrap) m/z: $[M+H]^+$ Calcd for $C_{27}H_{35}O_5S$ 471.2200; found 471.2203. ^{1}H NMR (500MHz, d_6-acetone): δ 7.55 (d, $J_{o,m}$=8.1Hz, 2H, *o*-Tol), δ 7.47-7.45 (m, 2H, *o*-PhCHOO), δ 7.40-7.35 (m, 3H, *m,p*-PhCHOO), δ 7.06 (d, $J_{o,m}$=7.8Hz, 2H, *m*-Tol), δ 5.61 (s, 1H, PhC**H**OO), δ 4.58 (d, $J_{1,2}$=9.5Hz, 1H, H-1(Gal)), δ 4.45 (dd, $J_{3,4}$=3.4Hz, $J_{4,5}$=1.0Hz, 1H, H-4(Gal)), δ 4.21, 4.11 (ABdq, $\Delta\delta_{AB}$=0.10, J_{AB}=12.2Hz, $J_{5,6}$=1.8Hz, 2H, H-6(Gal)), δ 4.07 (d, J_{OH}=4.1Hz, 1H, 2-OH (Gal)), δ 3.72 (ddd, $J_{1,2}$=$J_{2,3}$=9.4Hz, J_{OH}=4.1Hz, 1H, H-2(Gal)), δ 3.64 (m, 1H, H-5(Gal)), δ 3.45, 3.35 (ABdq, $\Delta\delta_{AB}$=0.10, J_{AB}=9.1Hz, J_{Cy}=6.3Hz, 2H, Cy-C$\boldsymbol{H}_2$-O), δ 3.43 (dd, $J_{2,3}$=9.3Hz, $J_{3,4}$=3.4Hz, 1H, H-3(Gal)), δ 2.30 (s, 3H, S-Ph-C$\boldsymbol{H}_3$), δ 1.77-1.72 (m, 2H, ***Cy***-CH$_2$-O), δ 1.67-1.59 (m, 3H, ***Cy***-CH$_2$-O), δ 1.56-1.47 (m, 1H, ***Cy***-CH$_2$-O), δ 1.24-1.08 (m, 3H, ***Cy***-CH$_2$-O), δ 0.95-0.86 (m, 2H, ***Cy***-CH$_2$-O). ^{13}C{^{1}H} NMR (126MHz, d_6-acetone): δ 140.0 (PhCHOO), δ 137.9 (Tol), δ 133.7 (Tol), δ 130.2 (Tol), δ 130.2 (Tol), δ 129.4 (PhCHOO), δ 128.6 (PhCHOO), δ 127.4 (PhCHOO), δ 101.4 (Ph**C**HOO), δ 88.4 (C-1(Gal)), δ 82.6 (C-3(Gal)), δ 75.7 (Cy-**C**H$_2$-O), δ 73.9 (C-4(Gal)), δ 70.8 (C-5(Gal)), δ 70.1 (C-6(Gal)), δ 68.1 (C-2(Gal)), δ 39.1 (***Cy***-CH$_2$-O), δ 30.7 (***Cy***-CH$_2$-O), δ 30.6 (***Cy***-CH$_2$-O), δ 27.3 (***Cy***-CH$_2$-O), δ 26.5 (***Cy***-CH$_2$-O), δ 26.5 (***Cy***-CH$_2$-O), δ 21.1 (S-Ph-C$\boldsymbol{H}_3$).

_p_-Tolyl 3-O-benzoyl-4,6-O-benzylidene-1-thio-β-D-galactopyranoside (2e)

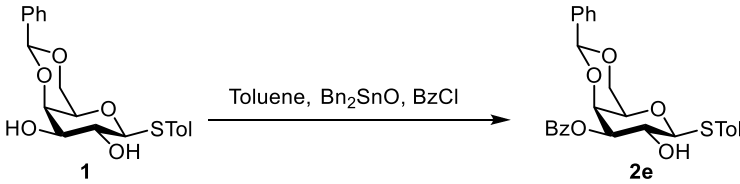

Compound **1** (2.87 g, 7.65 mmol) and dibutyltin oxide (2.0 g, 8.0 mmol) were suspended in dry toluene (95 ml). The reaction mixture was heated in an oil bath and refluxed for 3h in Dean-Stark apparatus equipped with a drying tube until no further water collection was observed. Reaction mixture was cooled to 0°C in an ice bath and benzoyl chloride (BzCl) (1.0 ml, 8.4 mmol) was added. The reaction was stirred for 2 h. at RT until completion (as indicated by TLC, 70% EtOAc in hexane, R_f=0.71). The reaction was quenched by the addition of isopropyl alcohol (~5 ml) and a few drops of sat. $NaHCO_3$ solution. A precipitate immediately formed. The reaction mixture was vacuum filtered, and the filtrate was concentrated *in vacuo*. The crude product was dissolved in minimal amount of EtOAc. Compound **2e** (1.61 g, 44% yield) was crystallized

from EtOAc and hexane. HRMS (ESI-TOF) m/z: $[M+Na]^+$ Calcd for $C_{27}H_{26}O_6NaS$ 501.1348; found 501.1349. ^{1}H NMR (500MHz, CD_2Cl_2): δ 8.02-8.01 (m, 2H, *o*-Bz), δ 7.59-7.55 (m, 3H, *o*-Tol and *p*-Bz), δ 7.44-7.36 (m, 7H, *o,m,p*-PhCHOO and *m*-Bz), δ 7.13 (d, $J_{o,m}$=7.9Hz, 2H, *m*-Tol), δ 5.50 (s, 1H, PhC***H***OO), δ 5.19 (dd, $J_{2,3}$=9.8Hz, $J_{3,4}$=3.5Hz, 1H, H-3(Gal)), δ 4.66 (d, $J_{1,2}$=9.5Hz, 1H, H-1(Gal)), δ 4.49 (dd, $J_{3,4}$=3.4Hz, $J_{4,5}$=0.7Hz, 1H, H-4(Gal)), δ 4.34, 4.06 (ABdq, $\Delta\delta_{AB}$=0.28, J_{AB}=12.4Hz, $J_{5,6}$=1.7Hz, 2H, H-6(Gal)), δ 4.05 (ddd, $J_{1,2}$=$J_{2,3}$=9.6Hz, J_{OH}=2.3Hz, 1H, H-2(Gal)), δ 3.69 (m, 1H, H-5(Gal)), δ 2.48 (d, J_{OH}=2.8Hz, 1H, 2-OH (Gal)), δ 2.36 (s, 3H, S-Ph-C***H***$_3$), traces of residual EtOAc (~1:0.1 ratio, δ 4.08 (q), δ 2.01 (s), δ 1.23 (t)). $^{13}C\{^1H\}$ NMR (126MHz, CD_2Cl_2): δ 166.5 (Ph**C**=OO), δ 139.0 (*i*-Tol), δ 138.4 (Ar), δ 134.1 (*o*-Tol), δ 133.6 (Bz), δ 130.1 (Ph**C**HOO), δ 130.1 (*m*-Tol), δ 130.0 (*o*-Bz), δ 128.8 (Ar), δ 128.4 (Ar), δ 127.4 (*p*-Tol), δ 126.7 (Ar), δ 101.1 (Ph**C**HOO), δ 88.6 (C-1(Gal)), δ 75.6 (C-3(Gal)), δ 74.4 (C-4(Gal)), δ 70.3 (C-5(Gal)), δ 69.5 (C-6(Gal)), δ 66.4 (C-2(Gal)), δ 21.3 (S-Ph-C***H***$_3$).). The ^{1}H-NMR spectrum is in agreement with the literature.[4]

***p*-Tolyl 2-O-benzoyl-4,6-O-benzylidene-3-O-(2-naphthalenylmethyl)-1-thio-β-D-galactopyranoside (3a)**

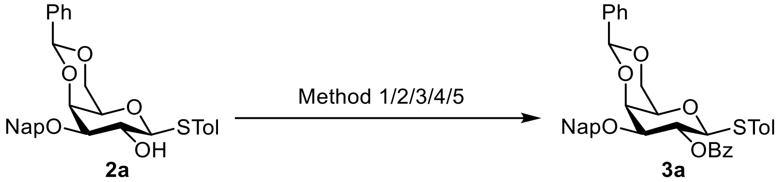

Compound **3a** was synthesized from **2a** following either one of the methods described below.

Method 1: Compound **2a** (62 mg, 0.12 mmol) was dissolved in dry DCM and pyridine (7 ml, 1:1) and cooled to 0°C in an ice bath under Ar. 4-(Dimethylamino)pyridine (DMAP) (1-2 crystals) and benzoic anhydride (Bz_2O) (55 mg, 0.24 mmol) were added. Reaction was left for 24h at RT. The reaction mixture was diluted with DCM (~20 ml) and quenched by the addition of aqueous HCl (0.1 M, 10 ml). The organic layer was separated and washed with HCl (0.1 M), then sat. $NaHCO_3$ (x3) and brine. The organic layer was dried over Na_2SO_4, gravitationally filtered and evaporated. Compound **3a** was purified by prep-HPLC (28 mg, 38% yield, elution at 95% ACN).

Method 2: Compound **2a** (118 mg, 0.229 mmol) was dissolved in dry N,N-dimethylformamide (20 ml). The reaction was cooled to 0°C in an ice bath under Ar flow, and NaH (60% dispersion in mineral oil, 11 mg, 0.275 mmol) added in one portion. The reaction mixture was stirred at 0°C for 15 min. until H_2 evolution stopped, then BzCl (32 µl, 0.28 mmol) was added. The mixture was stirred overnight at RT. Reaction mixture was diluted with DCM (50 ml) and poured onto aqueous HCl (0.5 M). The organic layer was separated and washed with HCl (0.1 M), then sat. $NaHCO_3$ (x2) and brine. The organic layer was dried over Na_2SO_4, gravitationally filtered and evaporated. Compound **3a** was purified by prep-HPLC (111 mg, 78% yield, elution at 95% ACN).

Method 3: Compound **2a** (53 mg, 0.10 mmol) was dissolved in dry DCM (5 ml) and cooled to 0°C in an ice bath under Ar. Triethylamine (TEA) (0.2 mmol, 0.03 ml) and 1,8-Diazabicyclo[5.4.0]undec-7-ene (DBU) (0.05 mmol, 8 µl) were added. After stirring for 10 min, BzCl (0.2 mmol, 0.02 ml) was added. Reaction was left for 24h at RT. The reaction mixture was diluted with DCM (~20 ml) and quenched by the addition of aqueous HCl (0.1 M, 10 ml). The organic layer was separated and washed with HCl (0.1 M), then sat. $NaHCO_3$ (x3) and brine. The organic layer was dried over Na_2SO_4, gravitationally filtered and evaporated. Compound **3a** was purified by prep-HPLC (21 mg, 33% yield, elution at 95% ACN).

Method 4: Compound **2a** (61 mg, 0.12 mmol) was dissolved in dry DCM (8 ml) and cooled to 0°C in an ice bath under Ar. TEA (0.2 mmol, 0.03 ml) was added. After stirring for 10 min, BzCl (0.2 mmol, 0.02 ml) was added. Reaction was left for 24h at RT. The reaction mixture was diluted with DCM (~20 ml) and quenched by the addition of aqueous HCl (0.1 M, 10 ml). The organic layer was separated and washed with HCl (0.1 M), then sat. $NaHCO_3$ (x3) and brine. The organic layer

was dried over Na_2SO_4, gravitationally filtered and evaporated. Compound **3a** was purified by prep-HPLC (9 mg, 12% yield, elution at 95% ACN).

Method 5: Compound **2a** (48 mg, 0.09 mmol) was dissolved in dry DCM and pyridine (6 ml, 1:1) and cooled to 0°C in an ice bath under Ar. TEA (0.2 mmol, 0.03 ml) and DMAP (1-2 crystals) were added. After stirring for 10 min, BzCl (0.2 mmol, 0.02 ml) was added. Reaction was left for 24h at RT. The reaction mixture was diluted with DCM (~20 ml) and quenched by the addition of aqueous HCl (0.1 M, 10 ml). The organic layer was separated and washed with HCl (0.1 M), then sat. $NaHCO_3$ (x3) and brine. The organic layer was dried over Na_2SO_4, gravitationally filtered and evaporated. Compound **3a** was purified by prep-HPLC (53 mg, 92% yield, elution at 95% ACN).

Compound **3a** was characterized after purification: HRMS (ESI-TOF) m/z: $[M+Na]^+$ Calcd for $C_{38}H_{34}O_6NaS$ 641.1974; found 641.1974. ^1H NMR (500MHz, d_6-acetone): δ 8.05-8.04 (m, 2H, *o*-Bz), δ 7.81-7.79 (m, 1H, Ar), δ 7.71 (m, 1H, Ar), δ 7.68-7.64 (m, 2H, Ar), δ 7.59-7.57 (m, 1H, Ar), δ 7.54-7.50 (m, 4H, Ar), δ 7.47-7.45 (m, 2H, *o*-Tol), δ 7.44-7.37 (m, 5H, Ar), δ 7.32 (dd, J_o=8.5Hz, J_m=1.7Hz, 1H, Ar), δ 7.11 (m, 2H, *m*-Tol), δ 5.70 (s, 1H, PhC***H***OO), δ 5.61 (dd, $J_{1,2}$=$J_{2,3}$=9.8Hz, 1H, H-2(Gal)), δ 5.02 (d, $J_{1,2}$=9.9Hz, 1H, H-1(Gal)), δ 4.88, 4.76 (ABq, $\Delta\delta_{AB}$=0.12, J_{AB}=12.6Hz, 2H, Naph-C$\boldsymbol{H_2}$-O), δ 4.73 (dd, $J_{3,4}$=3.5Hz, $J_{4,5}$=0.9Hz, 1H, H-4(Gal)), δ 4.27, 4.17 (ABdq, $\Delta\delta_{AB}$=0.10, J_{AB}=12.3Hz, $J_{5,6}$=1.6Hz, 2H, H-6(Gal)), δ 4.11 (dd, $J_{2,3}$=9.6Hz, $J_{3,4}$=3.5Hz, 1H, H-3(Gal)), δ 3.81 (m, 1H, H-5(Gal)), δ 2.30 (s, 3H, S-Ph-C$\boldsymbol{H_3}$). $^{13}C\{^1H\}$ NMR (126MHz, d_6-acetone): δ 165.6 (Ph**C**=OO), δ 139.7 (Ar), δ 138.3 (*i*-Tol), δ 136.9 (Ar), δ 134.1 (Ar), δ 134.0 (*m*-Bz), δ 133.8 (Ar), δ 133.6 (*o*-Tol), δ 131.4 (Ar), δ 130.4 (*o*-Bz), δ 130.3 (*m*-Tol), δ 130.1 (*p*-Tol), δ 129.5 (Ar), δ 129.4 (Ar), δ 128.7 (Ar), δ 128.6 (Ar), δ 128.5 (Ar), δ 128.4 (Ar), δ 127.4 (Ar), δ 126.8 (Ar), δ 126.6 (Ar), δ 126.5 (Ar), δ 101.6 (Ph**C**HOO), δ 86.3 (C-1(Gal)), δ 79.4 (C-3(Gal)), δ 73.6 (C-4(Gal)), δ 71.0 (Naph-**C**H_2-O), δ 70.9 (C-5(Gal)), δ 69.9 (C-2(Gal)), δ 70.1 (C-6(Gal)), δ 21.1 (S-Ph-C$\boldsymbol{H_3}$). The ^1H-NMR and ^{13}C-NMR spectra are in agreement with the literature.[2]

General procedure for DMAP catalyzed benzoylation (3)

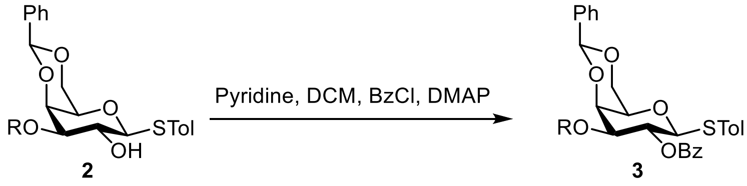

Compound **2a/b/c/d/e** (1 mmol) was dissolved in dry DCM and dry pyridine (70 ml, 1:1). Reaction cooled to 0°C in an ice bath and DMAP (3-4 crystals) and BzCl (2 mmol) added. Reaction was left for 24 h. at RT. The reaction mixture was diluted with DCM (100 ml) and quenched by the addition of aqueous HCl (0.1 M, 20 ml). The organic layer was separated and washed with HCl (0.1 M), then sat. $NaHCO_3$ (x3) and brine. The organic layer was dried over Na_2SO_4, gravitationally filtered and evaporated. The crude product was analyzed by analytical HPLC.

p-Tolyl 3-O-allyl-2-O-benzoyl-4,6-O-benzylidene-1-thio-β-D-galactopyranoside (3c)

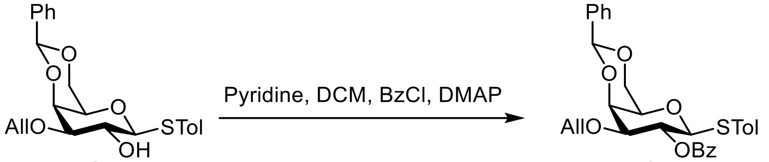

2c **3c**

Compound **2c** (240 mg, 0.58 mmol) was dissolved in dry DCM and dry pyridine (30 ml, 1:1). Reaction cooled to 0°C in an ice bath and DMAP (1-2 crystals) and BzCl (0.14 ml, 1.2 mmol) added. Reaction was left for 24 h. at RT. The reaction mixture was diluted with DCM (~50 ml) and quenched by the addition of aqueous HCl (0.1 M, 10 ml). The organic layer was separated and washed with HCl (0.1 M), then sat. $NaHCO_3$ (x3) and brine. The organic layer was dried over Na_2SO_4, gravitationally filtered and evaporated. The crude product was purified by prep-HPLC (196 mg, 65% yield, elution at 93% ACN). HRMS (ESI-TOF) m/z: $[M+Na]^+$ Calcd for $C_{30}H_{30}O_6NaS$ 541.1661; found 541.1661. ^1H NMR (500MHz, d_6-acetone): δ 8.07-8.05 (dd, J_m=1.1 Hz, J_o=8.3 Hz, 2H, *o*-Bz), δ 7.43 (m, 1H, *p*-Bz), δ 7.55-7.52 (m, 2H, *m*-PhCHOO), δ 7.50-7.48 (m, 2H, Ar), δ 7.46 (d, $J_{o,m}$=8.2Hz, 2H, *o*-Tol), δ 7.41-7.37 (m, 3H, Ar), δ 7.07 (d, $J_{o,m}$=8.2Hz, 2H, *m*-Tol), δ 5.75 (ddt, J_{trans}=17.2Hz, J_{cis}=10.6Hz, J_{all}=5.3Hz, 1H, CH_2=C***H***-CH_2-O), δ 5.68 (s, 1H, PhC***H***OO), δ 5.52 (dd, $J_{1,2}$=$J_{2,3}$=9.8Hz, 1H, H-2(Gal)), δ 5.16 (ddt, J_{trans}=17.3Hz, J_{gem}=1.7Hz, J_{all}=1.7Hz, 1H, C***H***$_2$=CH-CH_2-O), δ 5.02 (d, $J_{1,2}$=9.9Hz, 1H, H-1(Gal)), δ 4.98 (ddt, J_{cis}=10.5Hz, J_{gem}=1.5Hz, J_{all}=1.5Hz, 1H, C***H***$_2$=CH-CH_2-O), δ 4.62 (m, 1H, H-4(Gal)), δ 4.27, 4.17 (ABdq, $\Delta\delta_{AB}$=0.10, J_{AB}=12.3Hz, $J_{5,6}$=1.7Hz, 2H, H-6(Gal)), δ 4.19-4.01 (m, 2H, CH_2=CH-C***H***$_2$-O), δ 4.00 (dd, $J_{2,3}$=9.7Hz, $J_{3,4}$=3.5Hz, 1H, H-3(Gal)), δ 3.81 (m, 1H, H-5(Gal)), δ 2.31 (s, 3H, S-Ph-C***H***$_3$). $^{13}C\{^1H\}$ NMR (126MHz, d_6-acetone): δ 165.6 (Ph**C**=OO), δ 139.7 (PhCHOO), δ 138.3 (Tol), δ 135.9 (CH_2=**C**H-CH_2-O), δ 133.9 (Bz), δ 133.6 (Tol), δ 131.4 (Bz), δ 130.3 (Bz), δ 130.3 (Tol), δ 130.1 (Tol), δ 129.5 (PhCHOO), δ 129.4 (Bz), δ 128.7 (PhCHOO), δ 127.4 (PhCHOO), δ 116.6 (**C**H$_2$=CH-CH_2-O), δ 101.6 (Ph**C**HOO), δ 86.4 (C-1(Gal)), δ 79.6 (C-3(Gal)), δ 74.0 (C-4(Gal)), δ 70.9 (C-5(Gal)), δ 70.5 (Ph-**C**H$_2$-O), δ 70.2 (C-2(Gal)), δ 69.9 (C-6(Gal)), δ 21.1 (S-Ph-C***H***$_3$).

p-Tolyl 2,3-O-dibenzoyl-4,6-O-benzylidene-1-thio-β-D-galactopyranoside (3e)

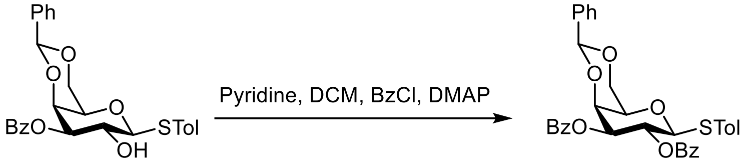

2e **3e**

Compound **2e** (366 mg, 0.76 mmol) was dissolved in dry DCM and dry pyridine (30 ml, 1:1). Reaction cooled to 0°C in an ice bath and DMAP (1-2 crystals) and BzCl (0.18 ml, 1.5 mmol) added. Reaction was left for 24 h. at RT. The reaction mixture was diluted with DCM (~50 ml) and quenched by the addition of aqueous HCl (0.1 M, 10 ml). The organic layer was separated and washed with HCl (0.1 M), then sat. $NaHCO_3$ (x3) and brine. The organic layer was dried over Na_2SO_4, gravitationally filtered and evaporated. The crude product was purified by prep-HPLC (368 mg, 83% yield, elution at 95% ACN). HRMS (ESI-TOF) m/z: $[M+Na]^+$ Calcd for $C_{34}H_{30}O_7NaS$ 605.1610; found 605.1609. ^1H NMR (500MHz, d_6-acetone): δ 7.98-7.96 (m, 2H, *o*-Bz), δ 7.89-7.87 (m, 2H, *o*-Bz), δ 7.60-7.57 (m, 1H, *p*-Bz), δ 7.56-7.53 (m, 1H, *p*-Bz), δ 7.51 (d, $J_{o,m}$=8.2Hz, 2H, *o*-Tol), δ 7.48-7.44 (m, 4H, Ar), δ 7.48-7.37 (m, 5H, Ar), δ 7.13 (d, $J_{o,m}$=8.4Hz, 2H, *m*-Tol), δ 5.75 (dd, $J_{1,2}$=$J_{2,3}$=9.9Hz, 1H, H-2(Gal)), δ 5.69 (s, 1H, PhC***H***OO), δ 5.59 (dd, $J_{2,3}$=9.9Hz, $J_{3,4}$=3.5Hz, 1H, H-3(Gal)), δ 5.27 (d, $J_{1,2}$=9.8Hz, 1H, H-1(Gal)), δ 4.74 (dd, $J_{3,4}$=3.5Hz, $J_{4,5}$=1.0Hz, 1H, H-4(Gal)), δ 4.34, 4.26 (ABdq, $\Delta\delta_{AB}$=0.08, J_{AB}=12.4Hz, $J_{5,6}$=1.7Hz, 2H, H-6(Gal)), δ 4.07 (m, 1H, H-5(Gal)), δ 2.35 (s, 3H, S-Ph-C***H***$_3$). $^{13}C\{^1H\}$ NMR (126MHz, d_6-acetone): δ 166.1 (Ph**C**=OO), δ 165.7 (Ph**C**=OO), δ 139.5 (Ar), δ 138.7 (Ar), δ 134.2 (Ar), δ 134.2 (Ar), δ 134.1 (*o*-Tol), δ 130.8 (Ar),

δ 130.4 (Ar), δ 130.4 (*m*-Tol), δ 130.3 (*p*-Bz), δ 130.3 (*p*-Bz), δ 129.6 (Ar), δ 129.4 (Ar), δ 129.2 (Ar), δ 128.8 (Ar), δ 127.4 (Ar), δ 101.5 (Ph**C**HOO), δ 85.7 (C-1(Gal)), δ 74.8 (C-3(Gal), C-4(Gal)), δ 69.7 (C-5(Gal)), δ 68.5 (C-6(Gal)), δ 69.9 (C-2(Gal)), δ 21.2 (S-Ph-C***H***$_3$). The ^{1}H-NMR spectrum is in agreement with the literature.[8]

_p_-Tolyl 3-O-(2-naphthalenylmethyl)-1-thio-β-D-galactopyranoside (4)

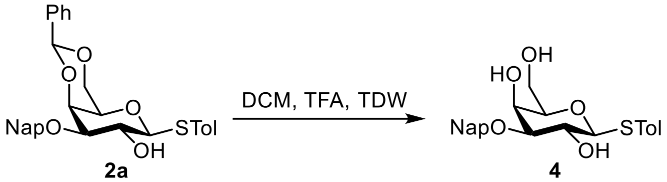

Compound **2a** (0.42 g, 0.82 mmol) was dissolved in DCM (20 ml) and cooled to 0°C in an ice bath. Trifluoroacetic acid (TFA) (2 ml) was added dropwise, followed by addition of TDW (0.1 ml). The reaction was allowed to reach RT and left for 1h until completion (as indicated by TLC, 70% EtOAc in hexane, R_f=0.38). The reaction mixture was diluted with DCM, and the organic layer was washed with sat. $NaHCO_3$. The aqueous layer was extracted with 1% MeOH in DCM (x3, 20 ml). The combined organic layer was washed with brine, dried over Na_2SO_4, gravitationally filtered and evaporated to yield an off-white solid. The crude product was triturated with 5% EtOAc in hexane (x3) to yield sufficiently pure **4** (310 mg, 89% yield). HRMS (ESI-Q-Orbitrap) m/z: $[M+H]^+$ Calcd for $C_{24}H_{27}O_5S$ 427.1574; found 427.1577. ^{1}H NMR (500MHz, d_6-acetone): δ 7.93-7.86 (m, 4H, Naph), δ 7.58 (dd, J_o=8.5Hz, J_m=1.7Hz, 2H, 3-Naph), δ 7.51-7.48 (m, 2H, Naph), δ 7.46 (d, $J_{o,m}$=8.2Hz, 2H, *o*-Tol), δ 7.12 (m, 2H, *m*-Tol), δ 4.96, 4.88 (ABq, $\Delta\delta_{AB}$=0.08, J_{AB}=12.2Hz, 2H, Naph-C***H***$_2$-O), δ 4.59 (d, $J_{1,2}$=9.7Hz, 1H, H-1(Gal)), δ 4.34 (d, J_{OH}=4.6Hz, 1H, 2-OH (Gal)), δ 4.25-4.23 (m, 1H, H-4(Gal)), δ 3.84 (ddd, $J_{1,2}$=$J_{2,3}$=9.3Hz, J_{OH}=4.7Hz, 1H, H-2(Gal)), δ 3.79-3.75 (m, 4H, H-6(Gal); 3-OH; 6-OH), δ 3.59 (m, 1H, H-5(Gal)), δ 3.56 (dd, $J_{2,3}$=9.0Hz, $J_{3,4}$=3.2Hz, 1H, H-3(Gal)), δ 2.29 (s, 3H, S-Ph-C***H***$_3$). ^{13}C{^{1}H} NMR (126MHz, d_6-acetone): δ 137.6 (Naph), δ 137.6 (Naph), δ 134.3 (Naph), δ 133.9 (Naph), δ 132.6 (*o*-Tol), δ 131.8 (Naph), δ 130.2 (*m*-Tol), δ 128.6 (Naph), δ 128.6 (Naph), δ 128.5 (Naph), δ 126.9 (Naph), δ 126.8 (Naph), δ 126.8 (Naph), δ 126.6 (Naph), δ 89.6 (C-1(Gal)), δ 83.8 (C-3(Gal)), δ 80.0 (C-5(Gal)), δ 72.1 (Naph-**C**H$_2$-O), δ 69.8 (C-2(Gal)), δ 67.2 (C-4(Gal)), δ 62.5 (C-6(Gal)), δ 21.0 (S-Ph-C***H***$_3$).

_p_-Tolyl 2,4,6-O-tribenzoyl-3-O-(2-naphthalenylmethyl)-1-thio-β-D-galactopyranoside (5)

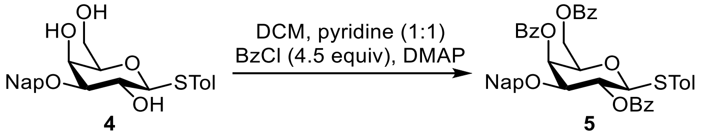

Compound **4** (320 mg, 0.75 mmol) was dissolved in dry DCM and dry pyridine (30 ml, 1:1). Reaction cooled to 0°C and a few crystals of DMAP and BzCl (0.39 ml, 3.4 mmol) added. Reaction left overnight at RT. The reaction mixture was diluted with DCM (50 ml) and quenched with aqueous HCl (0.1 M, 20 ml). The organic layer was separated and washed with HCl (0.1 M), then sat. $NaHCO_3$ (x3) and brine. The organic layer was dried over Na_2SO_4, gravitationally filtered and evaporated. Compound **5** was purified by flash chromatography (elution at 30% EtOAc in hexane, 554 mg, 74% yield). HRMS (ESI-Q-Orbitrap) m/z: $[M+NH_4]^+$ Calcd for $C_{45}H_{42}NO_8S$ 756.2626; found 756.2629. ^{1}H NMR (500MHz, $CDCl_3$): δ 8.06-8.02 (m, 4H, Ar), δ 7.97-7.95 (m, 2H, Ar), δ 7.69-7.68 (m, 1H, Ar), δ 7.63-7.58 (m, 4H, Ar), δ 7.50-7.41 (m, 11H, Ar), δ 7.40-7.36 (m, 1H, Ar), δ 7.18-7.16 (m, 2H, Ar), δ 6.99 (d, $J_{o,m}$=7.9Hz, 2H, *m*-Tol), δ 5.97 (dd, $J_{3,4}$=3.3Hz, $J_{4,5}$=0.8Hz, 1H, H-4(Gal)), δ 5.53(dd, $J_{1,2}$=$J_{2,3}$=9.8Hz, 1H, H-2(Gal)), δ 4.83, 4.64 (ABq, $\Delta\delta_{AB}$=0.19, J_{AB}=12.9Hz, 2H, Naph-C***H***$_2$-O), δ 4.76 (d, $J_{1,2}$=10.0Hz, 1H, H-1(Gal)), δ 4.59, 4.47 (ABdq, $\Delta\delta_{AB}$=0.12, J_{AB}=11.5Hz, J_{5,H_A}=7.3Hz, J_{5,H_B}=5.2Hz, 2H, H-6(Gal)), δ 4.13 (ddd, J_{5,H_A}=7.3Hz, J_{5,H_B}=5.3Hz, $J_{4,5}$=1.0Hz, 1H, H-5(Gal)), δ 3.87 (dd, $J_{2,3}$=9.5Hz, $J_{3,4}$=3.3Hz, 1H, H-3(Gal)), δ 2.31 (s, 3H,

S-Ph-C$\boldsymbol{H}_3$). $^{13}C\{^1H\}$ NMR (126MHz, $CDCl_3$): δ 166.1 (Ph**C**=OO), δ 165.8 (Ph**C**=OO), δ 165.1 (Ph**C**=OO), δ 138.2 (Ar), δ 134.5 (Ar), δ 133.9 (Ar), δ 133.4 (Ar), δ 133.2 (Ar), δ 133.1 (Ar), δ 133.0 (Ar), δ 132.9 (Ar), δ 130.2 (Ar), δ 129.9 (Ar), δ 129.9 (Ar), δ 129.8 (Ar), δ 129.6 (Ar), δ 129.5 (Ar), δ 129.2 (Ar), δ 129.0 (Ar), δ 128.5 (Ar), δ 128.4 (Ar), δ 128.4 (Ar), δ 128.2 (Ar), δ 128.2 (Ar), δ 127.8 (Ar), δ 127.6 (Ar), δ 126.9 (Ar), δ 126.0 (Ar), δ 125.9 (Ar), δ 125.9 (Ar), δ 86.3 (C-1(Gal)), δ 77.2 (C-3(Gal)), δ 75.1 (C-5(Gal)), δ 71.0 (Naph-**C**H_2-O), δ 69.4 (C-2(Gal)), δ 66.8 (C-4(Gal)), δ 63.1 (C-6(Gal)), δ 21.2 (S-Ph-C$\boldsymbol{H}_3$).

***p*-Tolyl 2-O-acetyl-4,6-O-benzylidene-3-O-(2-naphthalenylmethyl)-1-thio-β-D-galactopyranoside (6)**

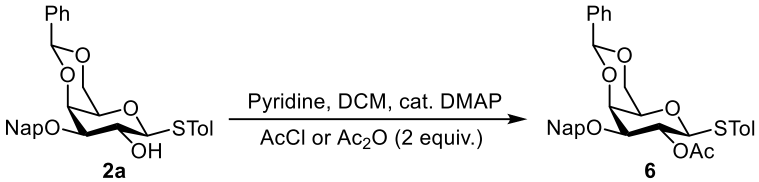

Compound **6** was synthesized from **2a** following either method 1 or method 2.

Method 1: Compound **2a** (90 mg, 0.17 mmol) was dissolved in dry DCM and pyridine (10 ml, 1:1). Reaction cooled to 0°C and DMAP (1-2 crystals) and AcCl (25 µl, 0.35 mmol) added. Reaction was left overnight at RT until completion (as indicated by RP-18 TLC, 70% ACN in TDW R_f=0.21). The reaction mixture was diluted with DCM (~30 ml) and quenched by the addition of aqueous HCl (0.1 M, 10 ml). The organic layer was separated and washed with HCl (0.1 M), then sat. $NaHCO_3$ (x3) and brine. The organic layer was dried over Na_2SO_4, gravitationally filtered and evaporated. Compound **6** was purified by prep-HPLC (81 mg, 83% yield, elution at 91% ACN).

Method 2: Compound **2a** (77 mg, 0.15 mmol) was dissolved in dry DCM and pyridine (7 ml, 1:1). Reaction cooled to 0°C and DMAP (1-2 crystals) and Ac_2O (0.03 ml, 0.3 mmol) added. Reaction was left overnight at RT. The reaction mixture was diluted with DCM (~30 ml) and quenched by the addition of aqueous HCl (0.1 M, 5 ml). The organic layer was separated and washed with HCl (0.1 M), then sat. $NaHCO_3$ (x3) and brine. The organic layer was dried over Na_2SO_4, gravitationally filtered and evaporated. Sufficiently pure **6** was afforded as an off-white solid without further purification (83 mg, 100% yield).

Compound **6** was characterized after purification: HRMS (ESI-Q-Orbitrap) m/z: $[M+Na]^+$ Calcd for $C_{33}H_{32}O_6NaS$ calculated 579.1812; found 579.1817. ^1H NMR (500MHz, $CDCl_3$): δ 7.83-7.75 (m, 3H, Naph), δ 7.72 (s, 1H, 1-Naph), δ 7.49 (d, $J_{o,m}$=8.1Hz, 2H, *o*-Tol), δ 7.49-7.46 (m, 2H, Ar), δ 7.43-7.40 (m, 3H, Ar), δ 7.36-7.33 (m, 3H, Ar), δ 7.05 (d, $J_{o,m}$=8.0Hz, 2H, *m*-Tol), δ 5.41 (s, 1H, PhC***H***OO), δ 5.29 (dd, $J_{1,2}$=$J_{2,3}$=9.7Hz, 1H, H-2(Gal)), δ 4.80, 4.76 (ABq, $\Delta\delta_{AB}$=0.04, J_{AB}=12.9Hz, 2H, Naph-C$\boldsymbol{H}_2$-O), δ 4.55 (d, $J_{1,2}$=9.8Hz, 1H, H-1(Gal)), δ 4.33, 3.95 (ABdq, $\Delta\delta_{AB}$=0.38, J_{AB}=12.3Hz, $J_{5,6}$=1.6Hz, 2H, H-6(Gal)), δ 4.18 (d, $J_{3,4}$=3.2Hz, 1H, H-4(Gal)), δ 3.64 (dd, $J_{2,3}$=9.6Hz, $J_{3,4}$=3.4Hz, 1H, H-3(Gal)), δ 3.40 (m, 1H, H-5(Gal)), δ 2.32 (s, 3H, S-Ph-C$\boldsymbol{H}_3$), δ 2.09 (s, 3H, Ac). $^{13}C\{^1H\}$ NMR (126MHz, $CDCl_3$): δ 169.2 (CH_3**C**=OO), δ 138.1 (*i*-Tol), δ 137.6 (Ar), δ 134.2 (*o*-Tol), δ 133.1 (Ar), δ 133.0 (Ar), δ 129.5 (*m*-Tol), δ 129.1 (Ar), δ 128.2 (Ar), δ 128.2 (Ar), δ 128.1 (Ar), δ 127.8 (*p*-Tol), δ 127.7 (Ar), δ 126.7 (*o*-PhCHOO), δ 126.4 (Ar), δ 126.2 (Ar), δ 126.0 (2-Naph), δ 125.6 (Ar), δ 125.3 (Ar), δ 101.3 (Ph**C**HOO), δ 85.4 (C-1(Gal)), δ 78.6 (C-3(Gal)), δ 73.2 (C-4(Gal)), δ 71.3 (Naph-**C**H_2-O), δ 69.9 (C-5(Gal)), δ 69.3 (C-6(Gal)), δ 68.4 (C-2(Gal)), δ 21.5 (S-Ph-C$\boldsymbol{H}_3$), δ 21.2 (**C**H_3C=OO).

Computational methods

In order to locate the transition states (TSs) for the benzoylation of **2a**-**2d**, we started with a small model system. The benzoyl group of the electrophile was replaced with an acryloyl group to obtain the electronically comparable 1-acryloyl-4-(dimethylamino)-pyridinium and the sugar backbone was truncated to obtain isopropyl alcohol while preserving the secondary nature of the alcohol (Fig. S1a). The TS was located using the QST2 method in Gaussian16 which requires optimized geometry of reactants and products (Fig. S1a and Fig.S1c).[9] The calculation resulted in the location of a TS with a single imaginary frequency (Fig. S1b). To further confirm the validity of the TS, an intrinsic reaction coordinate (IRC) calculation was performed to follow the reaction pathway from the TS to the reactants and products (Fig. S1d). The IRC calculation resulted in similar geometries as the reactants and products used in the QST2 calculation (Fig. S1e and Fig. S1f).

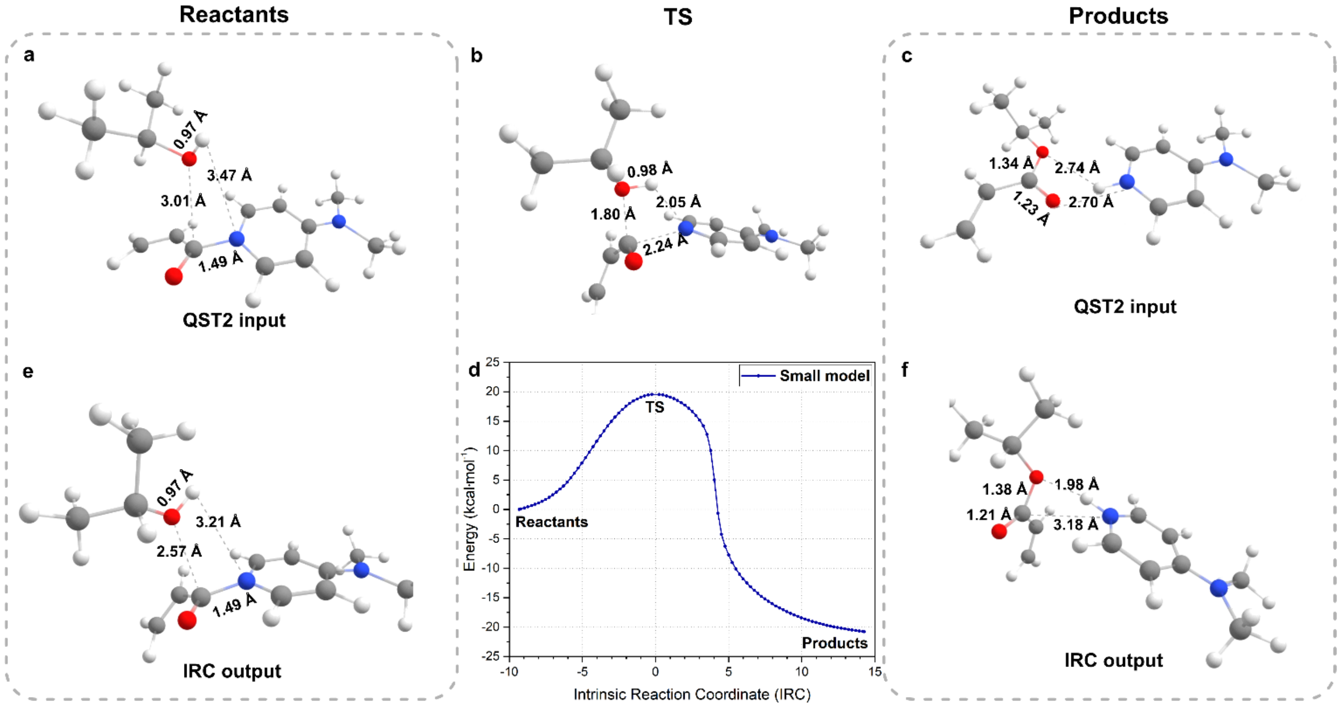

Figure S1. Optimized geometries of the a) Alcohol and acylpyridinium reactants, b) Transition state and c) Ester and 4-(dimethylamino)-pyridinium products of the simplified model system using B3LYP-D3BJ/6-31G(d,p). d) IRC calculation for the transition state of the small model system. Structures obtained from the IRC calculations of e) Reactants, f) Products.

The small model system was scaled-up systematically and incrementally to obtain the full-size models of 2a-2d without any truncations. First, the sugar backbone corresponding to the crystal structure of **2a** and the full-sized electrophile **E*** were recovered. Subsequently, the *O*-3-ether substituent was gradually increased in size (R= All, Bn, Nap, CHM). The pre-reaction complexes (PRCs) and the TSs were optimized after each addition. The motions associated with the TS and the overall '4-centered' geometry of the TS for the full-size models were comparable to the small model (Fig. S2).

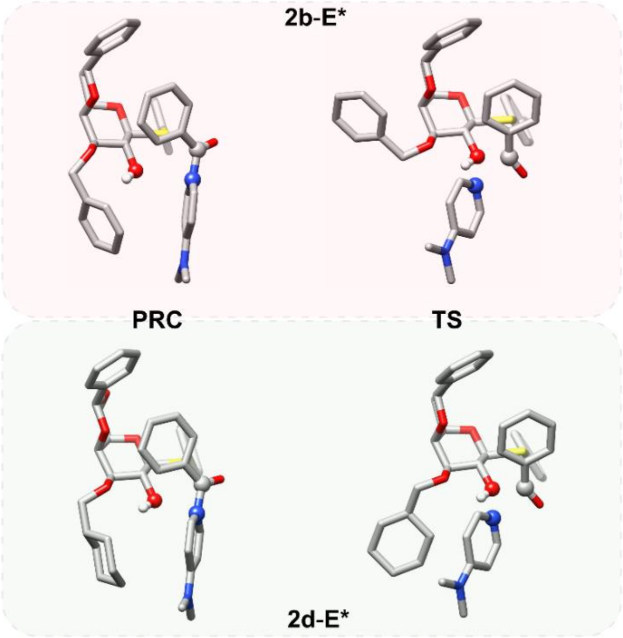

Figure S2. Optimized geometries of the PRC and TS for **2b** (top) and **2d** (bottom).

S3. Formation of side products in acetylation of **2a** with AcCl to form Compound **6**

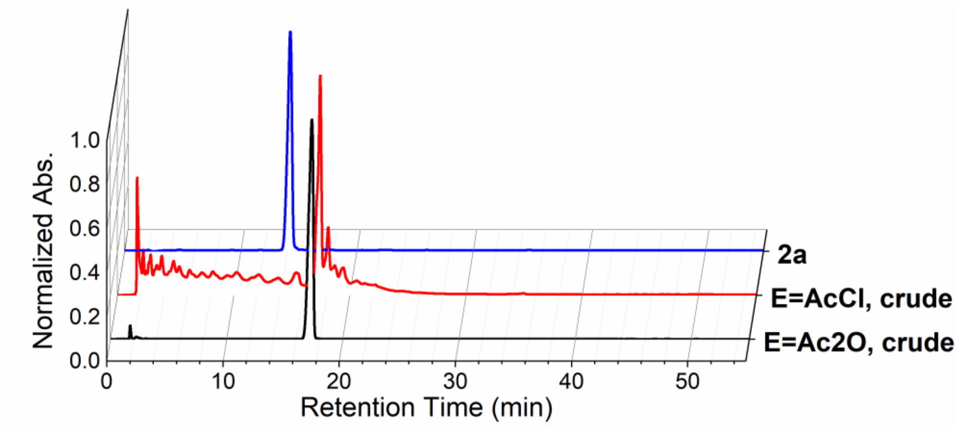

Figure S3. Analytical HPLC trace of crude **2a** acetylation product obtained by methods 1 and 2 with AcCl and Ac_2O electrophiles respectively.

S4. Comparative experiment for Compound **2e**

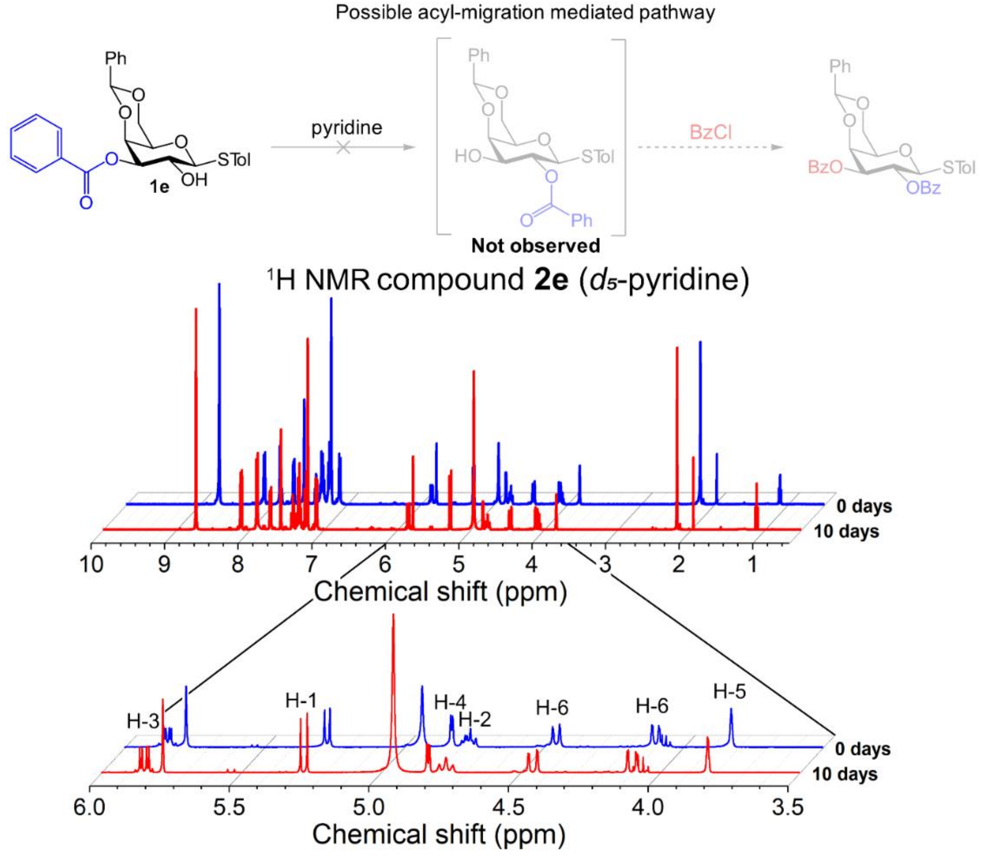

Figure S4. ^{1}H-NMR of **2e** in d_5-pyridine at time 0 and after 10 days.

Spectroscopic data

Compound **1**

^{1}H-NMR

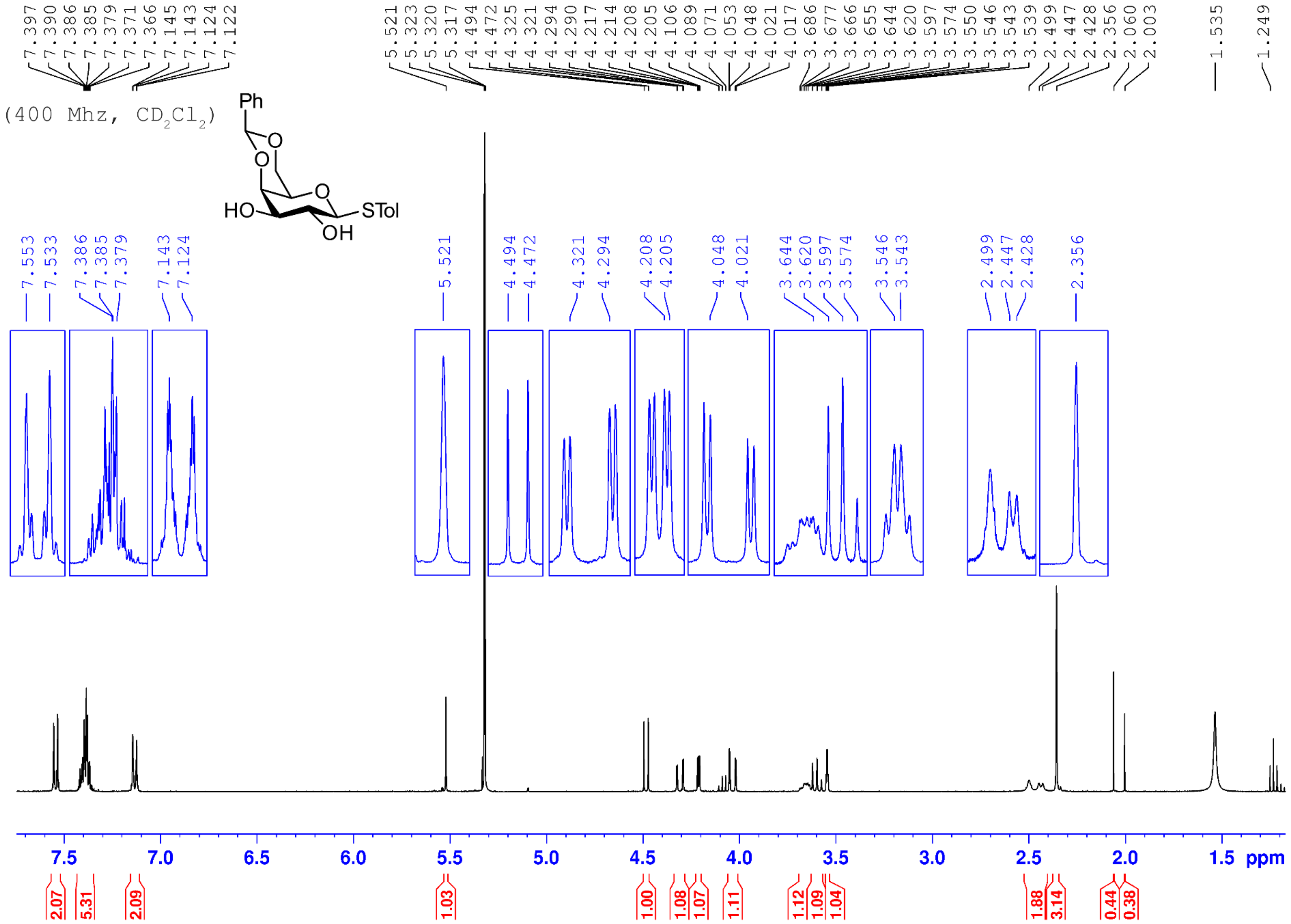

^{1}H-^{1}H COSY

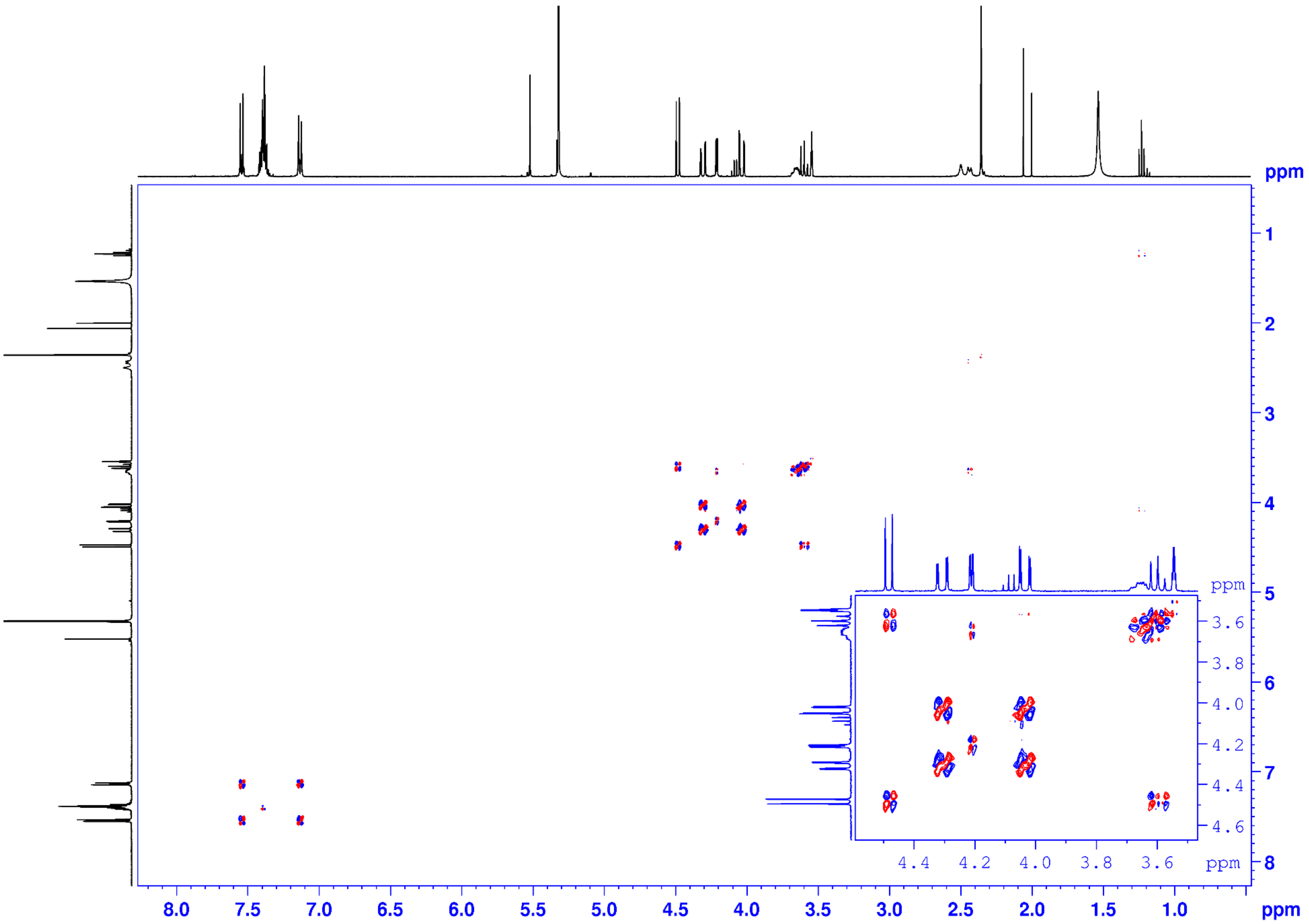

ppm
1
2
3
4
5
6
7
8
8.0
7.5
7.0
6.5
6.0
5.5
5.0
4.5
4.0
3.5
3.0
2.5
2.0
1.5
1.0
ppm
3.6
3.8
4.0
4.2
4.4
4.6
4.4
4.2
4.0
3.8
3.6
ppm

^1H-^{13}C HSQC

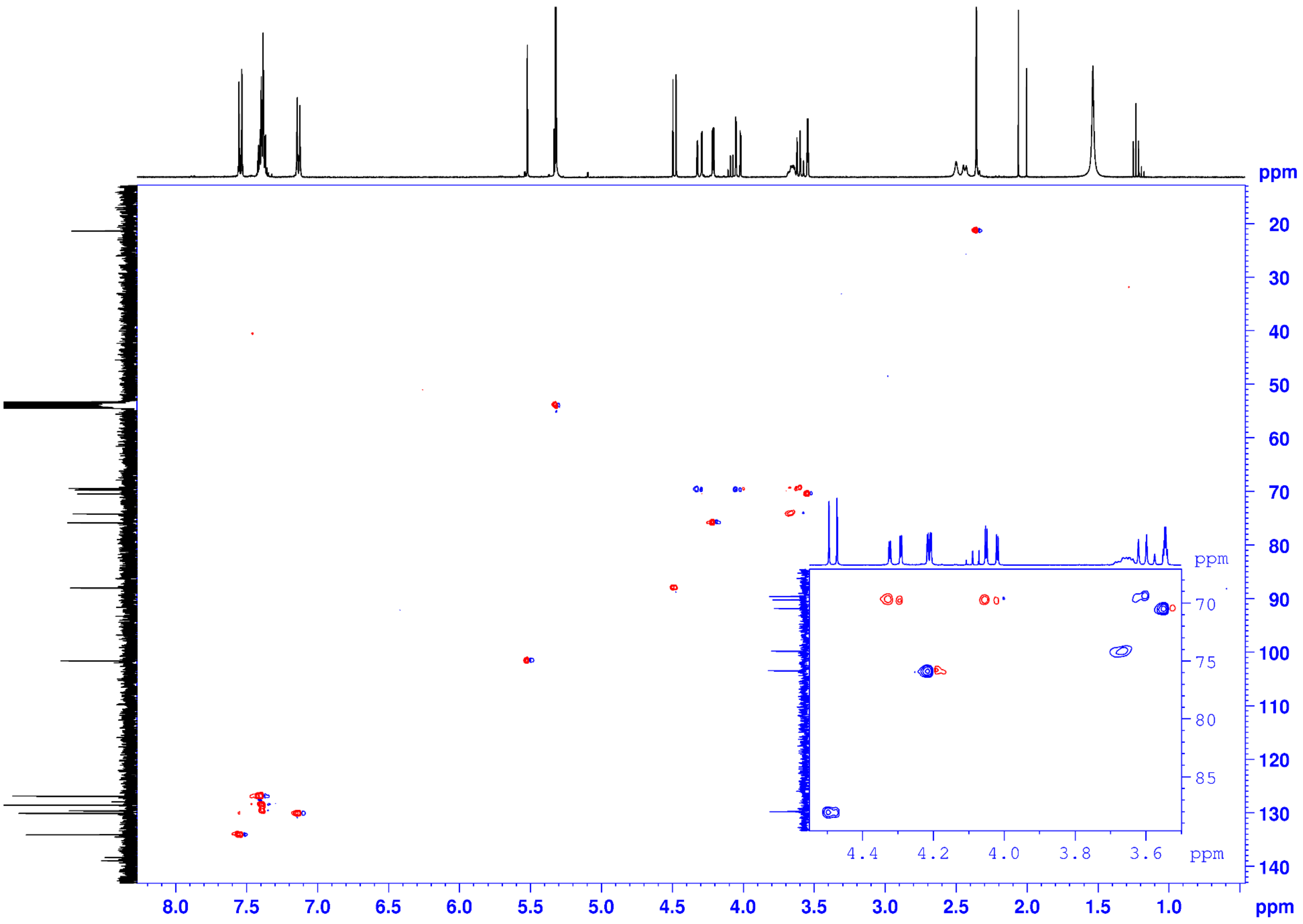
ppm
20
30
40
50
60
70
80
90
100
110
120
130
140
8.0
7.5
7.0
6.5
6.0
5.5
5.0
4.5
4.0
3.5
3.0
2.5
2.0
1.5
1.0
ppm
4.4
4.2
4.0
3.8
3.6
ppm
70
75
80
85
ppm

$^{13}C\{^1H\}$ NMR

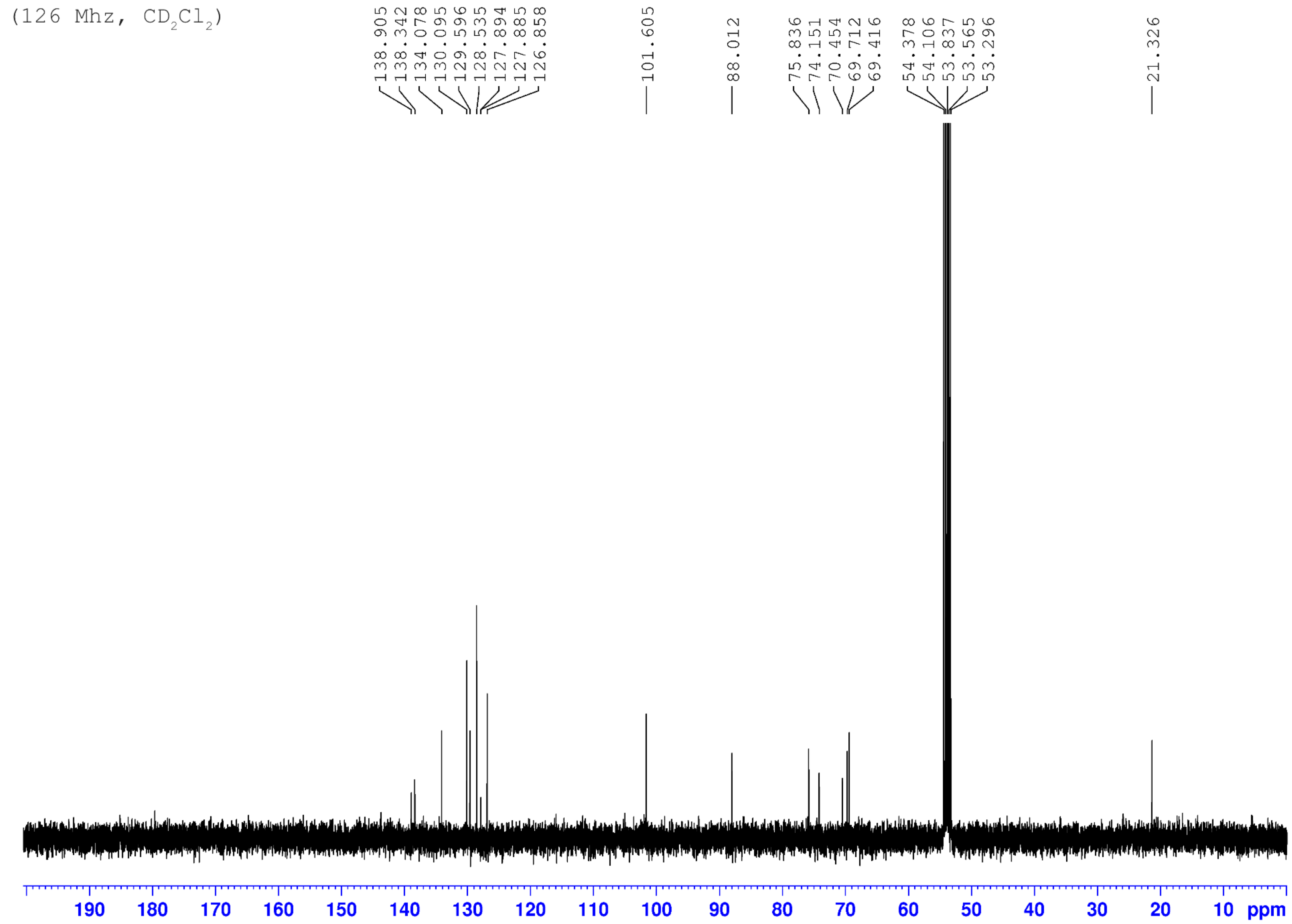
(126 Mhz, CD2Cl2)
138.905
138.342
134.078
130.095
129.596
128.535
127.894
127.885
126.858
101.605
88.012
75.836
74.151
70.454
69.712
69.416
54.378
54.106
53.837
53.565
53.296
21.326
190 180 170 160 150 140 130 120 110 100 90 80 70 60 50 40 30 20 10 ppm

Compound **2a**

^{1}H-NMR

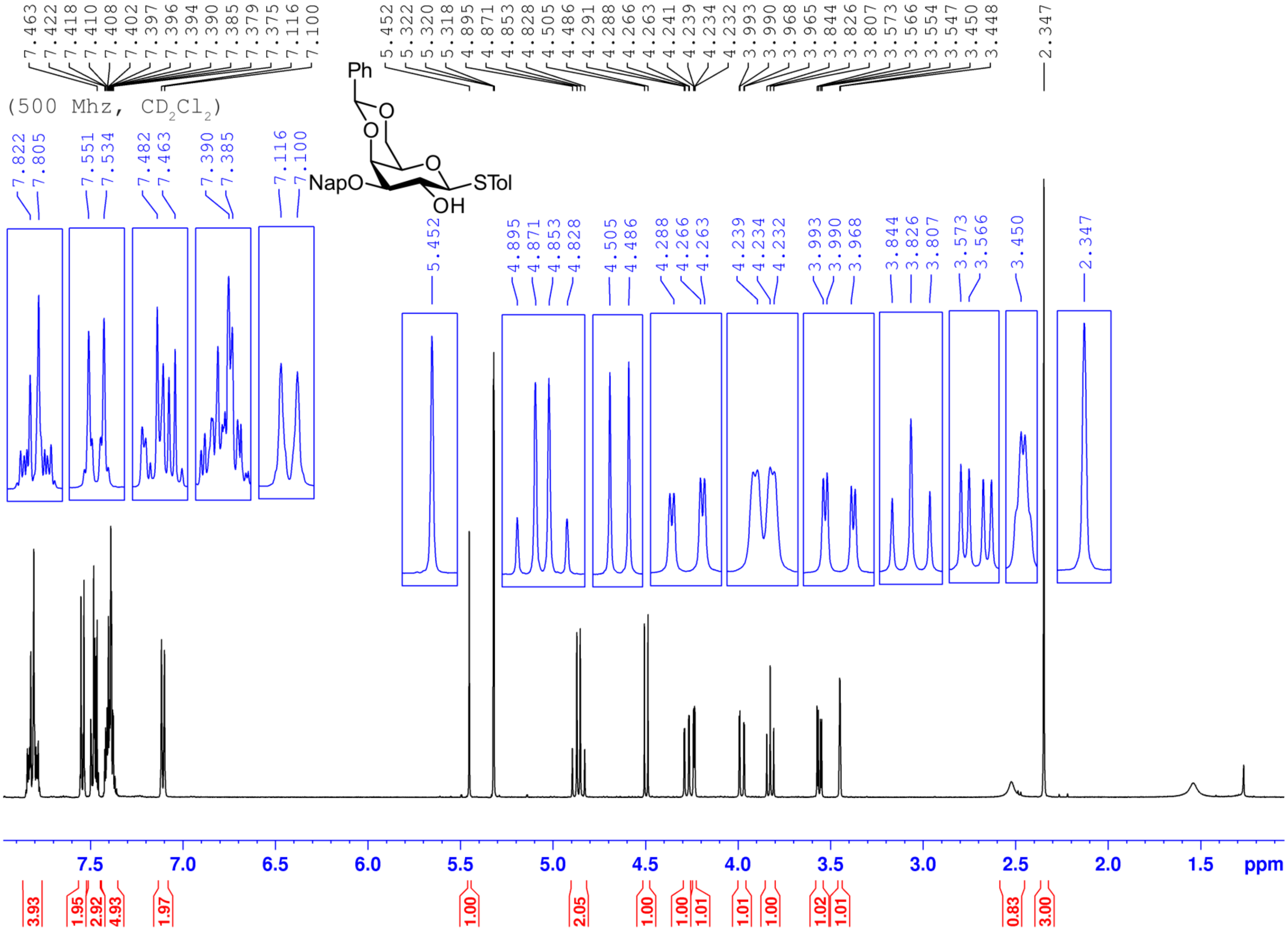

^{1}H-^{1}H COSY

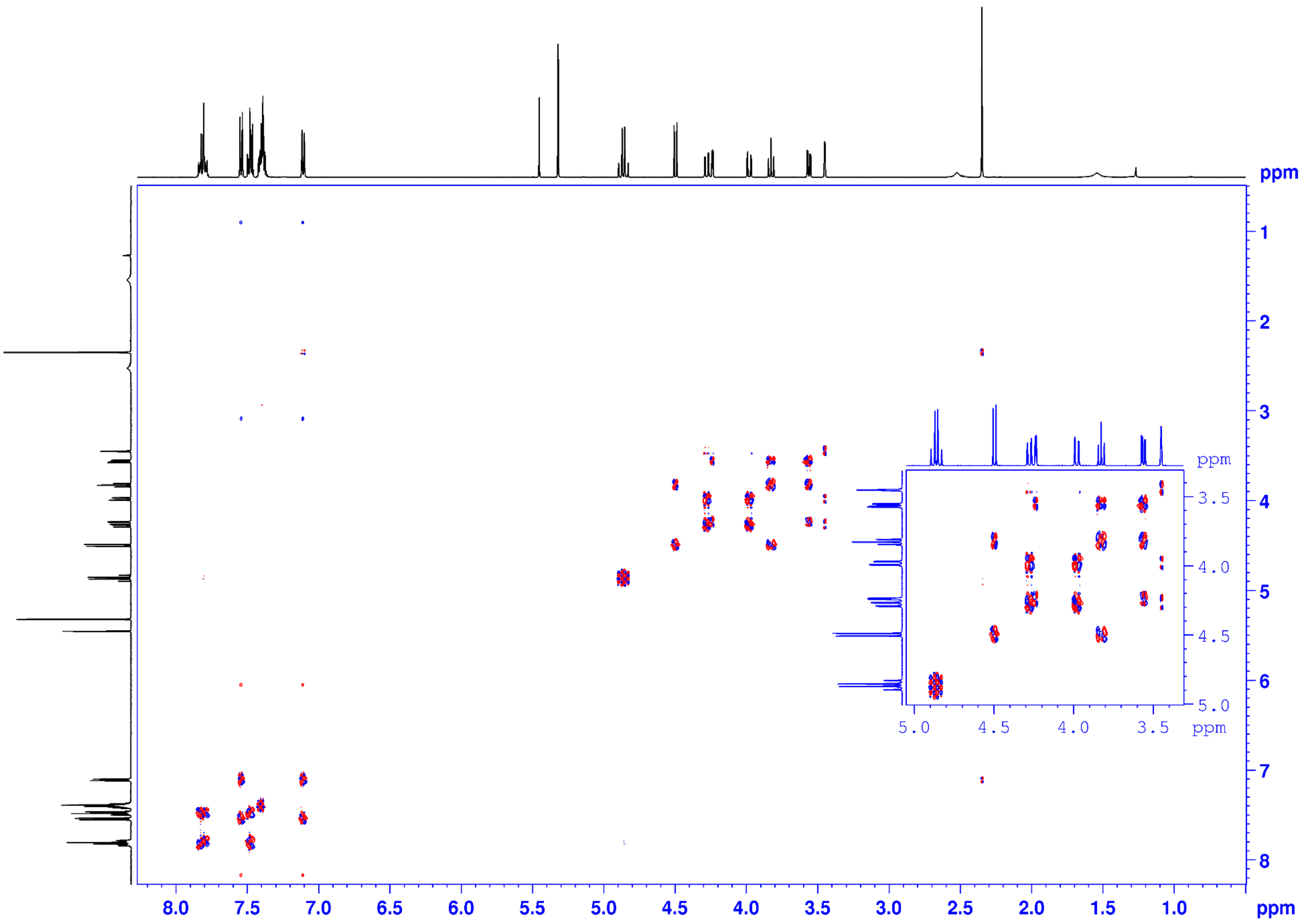

^{1}H-^{13}C HSQC

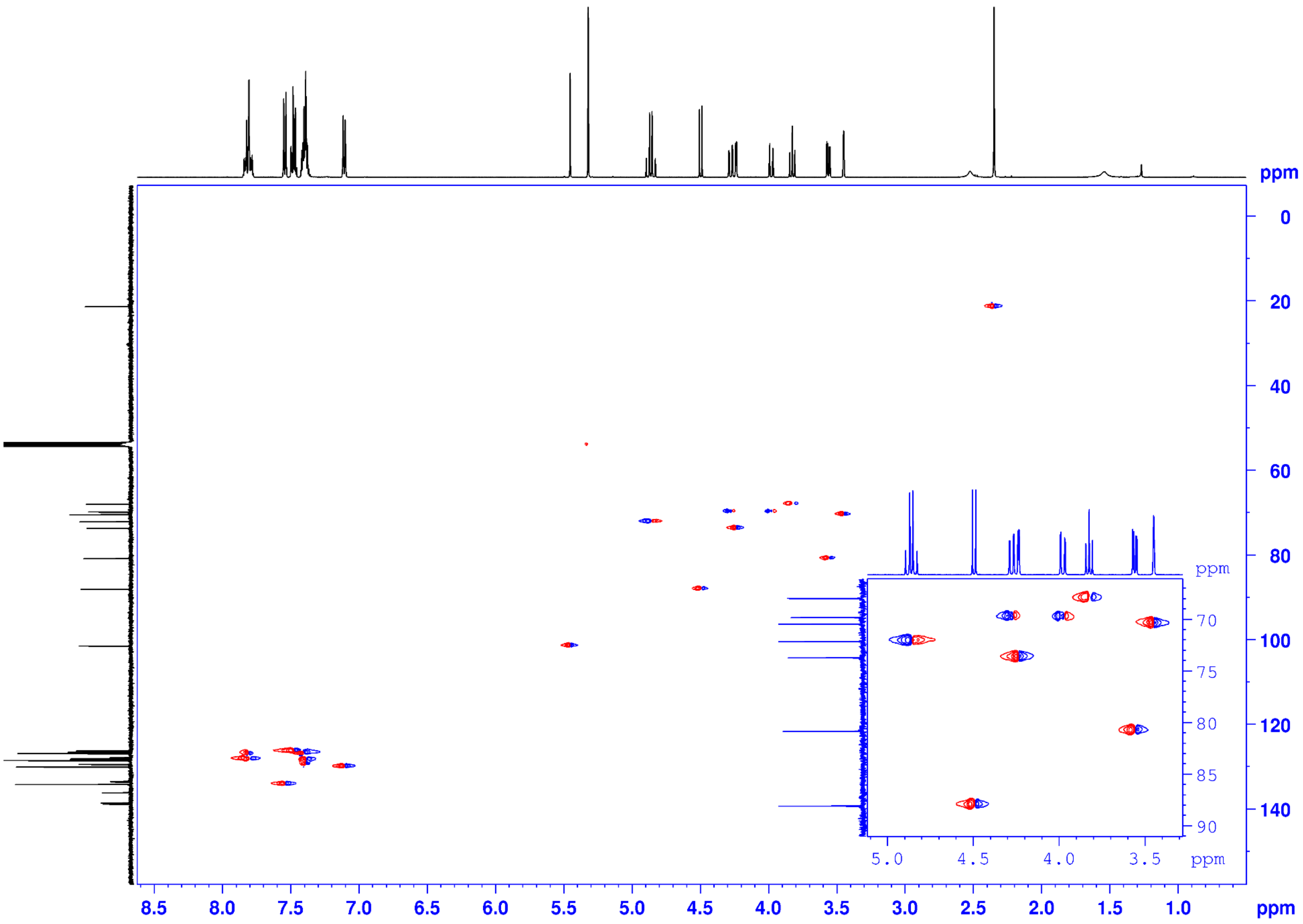

ppm
0
20
40
60
80
100
120
140
8.5
8.0
7.5
7.0
6.5
6.0
5.5
5.0
4.5
4.0
3.5
3.0
2.5
2.0
1.5
1.0
ppm
70
75
80
85
90
5.0
4.5
4.0
3.5

$^{13}C\{^1H\}$ NMR

(126 Mhz, CD_2Cl_2)

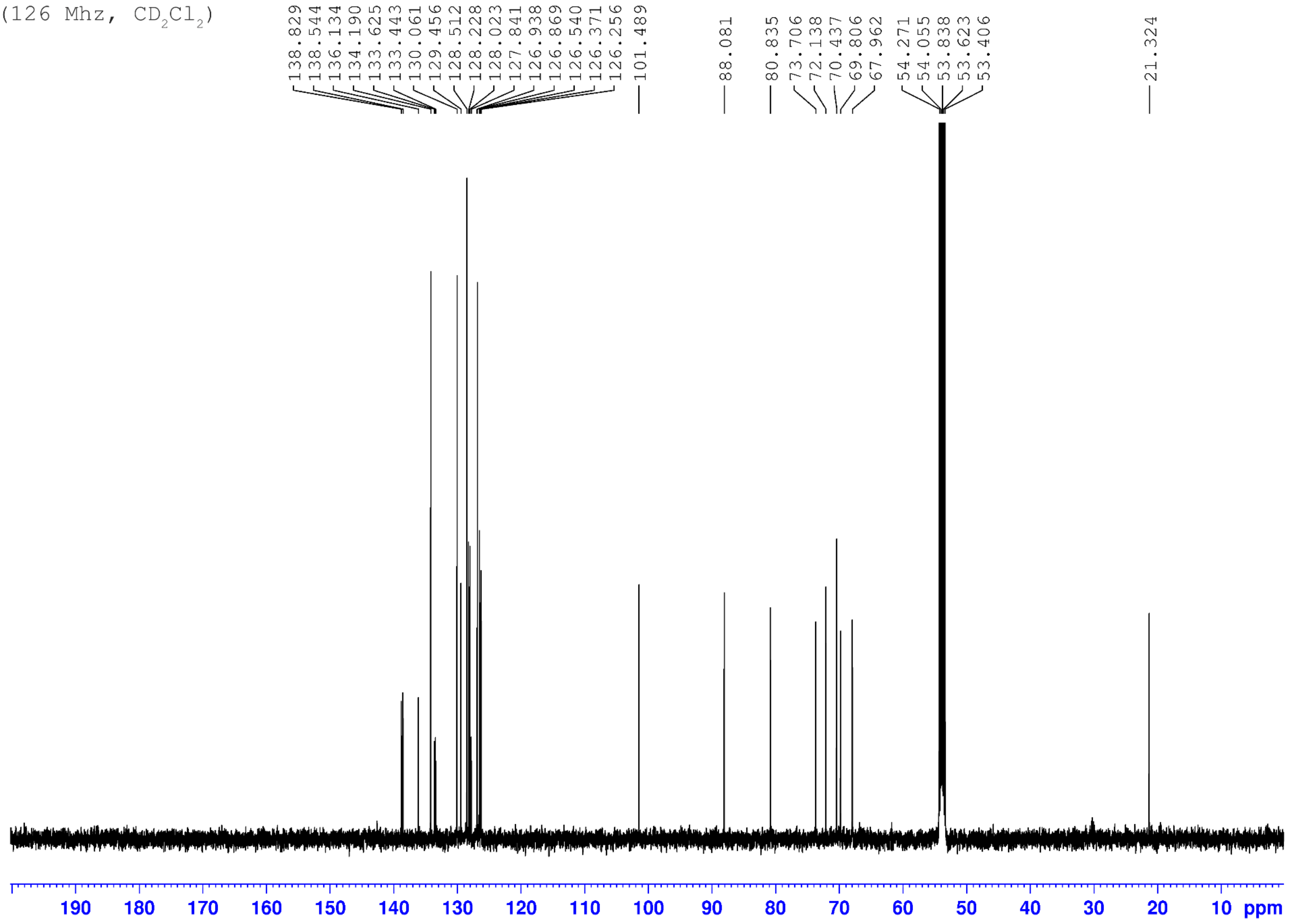

Compound **2b**

^{1}H-NMR

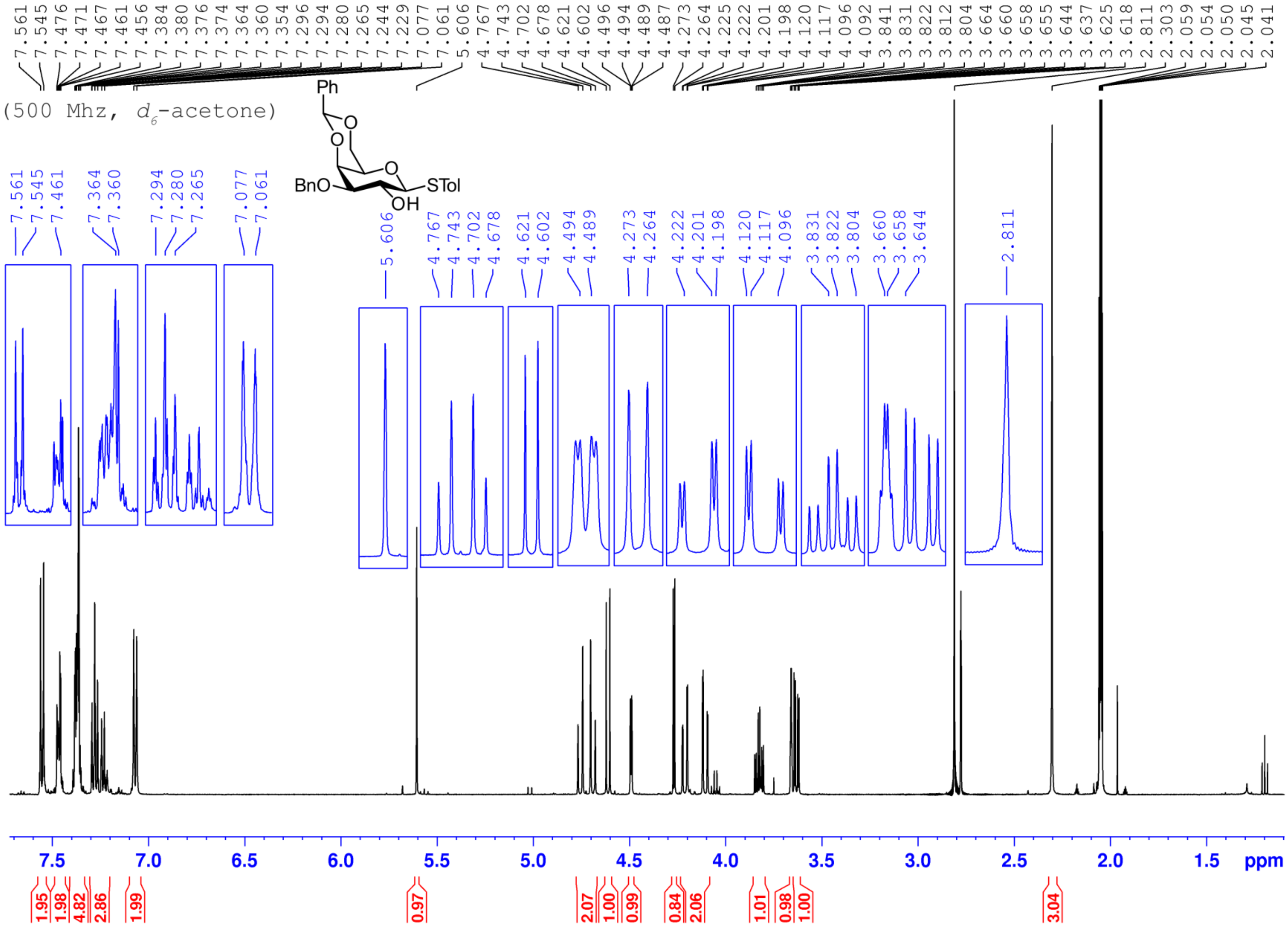

^{1}H-^{1}H COSY

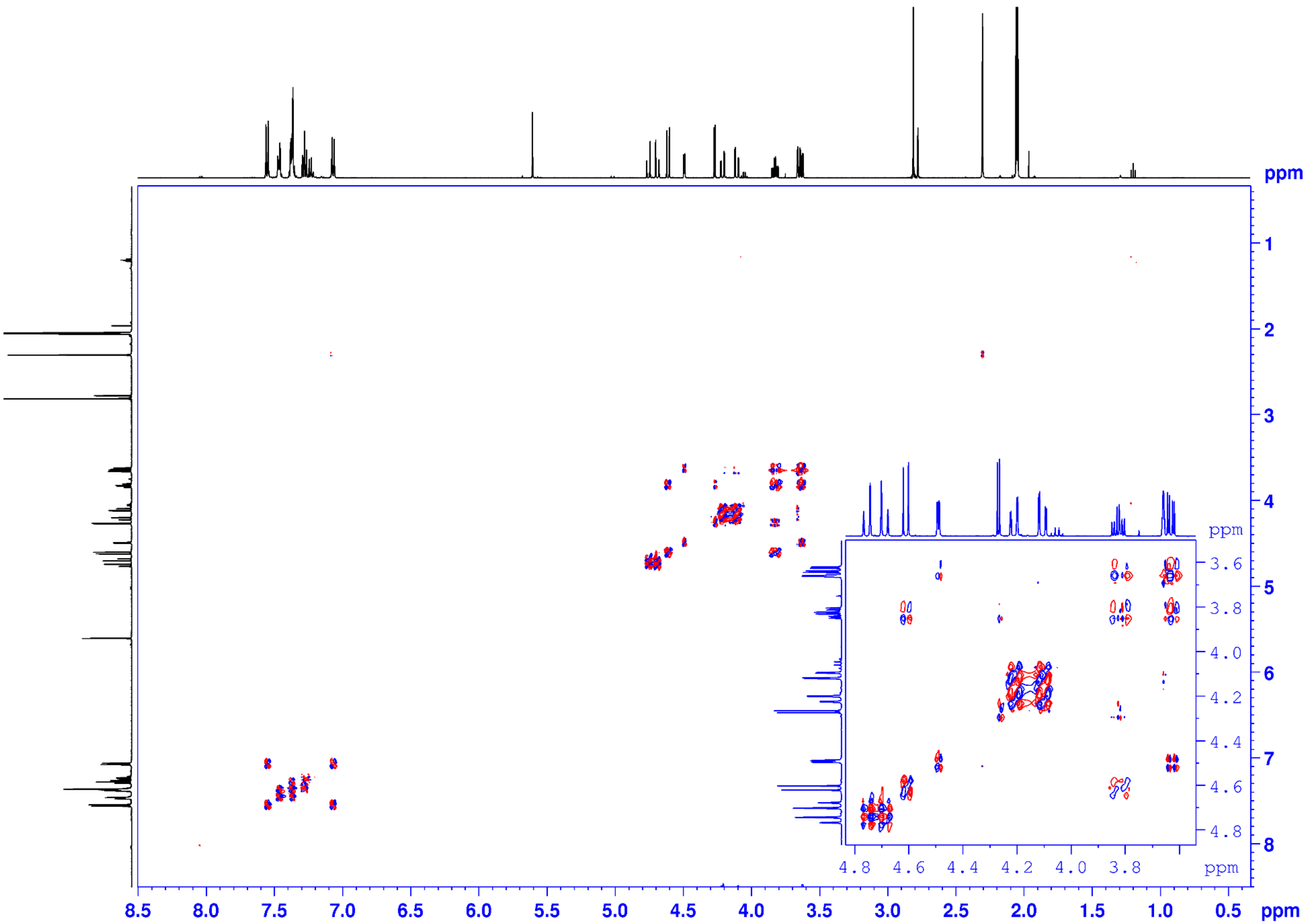

ppm
1
2
3
4
5
6
7
8
8.5
8.0
7.5
7.0
6.5
6.0
5.5
5.0
4.5
4.0
3.5
3.0
2.5
2.0
1.5
1.0
0.5
ppm
3.6
3.8
4.0
4.2
4.4
4.6
4.8
4.8
4.6
4.4
4.2
4.0
3.8
ppm

^{1}H-^{13}C HSQC

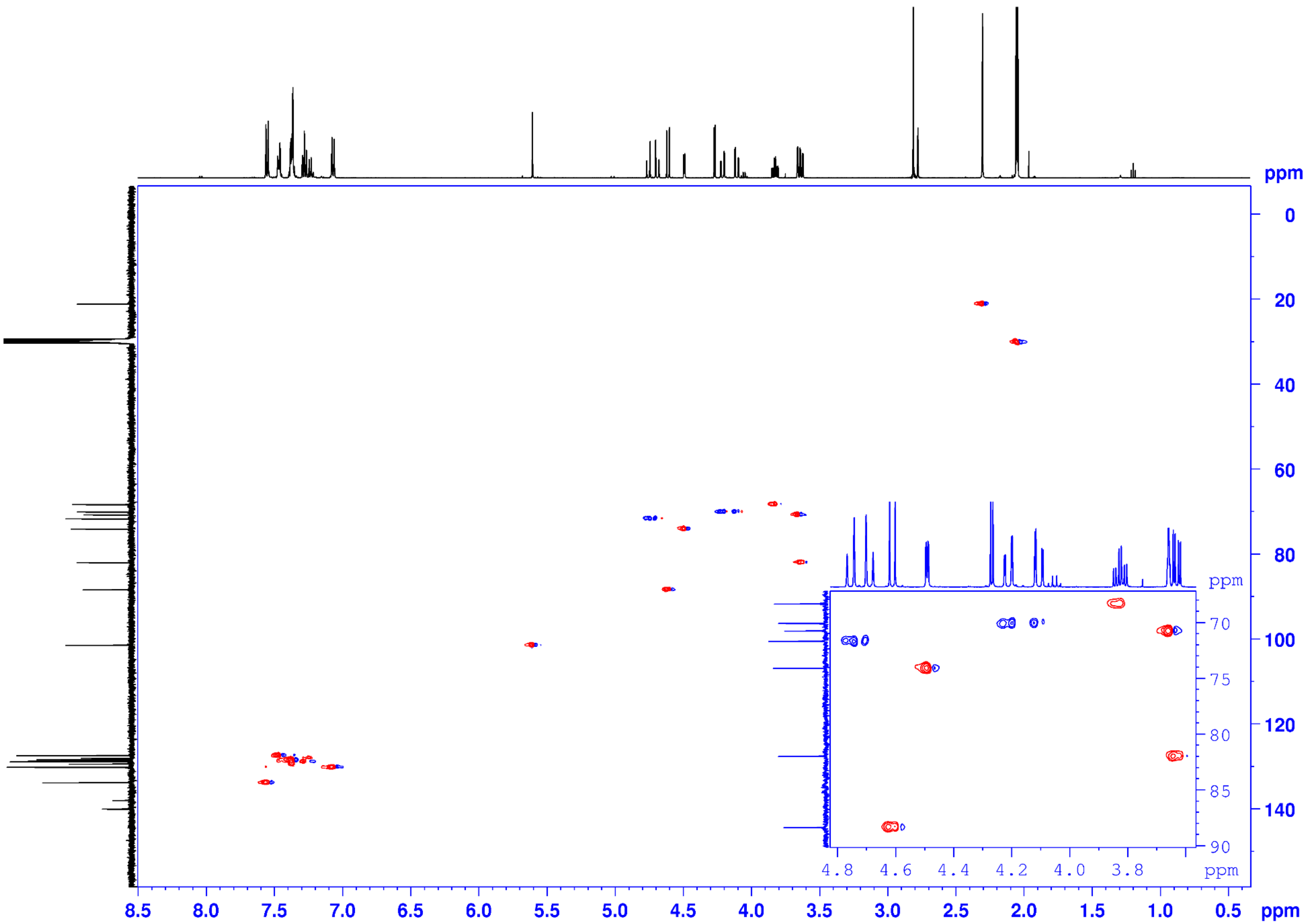

ppm
0
20
40
60
80
100
120
140
8.5
8.0
7.5
7.0
6.5
6.0
5.5
5.0
4.5
4.0
3.5
3.0
2.5
2.0
1.5
1.0
0.5
ppm
70
75
80
85
90
4.8
4.6
4.4
4.2
4.0
3.8
ppm

$^{13}C\{^{1}H\}$ NMR

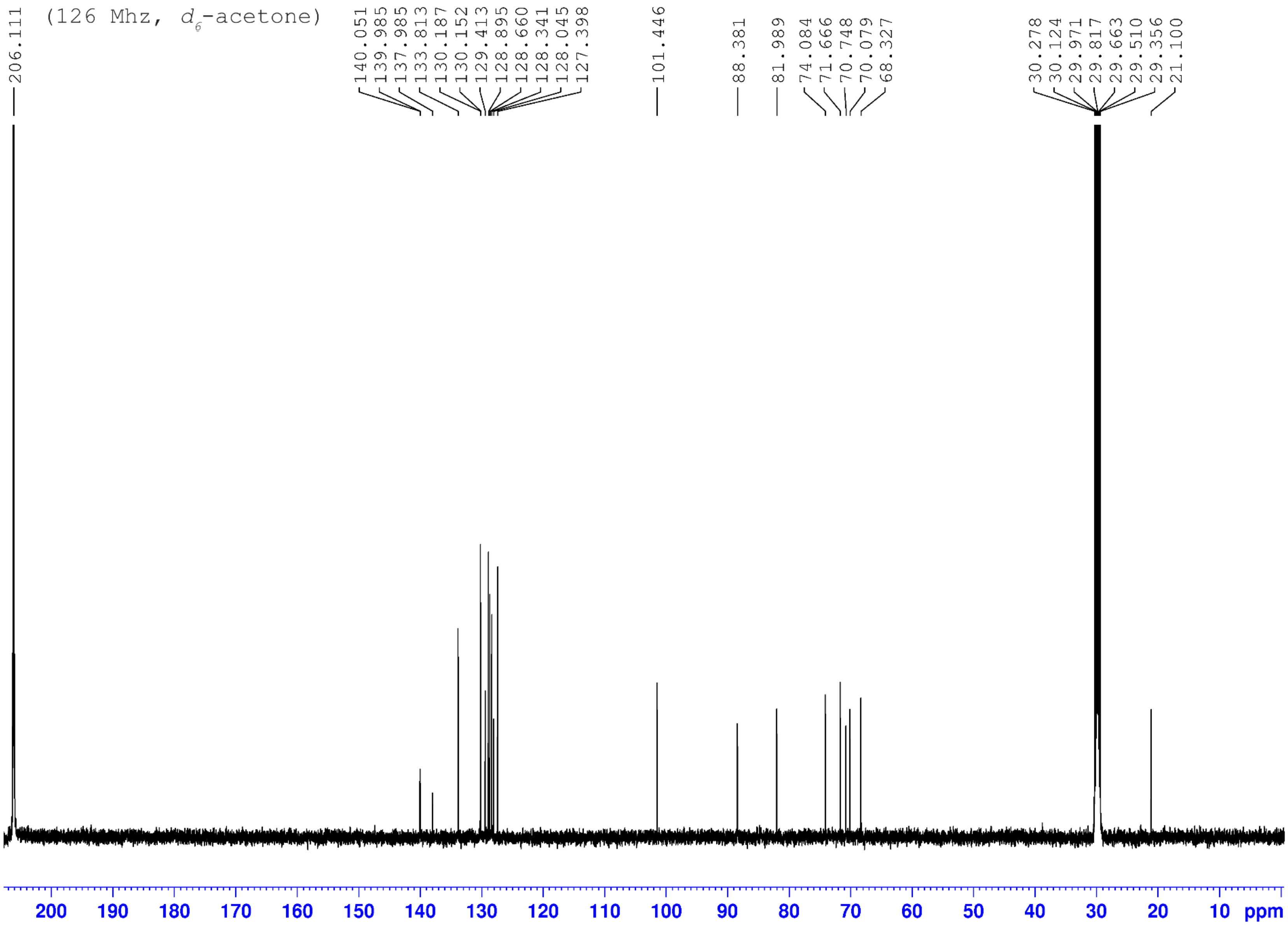
(126 Mhz, d6-acetone)
206.111
140.051
139.985
137.985
133.813
130.187
130.152
129.413
128.895
128.660
128.341
128.045
127.398
101.446
88.381
81.989
74.084
71.666
70.748
70.079
68.327
30.278
30.124
29.971
29.817
29.663
29.510
29.356
21.100
200 190 180 170 160 150 140 130 120 110 100 90 80 70 60 50 40 30 20 10 ppm

Compound **2c**

^{1}H-NMR

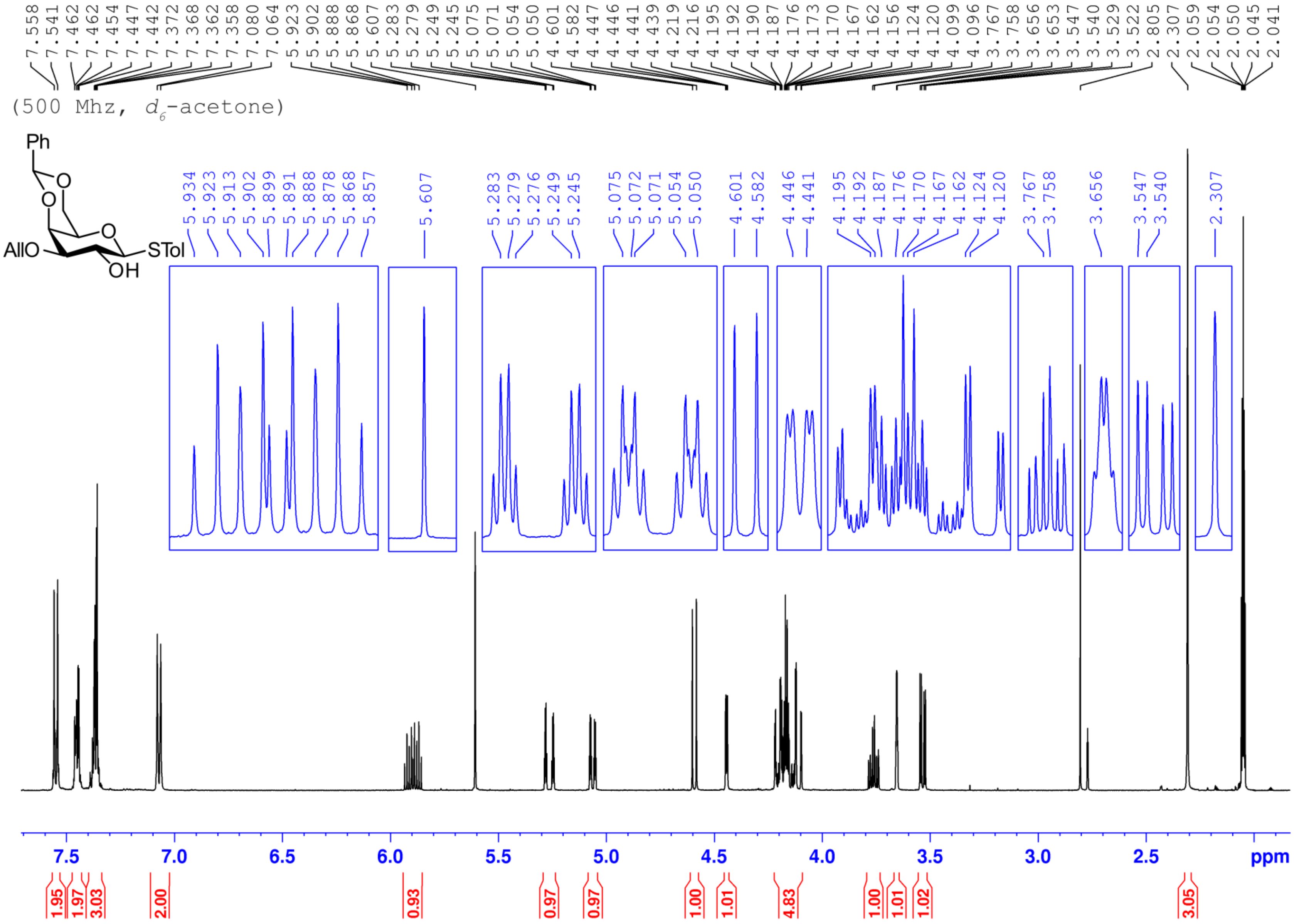

^{1}H-^{1}H COSY

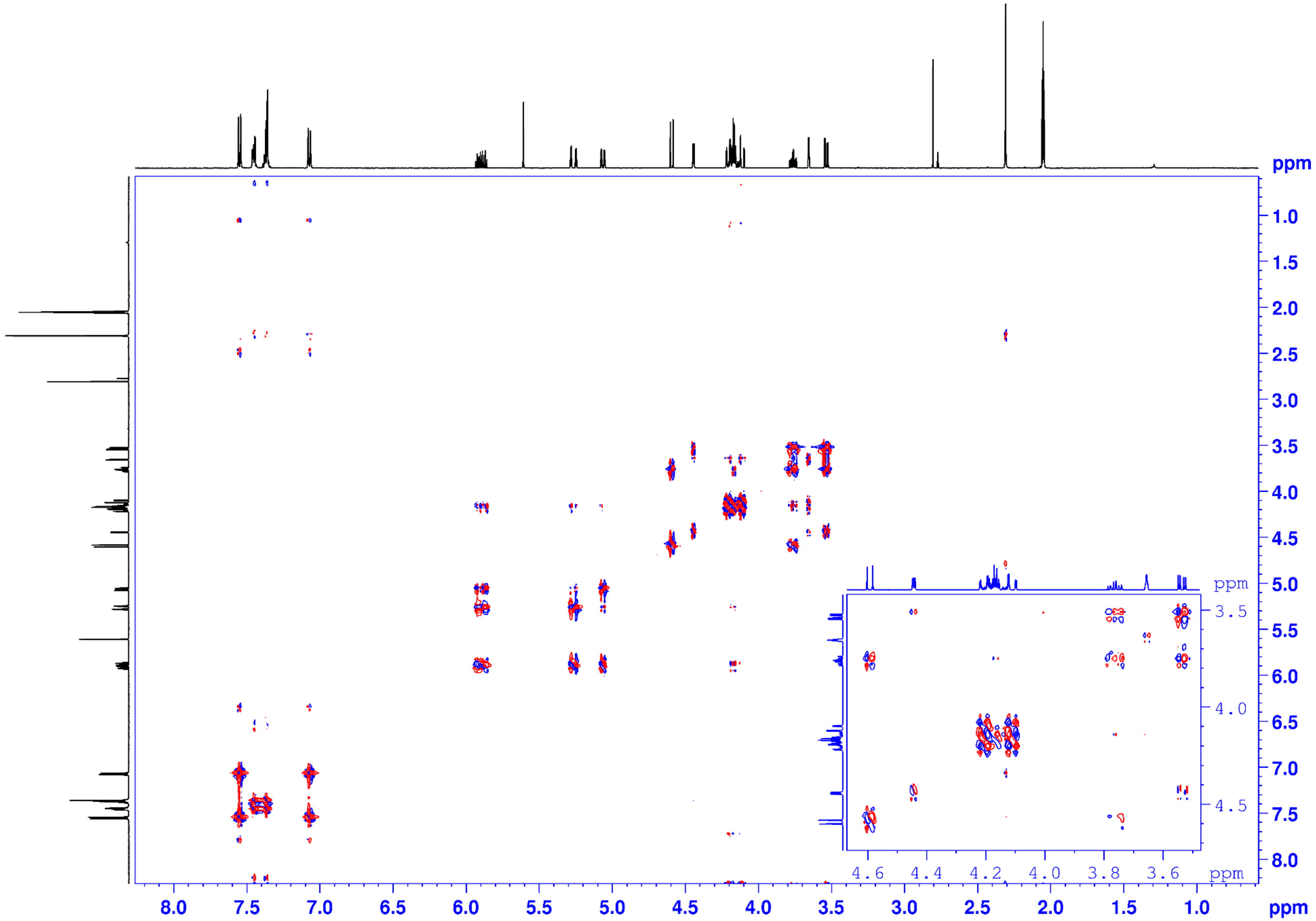
ppm
1.0
1.5
2.0
2.5
3.0
3.5
4.0
4.5
5.0
5.5
6.0
6.5
7.0
7.5
8.0
8.0 7.5 7.0 6.5 6.0 5.5 5.0 4.5 4.0 3.5 3.0 2.5 2.0 1.5 1.0
ppm
3.5
4.0
4.5
4.6 4.4 4.2 4.0 3.8 3.6

^{1}H-^{13}C HSQC

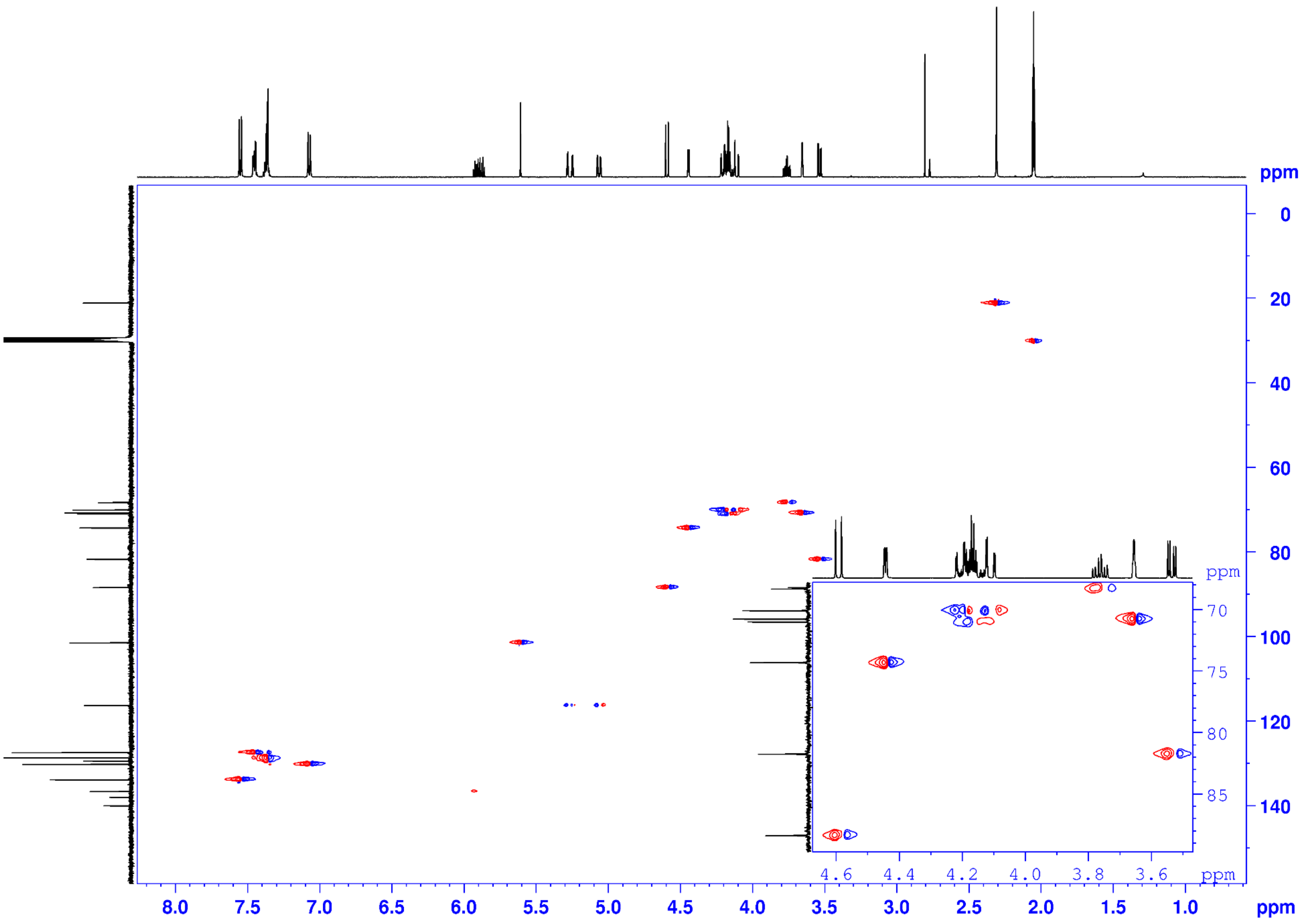

ppm
0
20
40
60
80
100
120
140
8.0
7.5
7.0
6.5
6.0
5.5
5.0
4.5
4.0
3.5
3.0
2.5
2.0
1.5
1.0
ppm
70
75
80
85
4.6
4.4
4.2
4.0
3.8
3.6

$^{13}C\{^{1}H\}$ NMR

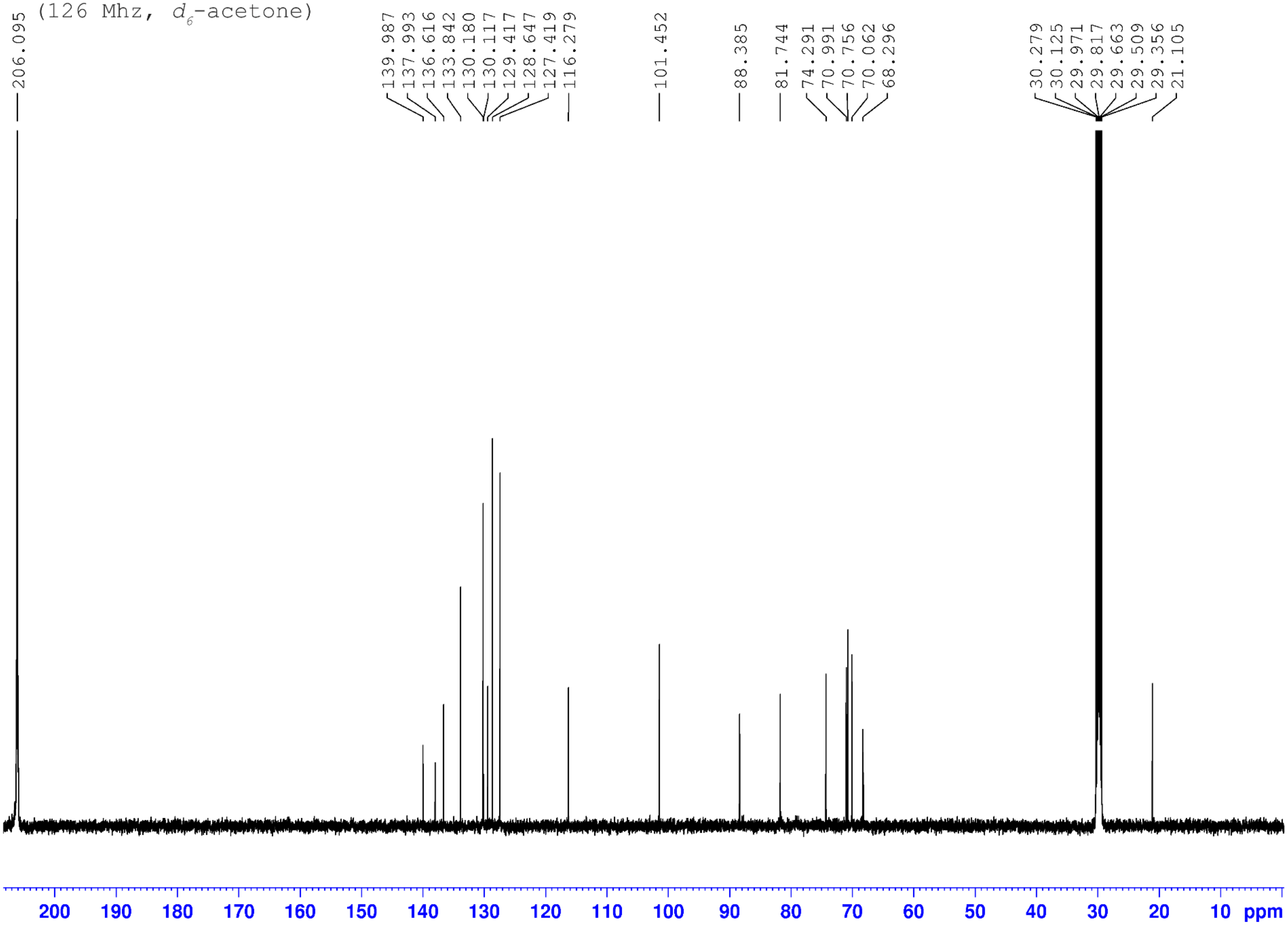
(126 Mhz, d_6-acetone)
206.095
139.987
137.993
136.616
133.842
130.180
130.117
129.417
128.647
127.419
116.279
101.452
88.385
81.744
74.291
70.991
70.756
70.062
68.296
30.279
30.125
29.971
29.817
29.663
29.509
29.356
21.105
200
190
180
170
160
150
140
130
120
110
100
90
80
70
60
50
40
30
20
10
ppm

Compound **2d**

^{1}H-NMR

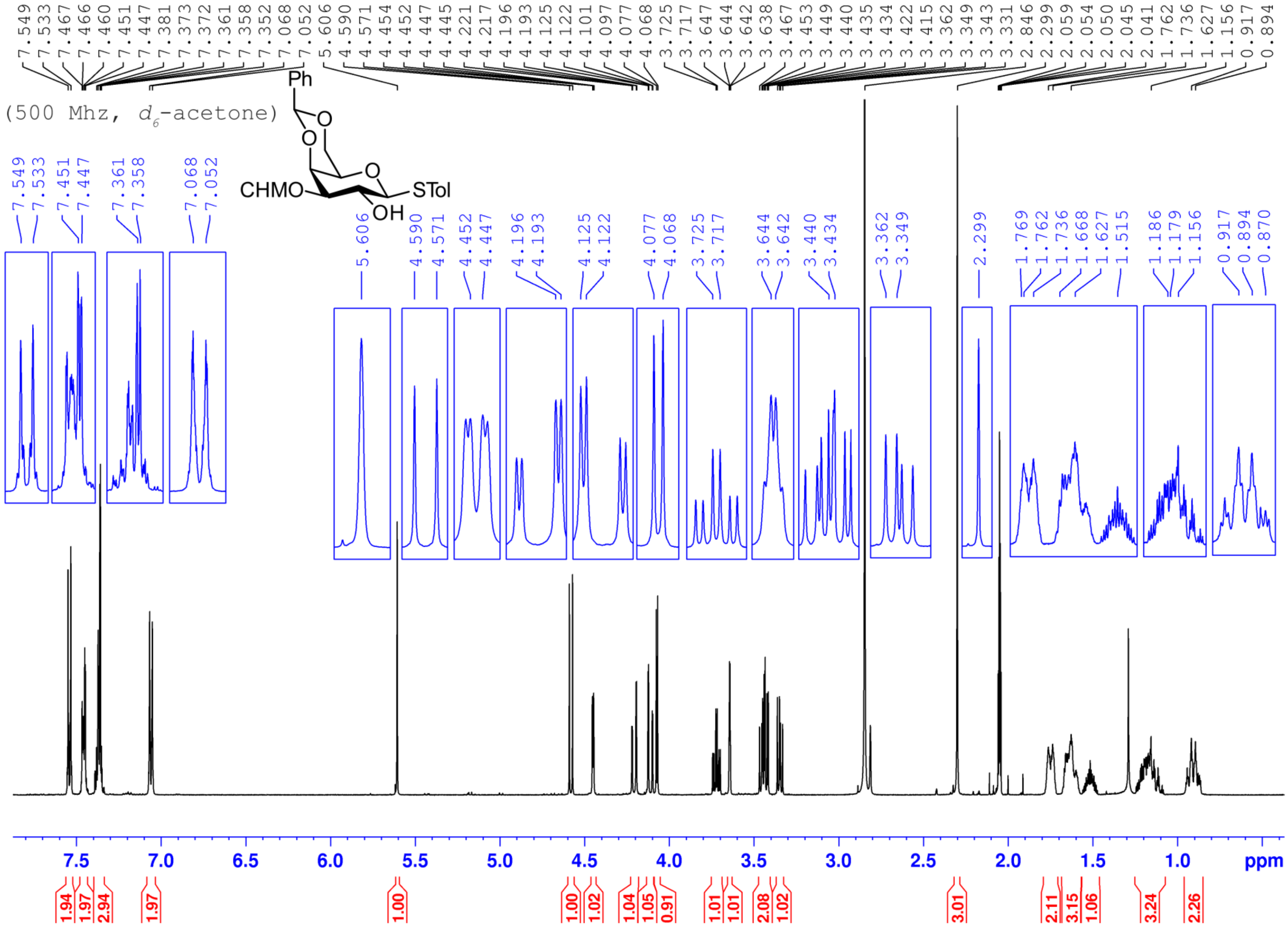

^{1}H-^{1}H COSY

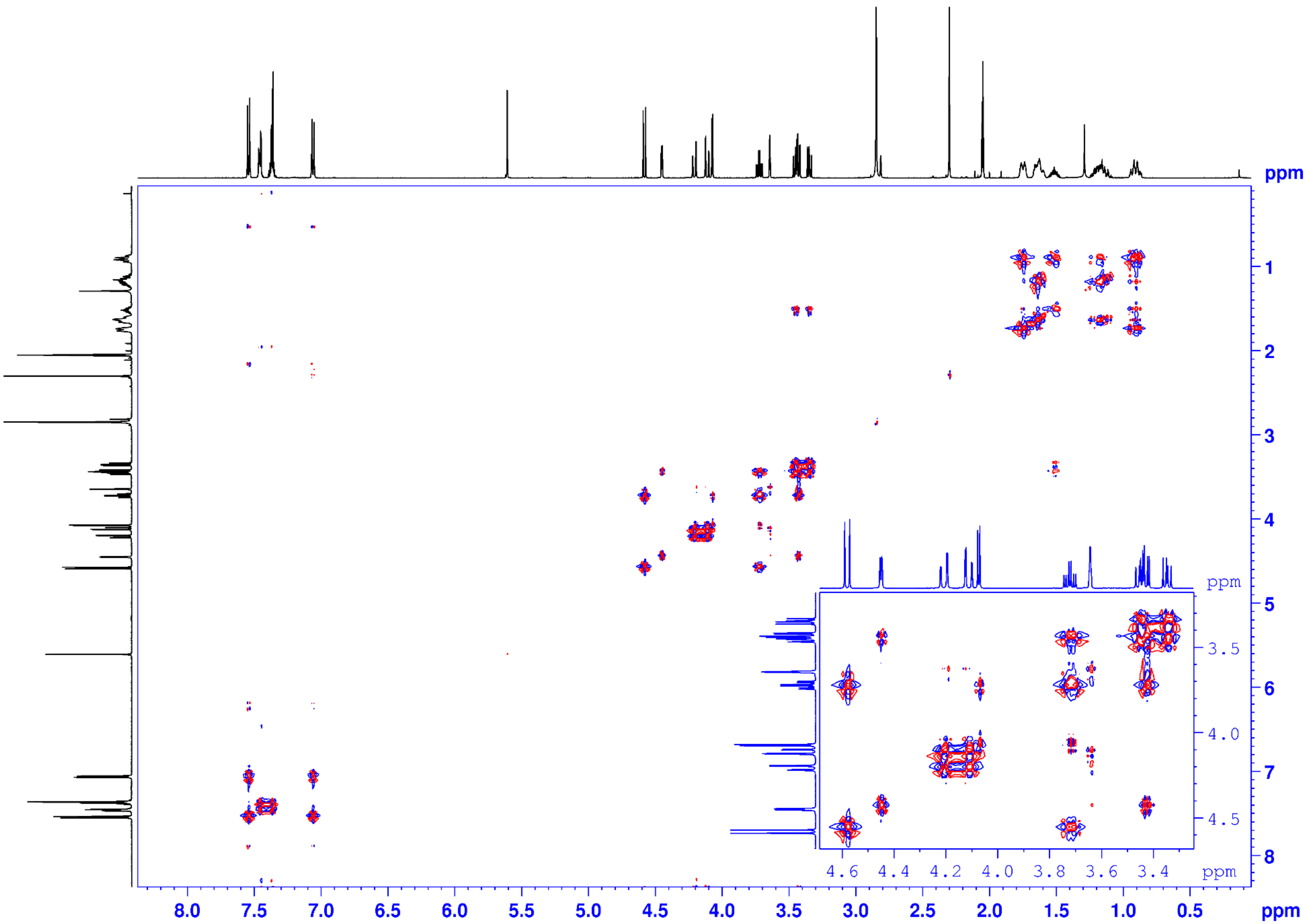

ppm
8.0
7.5
7.0
6.5
6.0
5.5
5.0
4.5
4.0
3.5
3.0
2.5
2.0
1.5
1.0
0.5
ppm
1
2
3
4
5
6
7
8
4.6
4.4
4.2
4.0
3.8
3.6
3.4
ppm
3.5
4.0
4.5
ppm

^{1}H-^{13}C HSQC

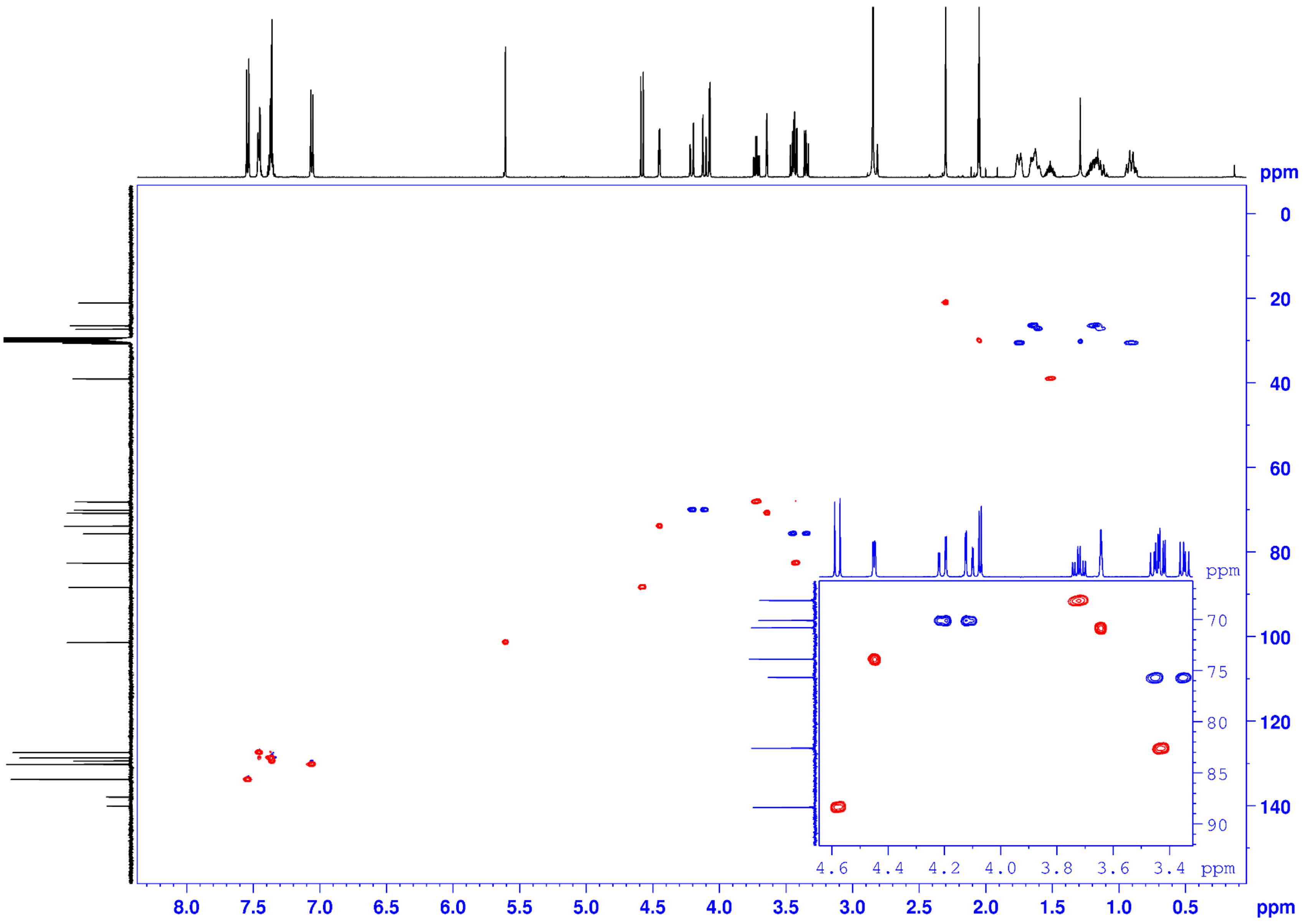

ppm
0
20
40
60
80
100
120
140
8.0
7.5
7.0
6.5
6.0
5.5
5.0
4.5
4.0
3.5
3.0
2.5
2.0
1.5
1.0
0.5
ppm
4.6
4.4
4.2
4.0
3.8
3.6
3.4
ppm
70
75
80
85
90

$^{13}C\{^1H\}$ NMR

(126 Mhz, d_6-acetone)

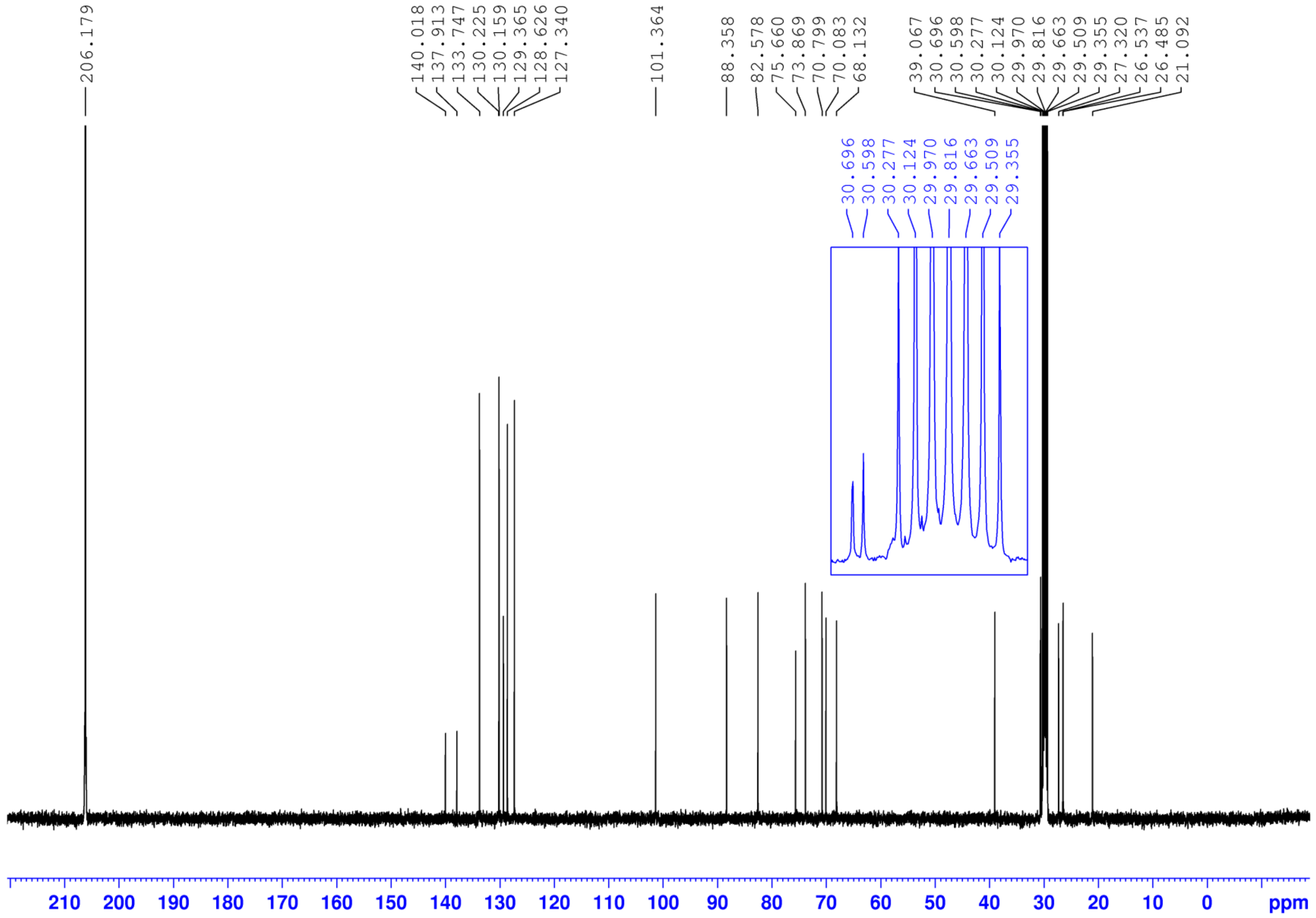

Compound **2e**

^{1}H-NMR

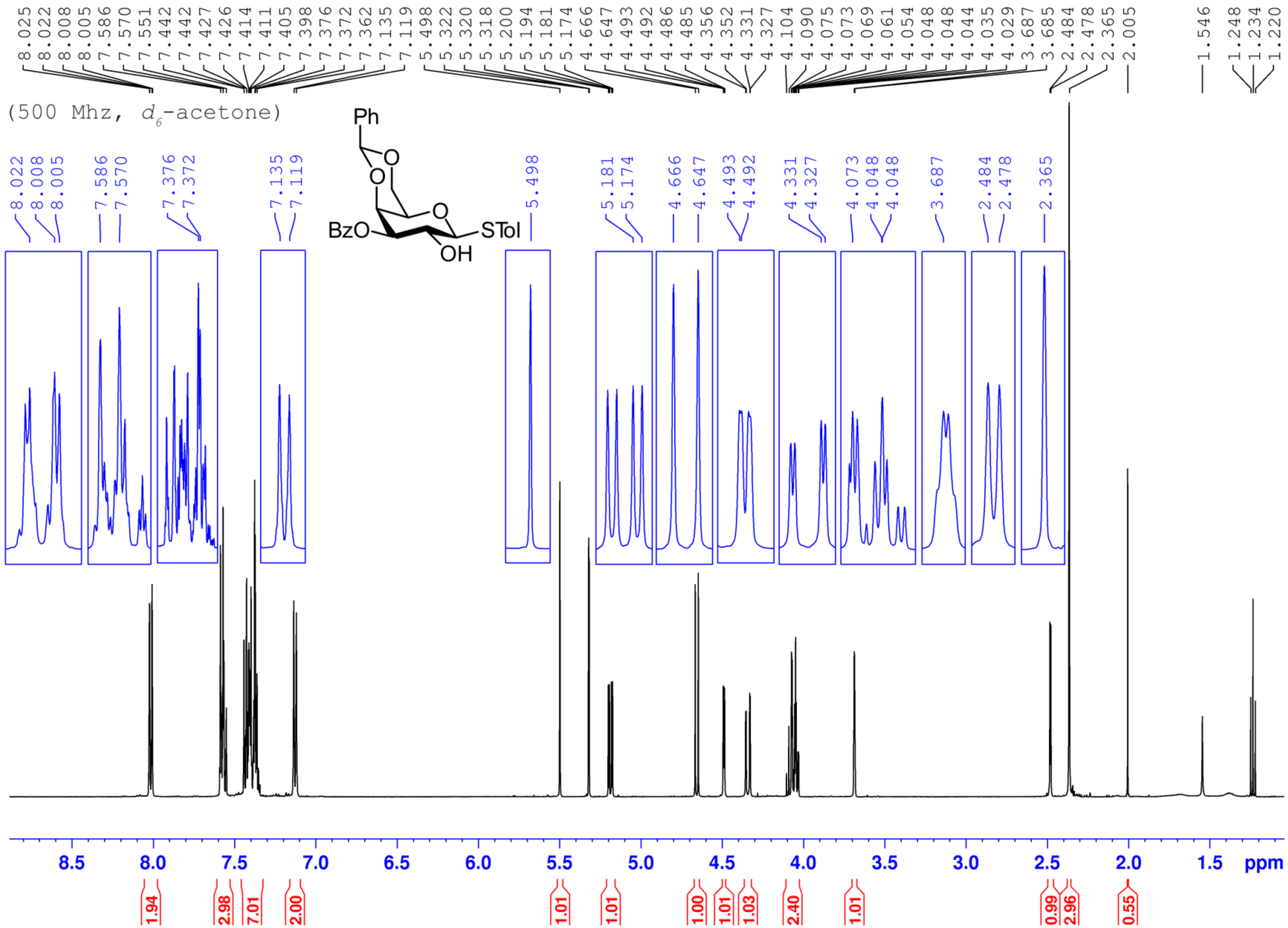

^{1}H-^{1}H COSY

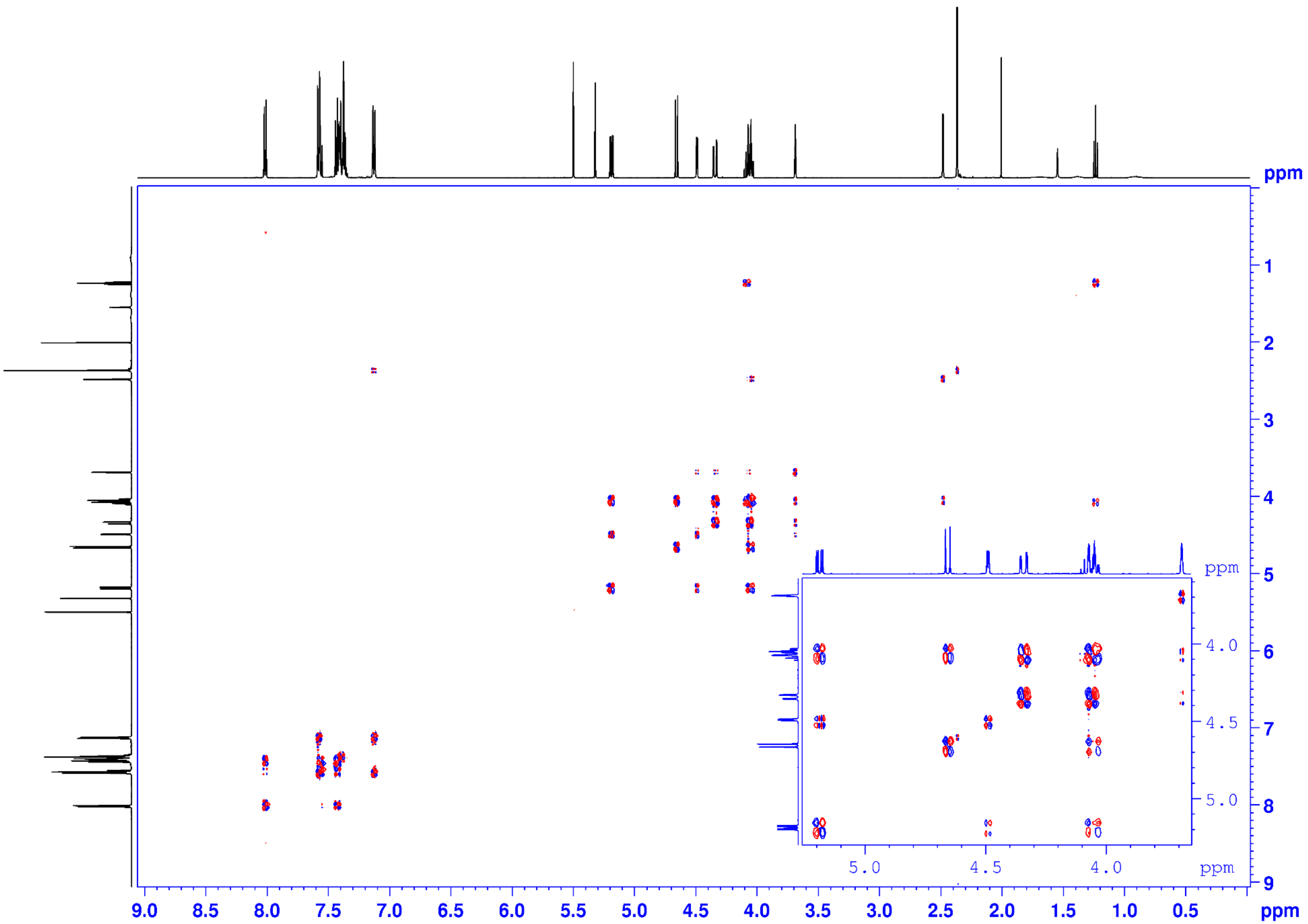
ppm
1
2
3
4
5
6
7
8
9
9.0
8.5
8.0
7.5
7.0
6.5
6.0
5.5
5.0
4.5
4.0
3.5
3.0
2.5
2.0
1.5
1.0
0.5
ppm
5.0
4.5
4.0
ppm

^{1}H-^{13}C HSQC

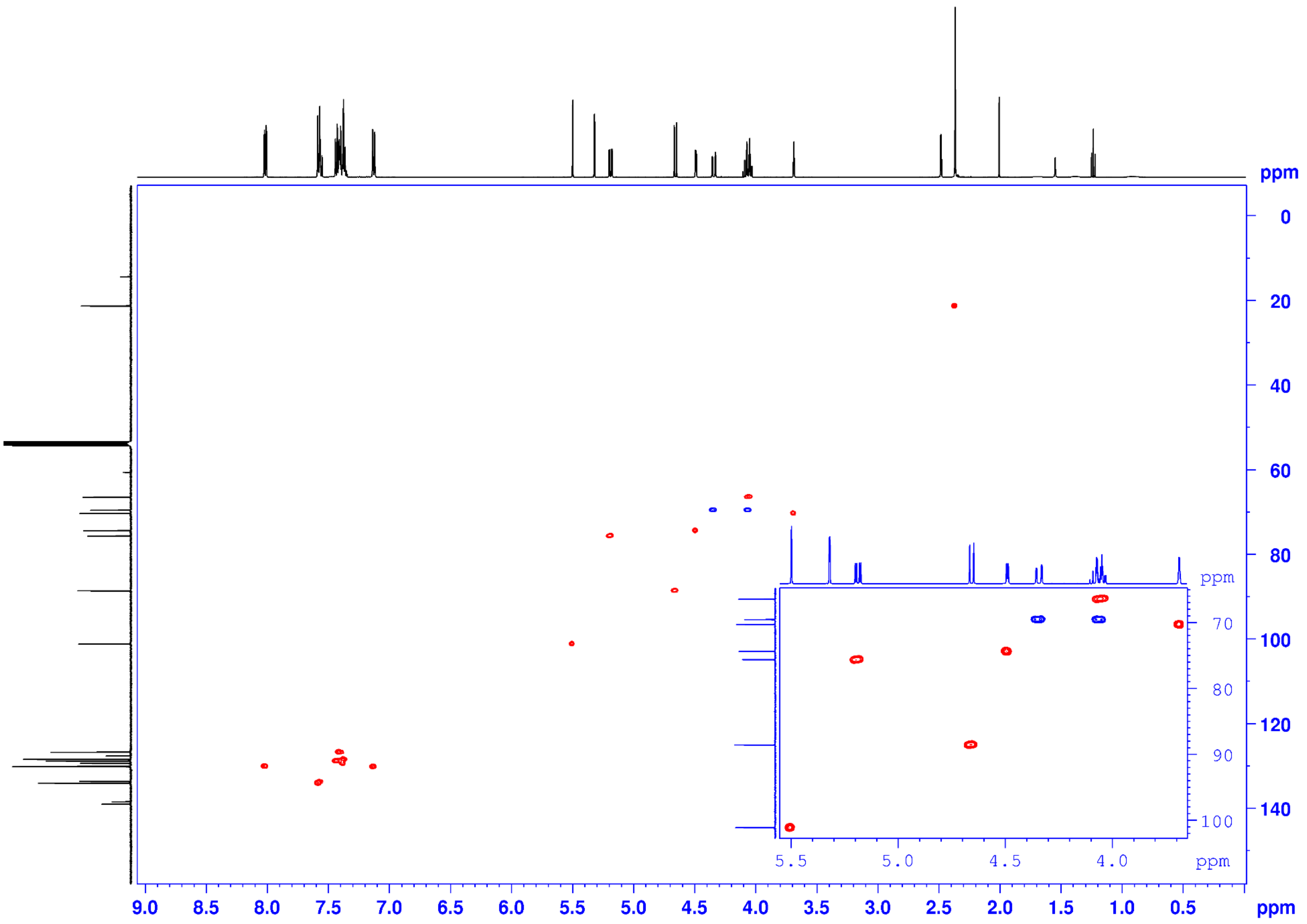
ppm
0
20
40
60
80
100
120
140
9.0 8.5 8.0 7.5 7.0 6.5 6.0 5.5 5.0 4.5 4.0 3.5 3.0 2.5 2.0 1.5 1.0 0.5
ppm
70
80
90
100
5.5 5.0 4.5 4.0
ppm

$^{13}C\{^1H\}$ NMR
(126 Mhz, d_6-acetone)

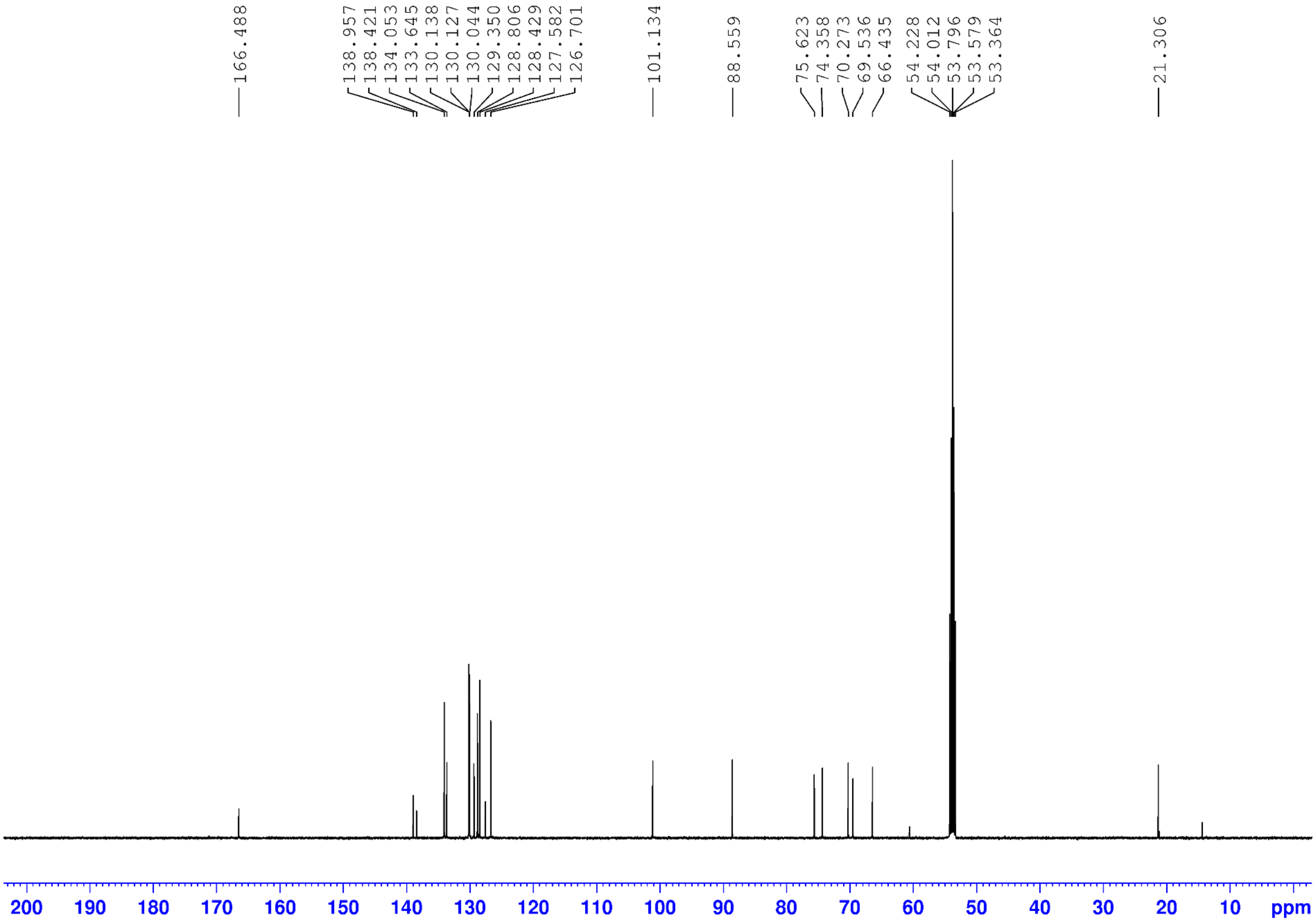

Compound **3a**

^{1}H-NMR

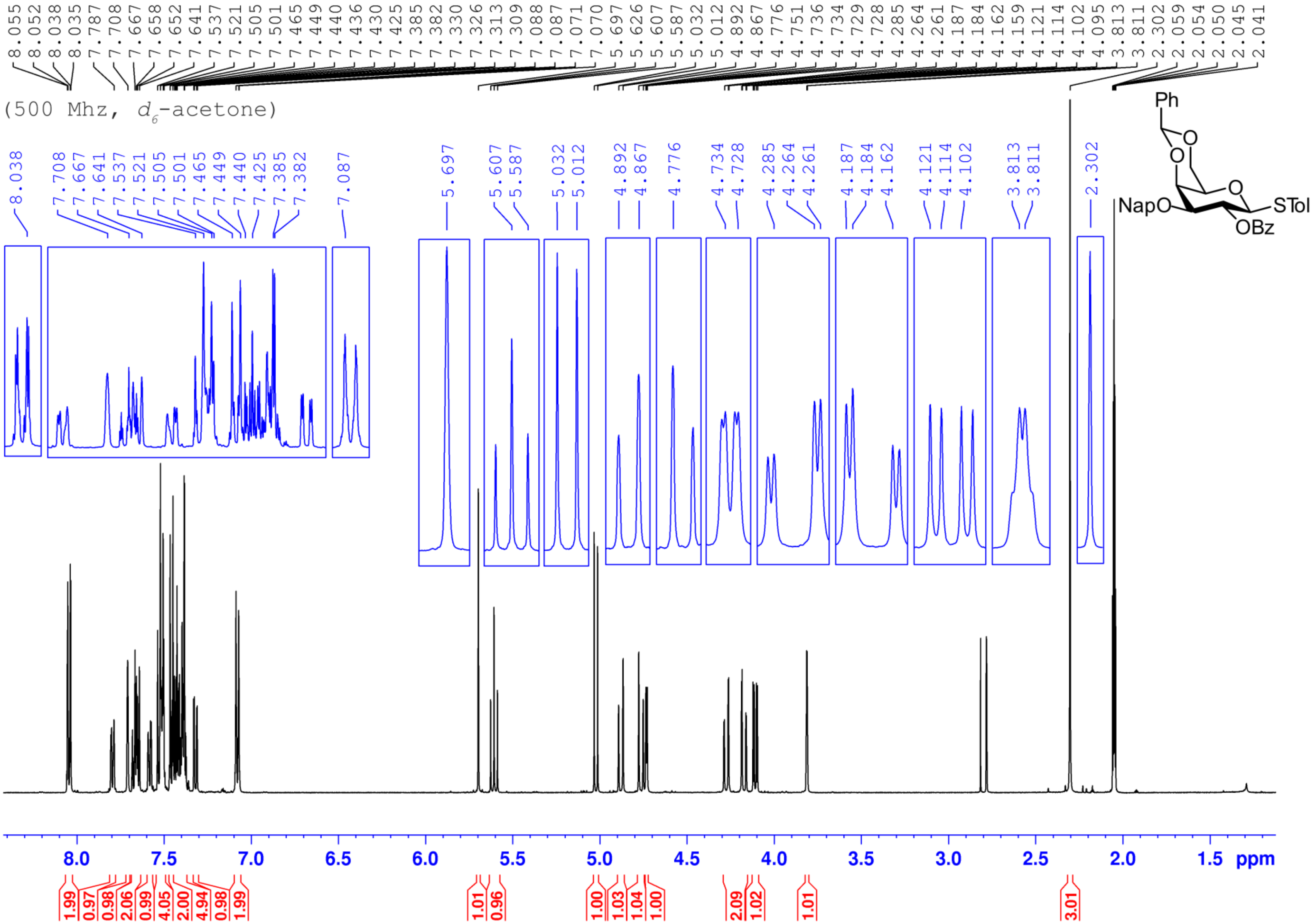

^1H-^1H COSY

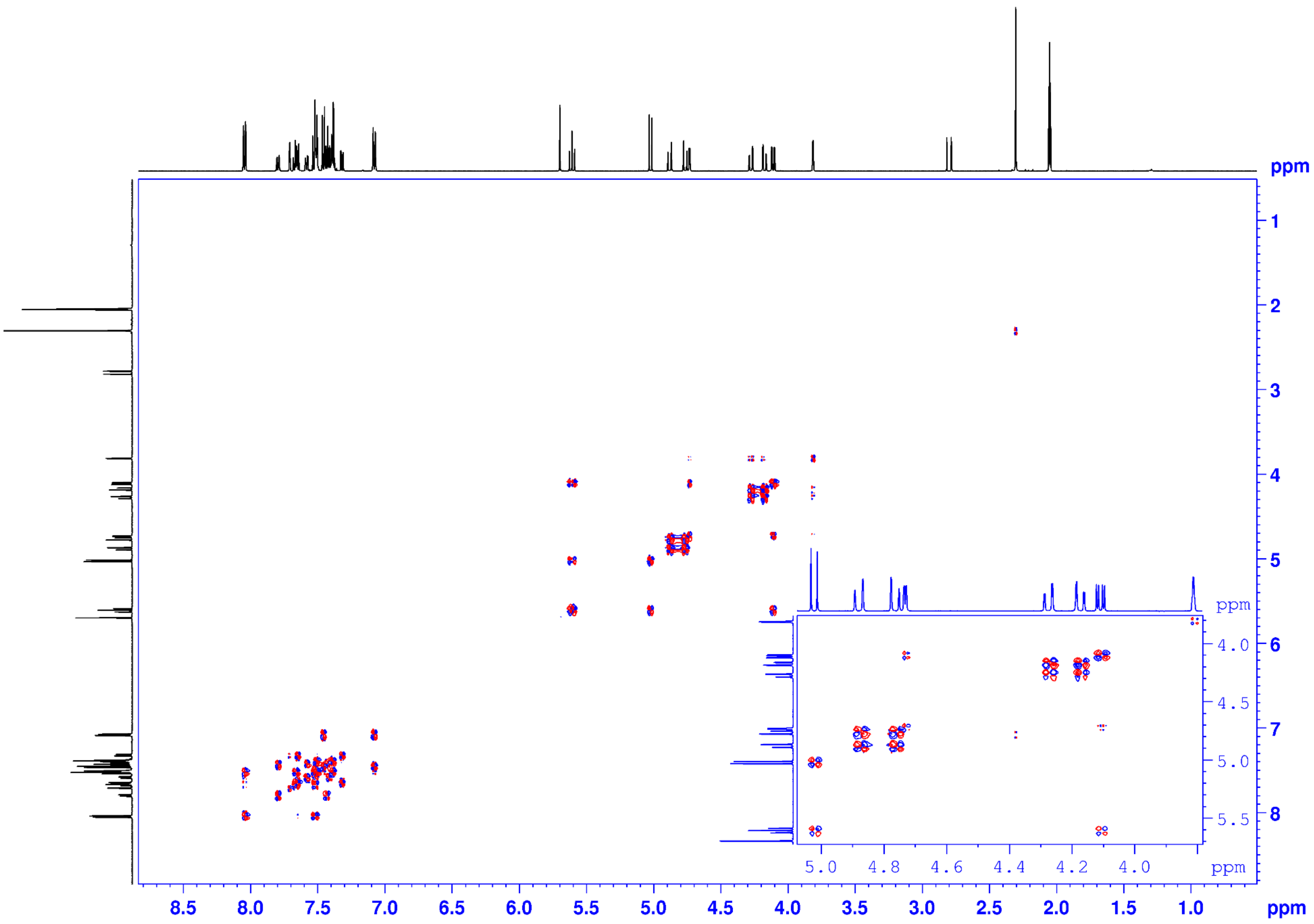
ppm
1
2
3
4
5
6
7
8
8.5
8.0
7.5
7.0
6.5
6.0
5.5
5.0
4.5
4.0
3.5
3.0
2.5
2.0
1.5
1.0
ppm
4.0
4.5
5.0
5.5
5.0
4.8
4.6
4.4
4.2
4.0
ppm

^{1}H-^{13}C HSQC

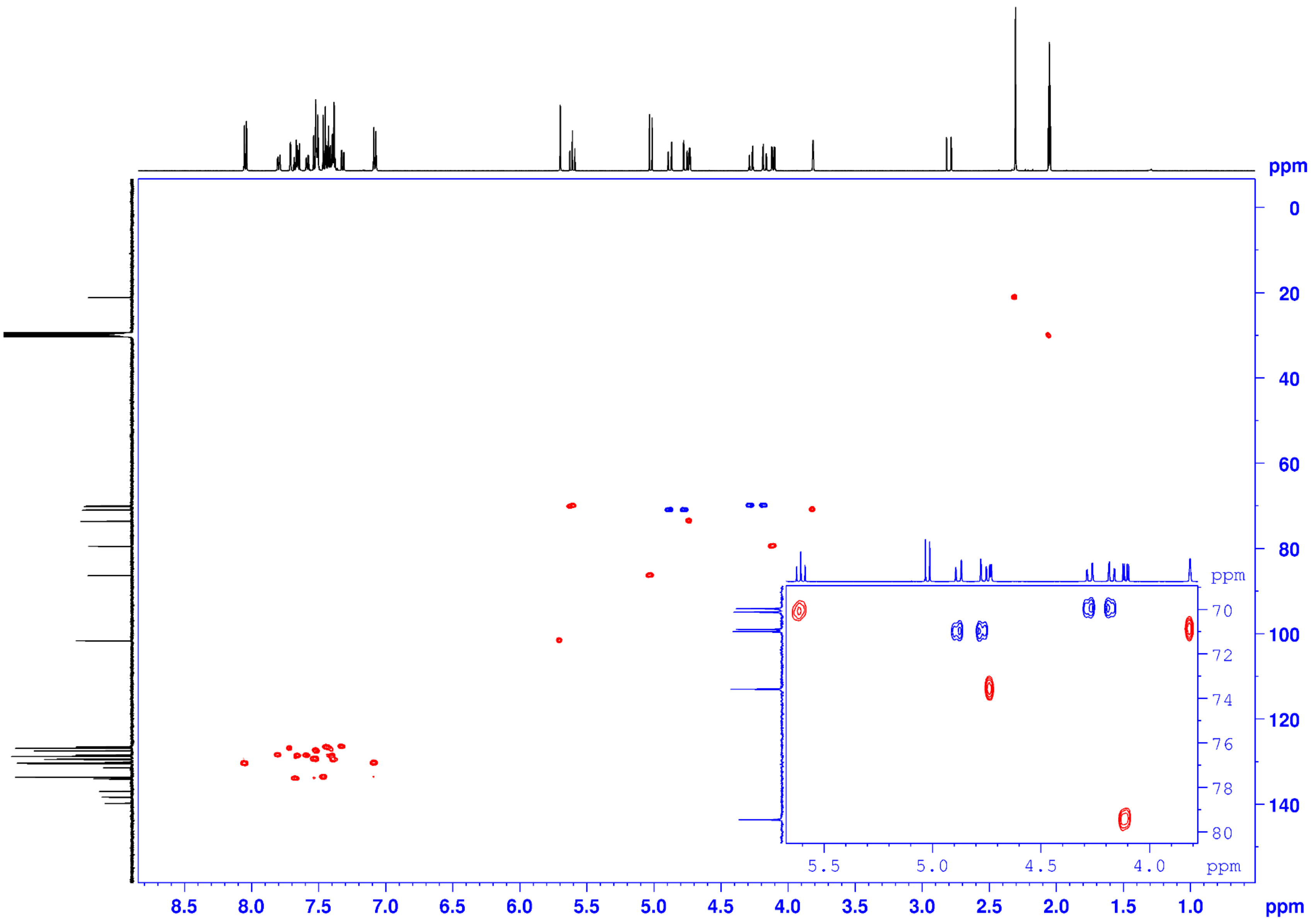

ppm
0
20
40
60
80
100
120
140
8.5
8.0
7.5
7.0
6.5
6.0
5.5
5.0
4.5
4.0
3.5
3.0
2.5
2.0
1.5
1.0
ppm
70
72
74
76
78
80
5.5
5.0
4.5
4.0

^{1}H-^{13}C HMBC

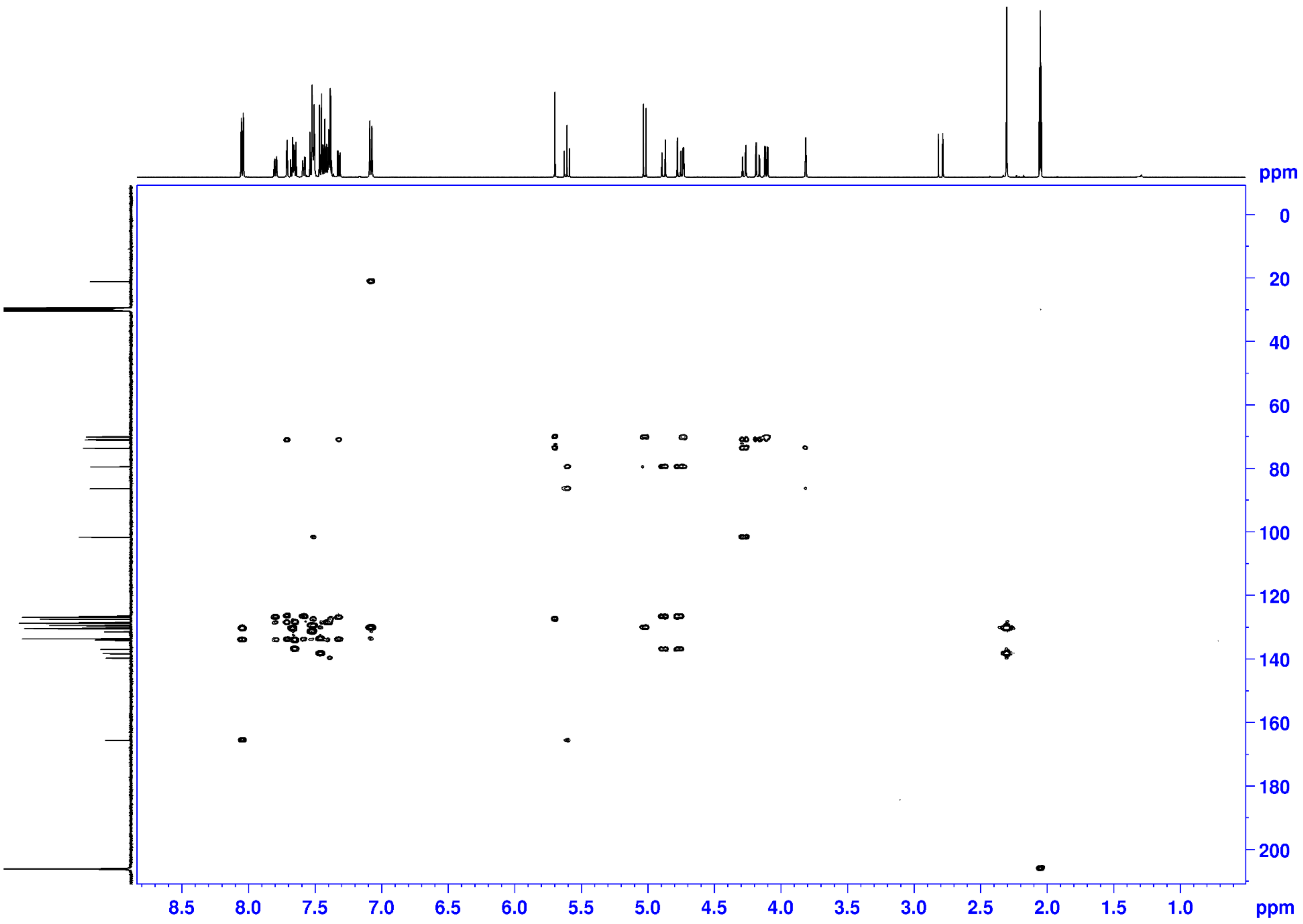
ppm
0
20
40
60
80
100
120
140
160
180
200
8.5 8.0 7.5 7.0 6.5 6.0 5.5 5.0 4.5 4.0 3.5 3.0 2.5 2.0 1.5 1.0 ppm

$^{13}C\{^1H\}$ NMR

(126 Mhz, d_6-acetone)

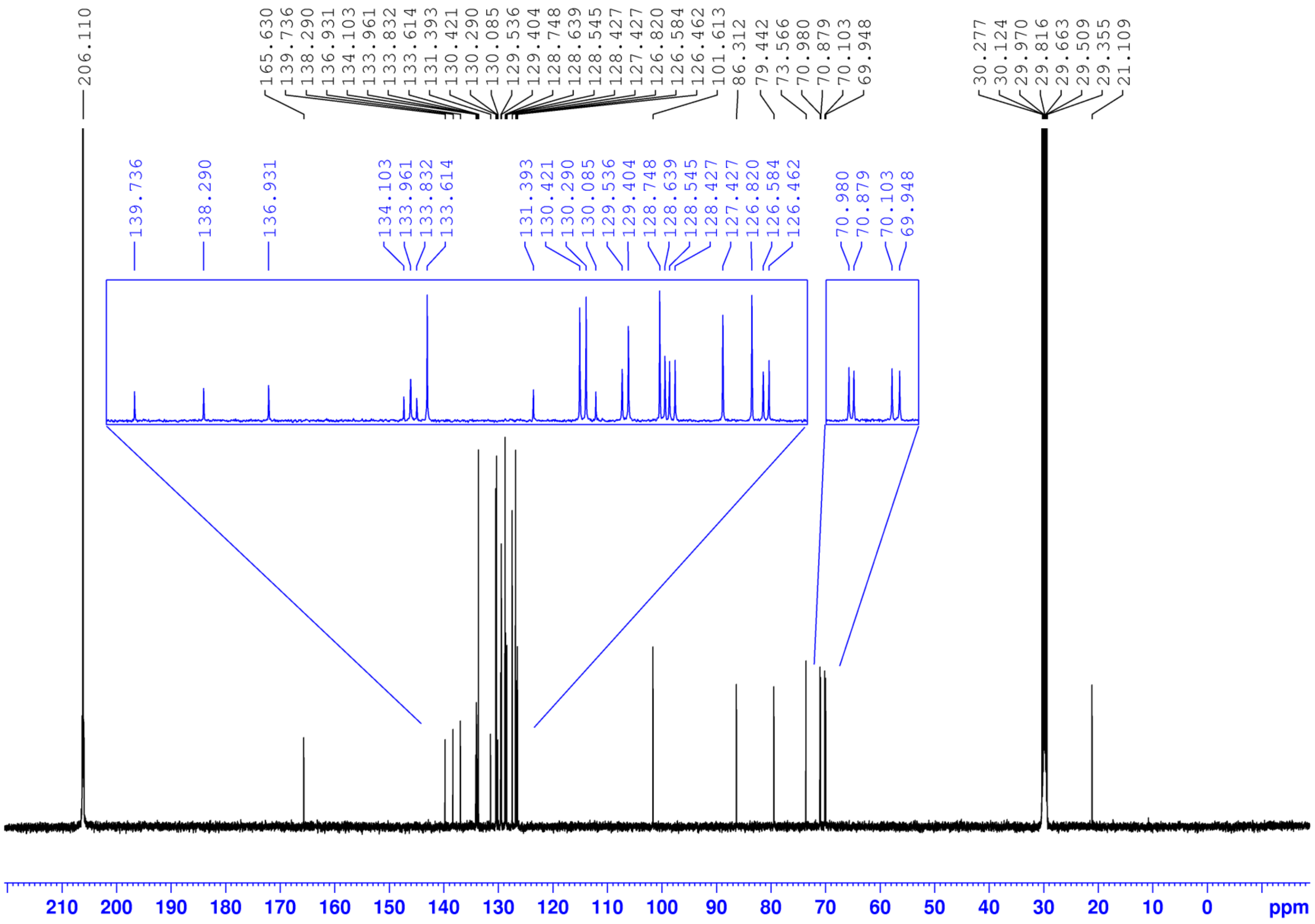

Compound **3c**

^{1}H-NMR

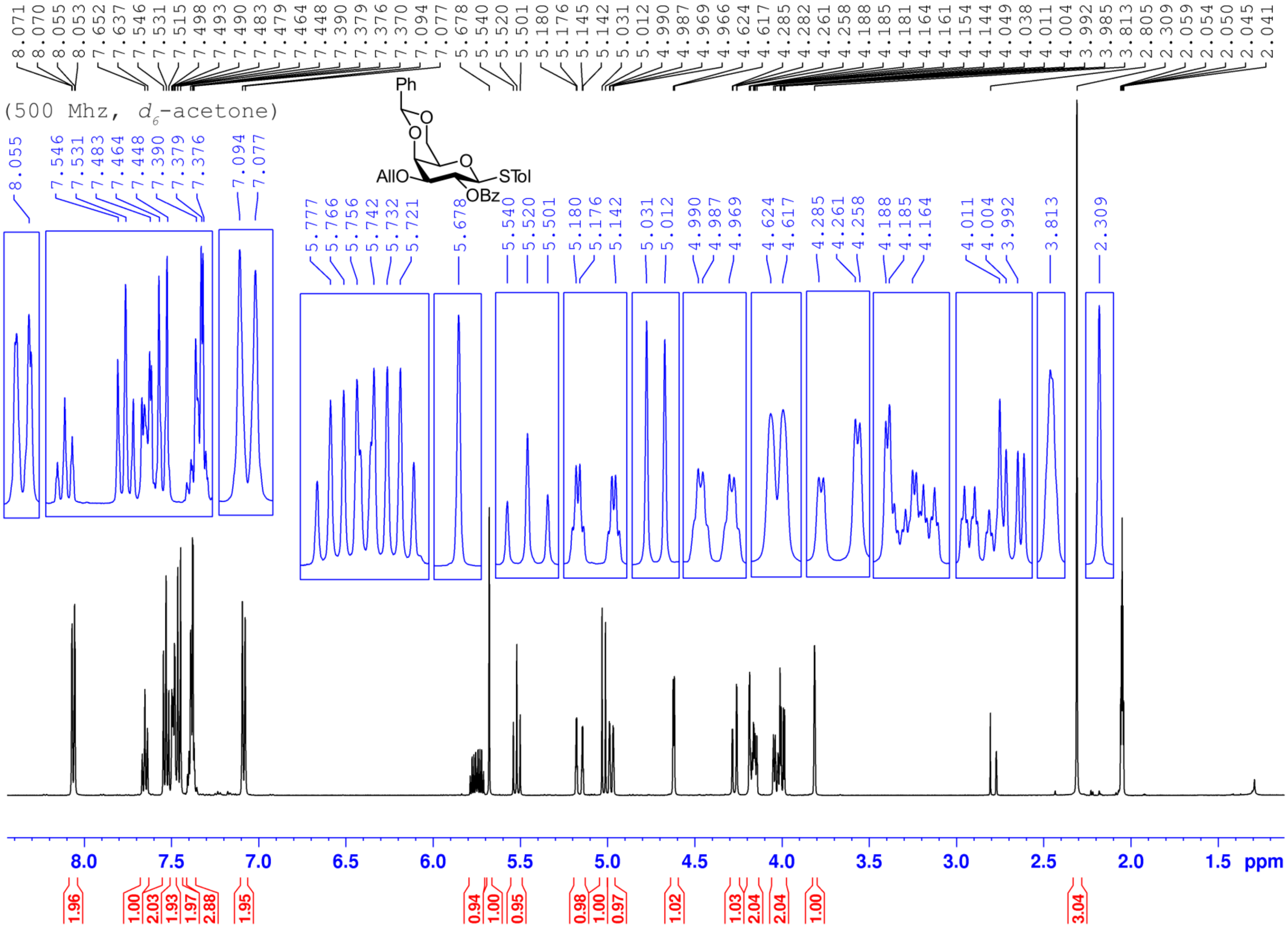

^{1}H-^{1}H COSY

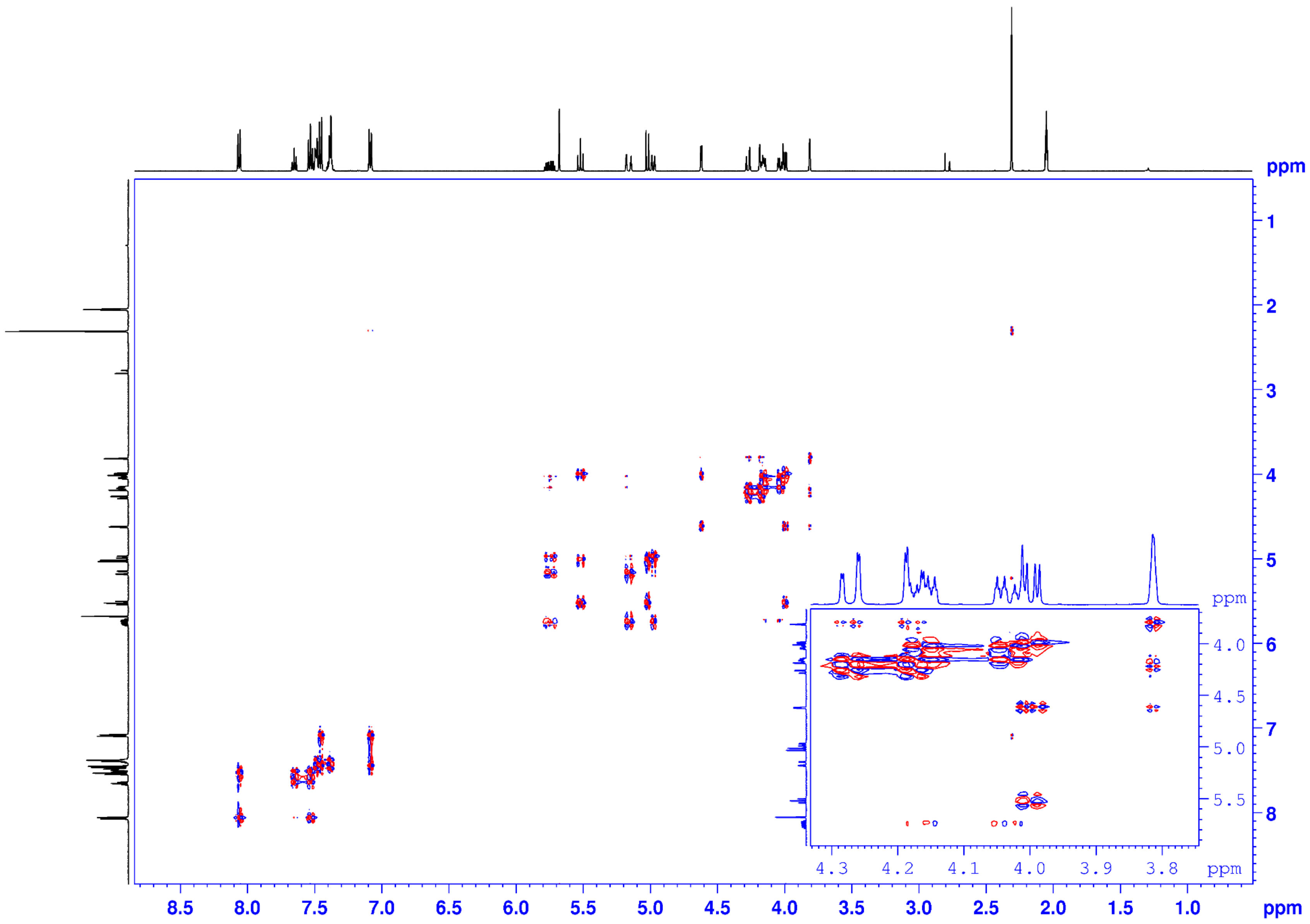

ppm
1
2
3
4
5
6
7
8
8.5
8.0
7.5
7.0
6.5
6.0
5.5
5.0
4.5
4.0
3.5
3.0
2.5
2.0
1.5
1.0
ppm
4.3
4.2
4.1
4.0
3.9
3.8
ppm
4.0
4.5
5.0
5.5
ppm

^1H-^{13}C HSQC

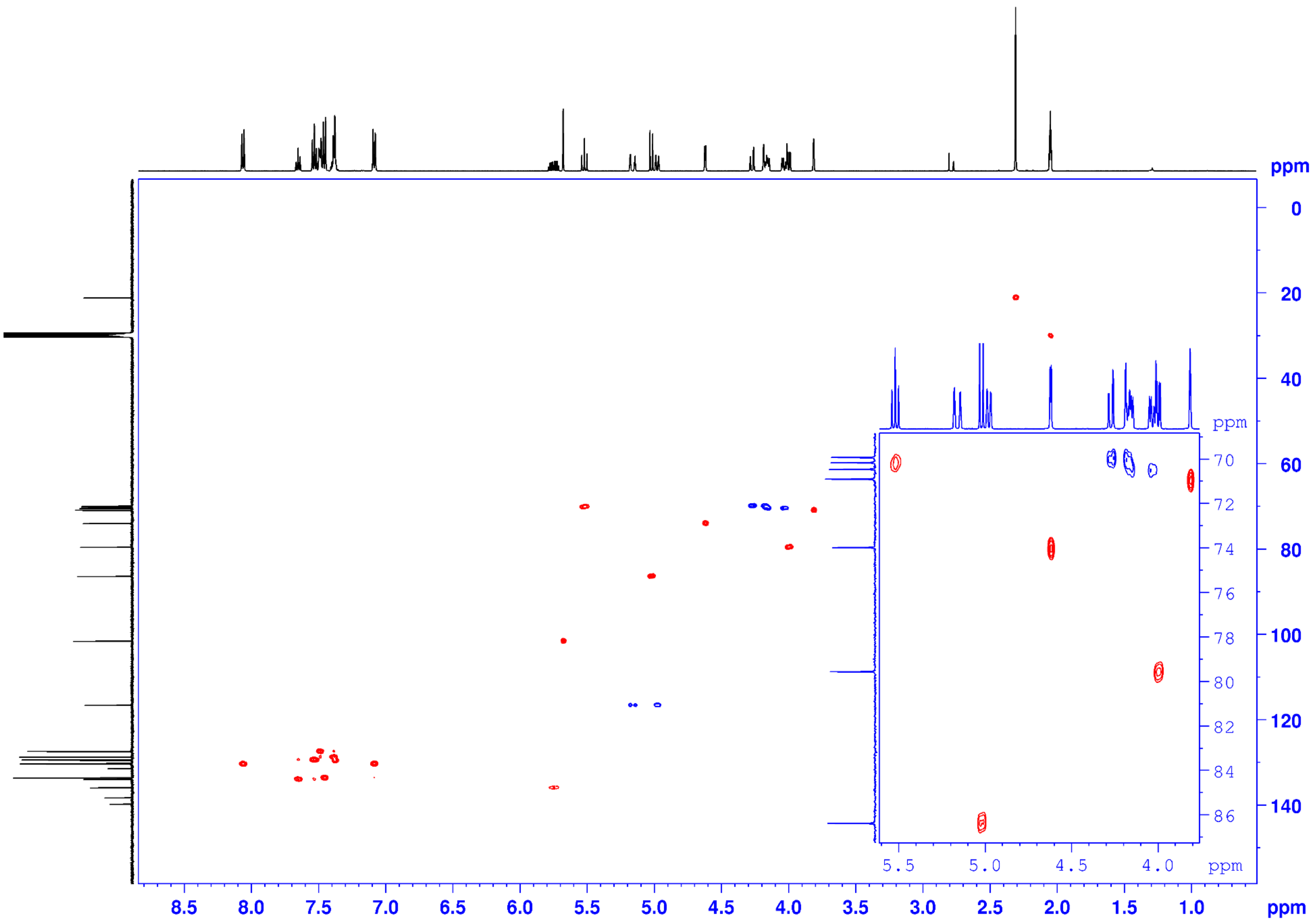

ppm
0
20
40
60
80
100
120
140
8.5
8.0
7.5
7.0
6.5
6.0
5.5
5.0
4.5
4.0
3.5
3.0
2.5
2.0
1.5
1.0
ppm
70
72
74
76
78
80
82
84
86
5.5
5.0
4.5
4.0
ppm

$^{13}C\{^{1}H\}$ NMR

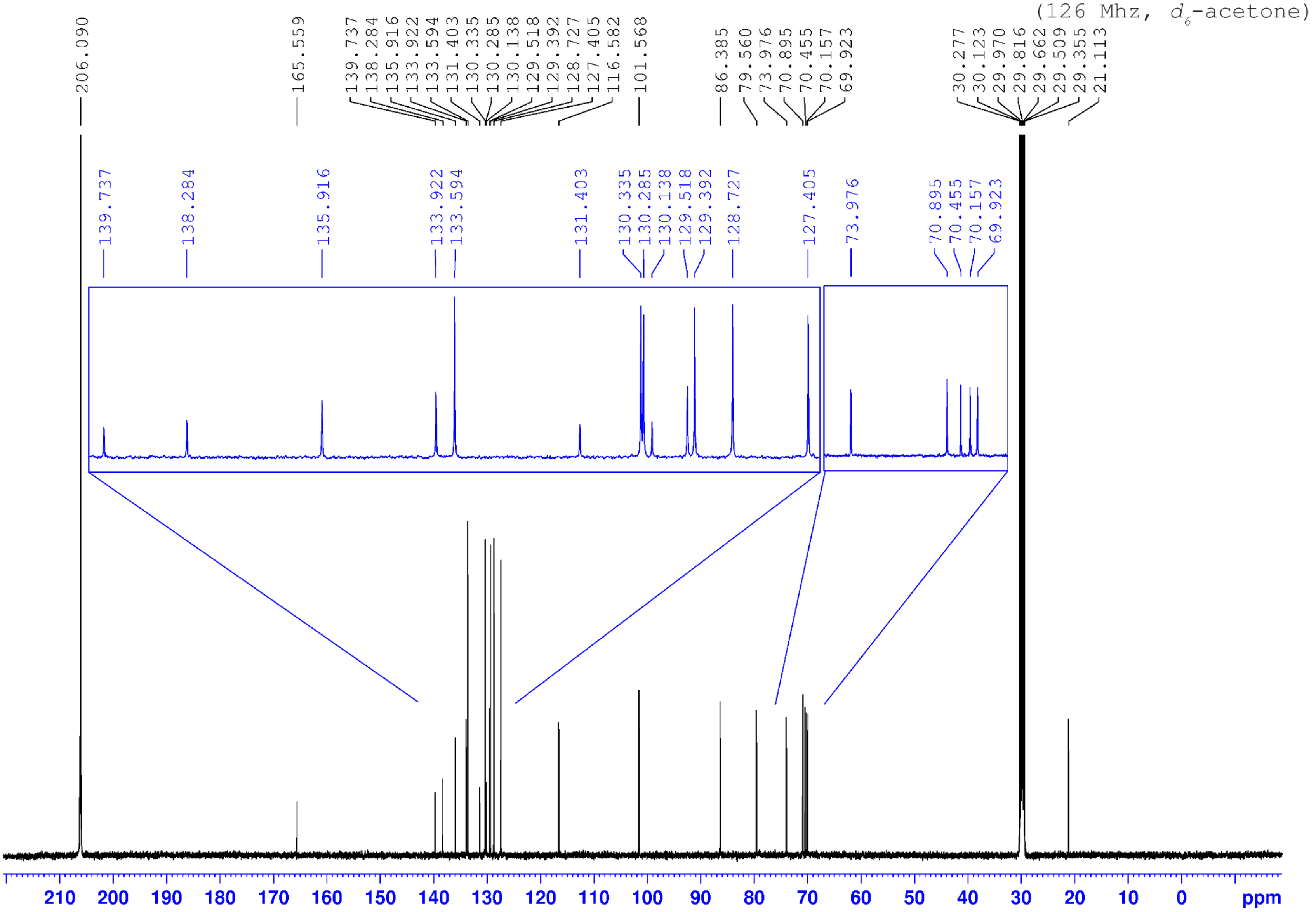
(126 Mhz, d6-acetone)
206.090
165.559
139.737
138.284
135.916
133.922
133.594
131.403
130.335
130.285
130.138
129.518
129.392
128.727
127.405
116.582
101.568
86.385
79.560
73.976
70.895
70.455
70.157
69.923
30.277
30.123
29.970
29.816
29.662
29.509
29.355
21.113
ppm

Compound **3e**

^{1}H-NMR

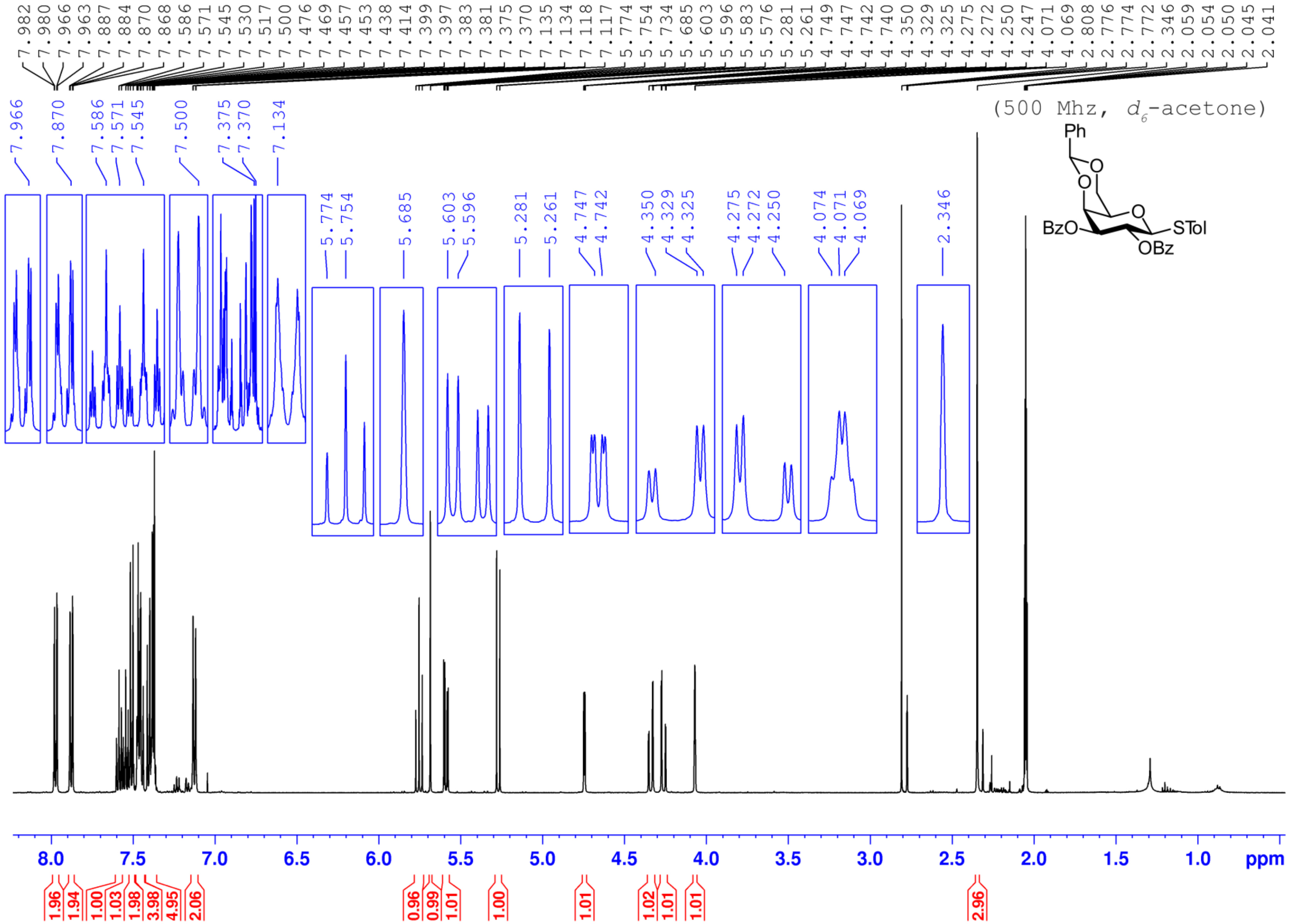

^{1}H-^{1}H COSY

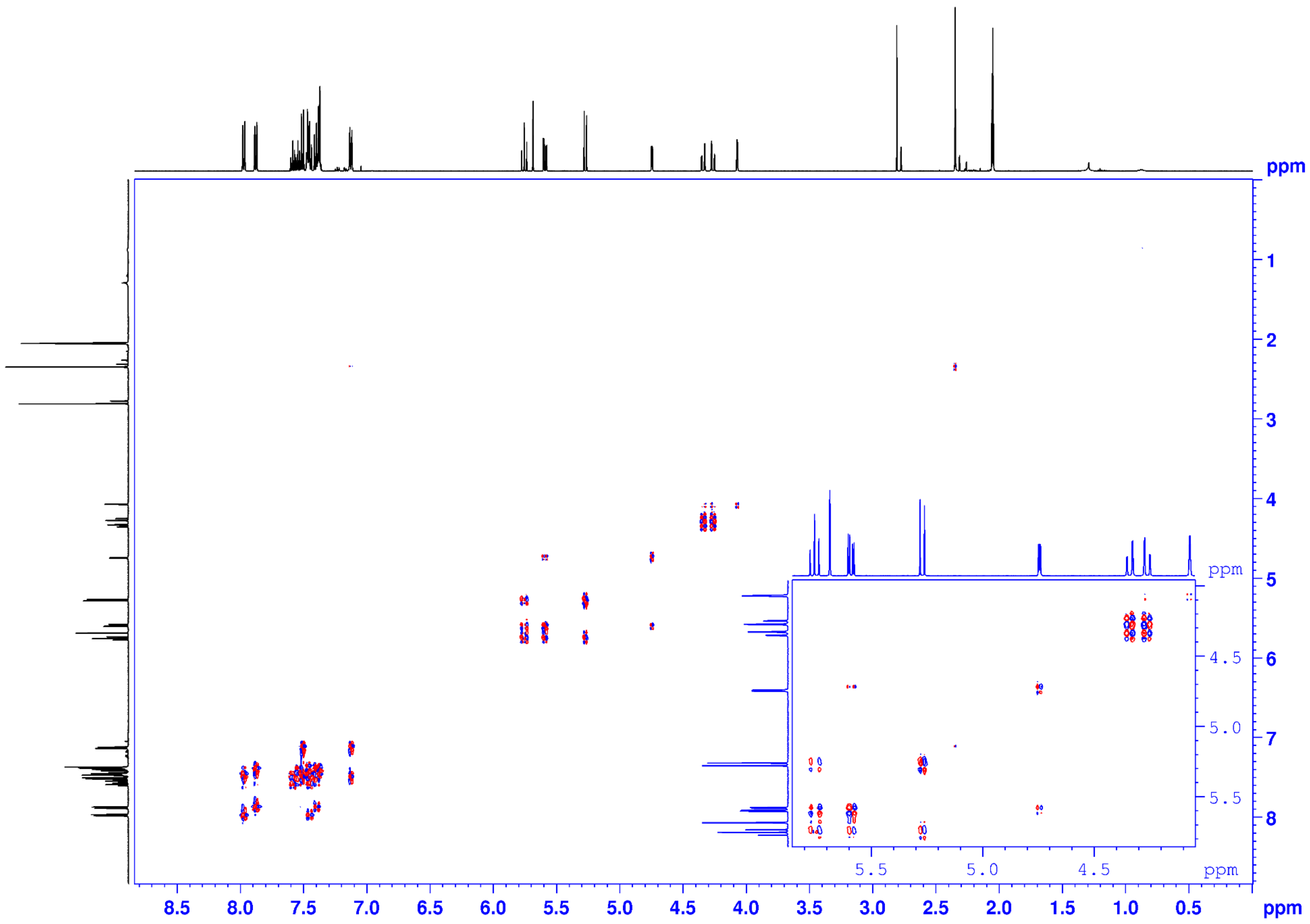
ppm
8.5
8.0
7.5
7.0
6.5
6.0
5.5
5.0
4.5
4.0
3.5
3.0
2.5
2.0
1.5
1.0
0.5
ppm
1
2
3
4
5
6
7
8
5.5
5.0
4.5
ppm
4.5
5.0
5.5
ppm

^{1}H-^{13}C HSQC

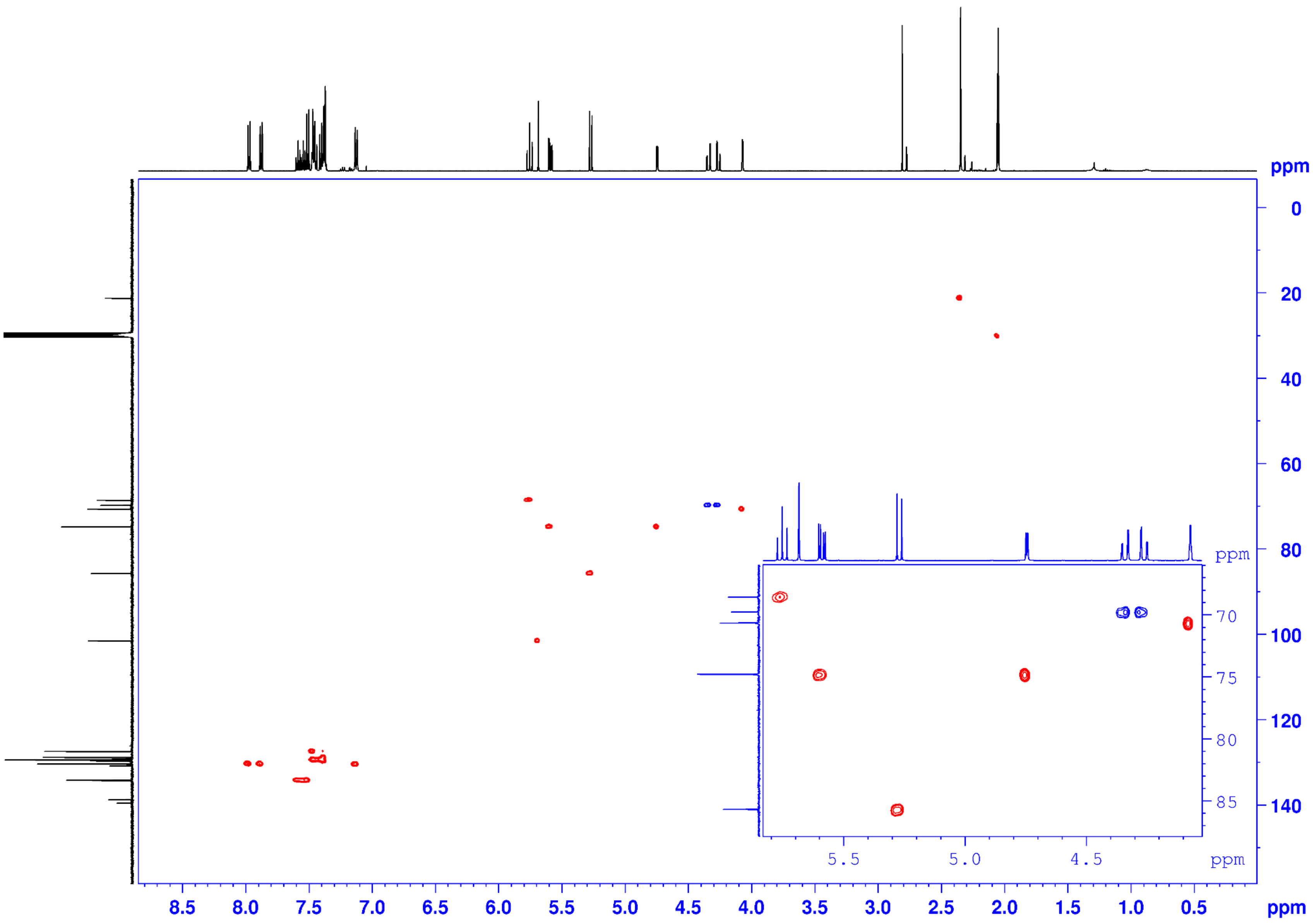

ppm
0
20
40
60
80
100
120
140
8.5
8.0
7.5
7.0
6.5
6.0
5.5
5.0
4.5
4.0
3.5
3.0
2.5
2.0
1.5
1.0
0.5
ppm
70
75
80
85
5.5
5.0
4.5

$^{13}C\{^1H\}$ NMR

(126 Mhz, d_6-acetone)

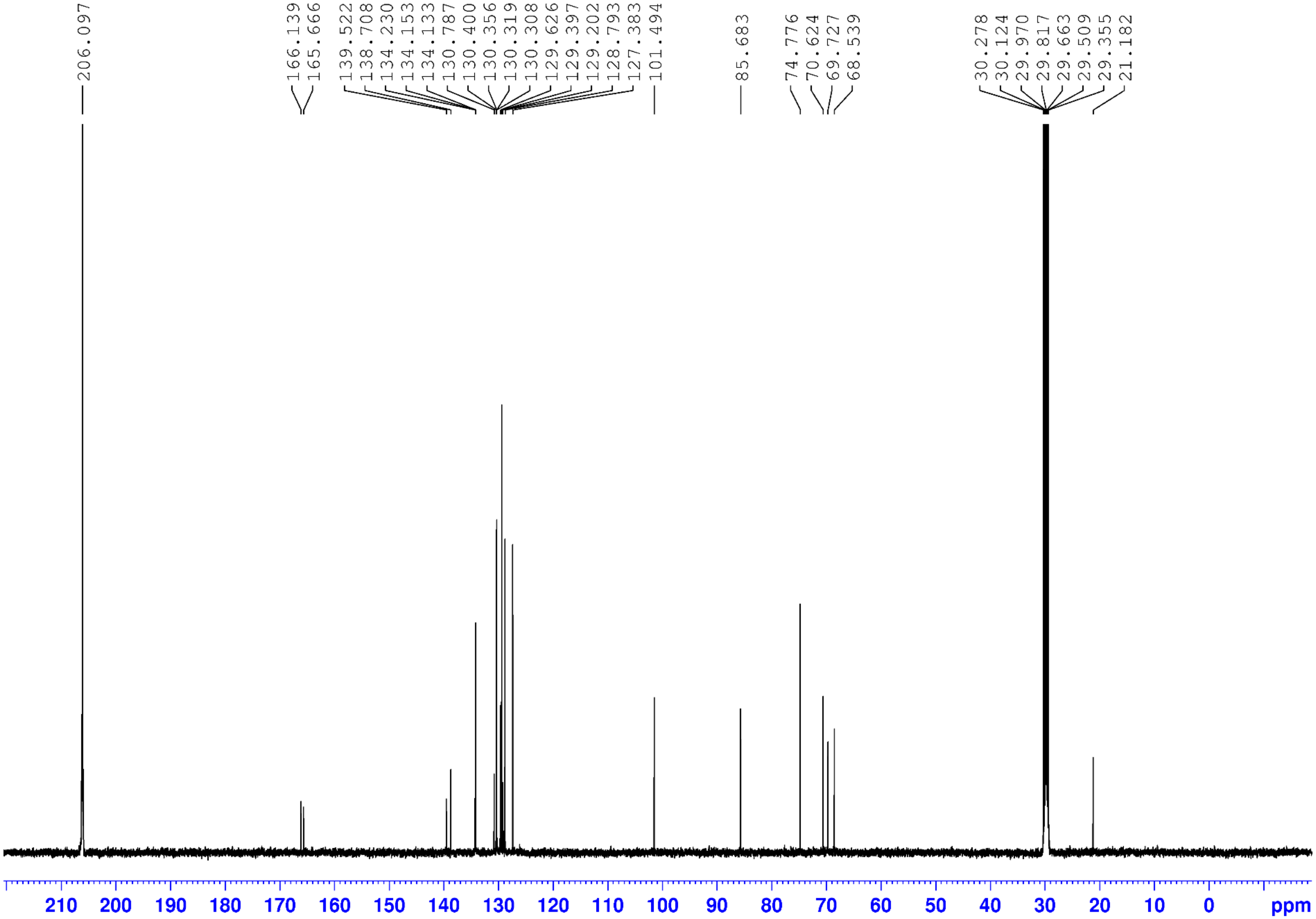

Compound **4**

^{1}H-NMR

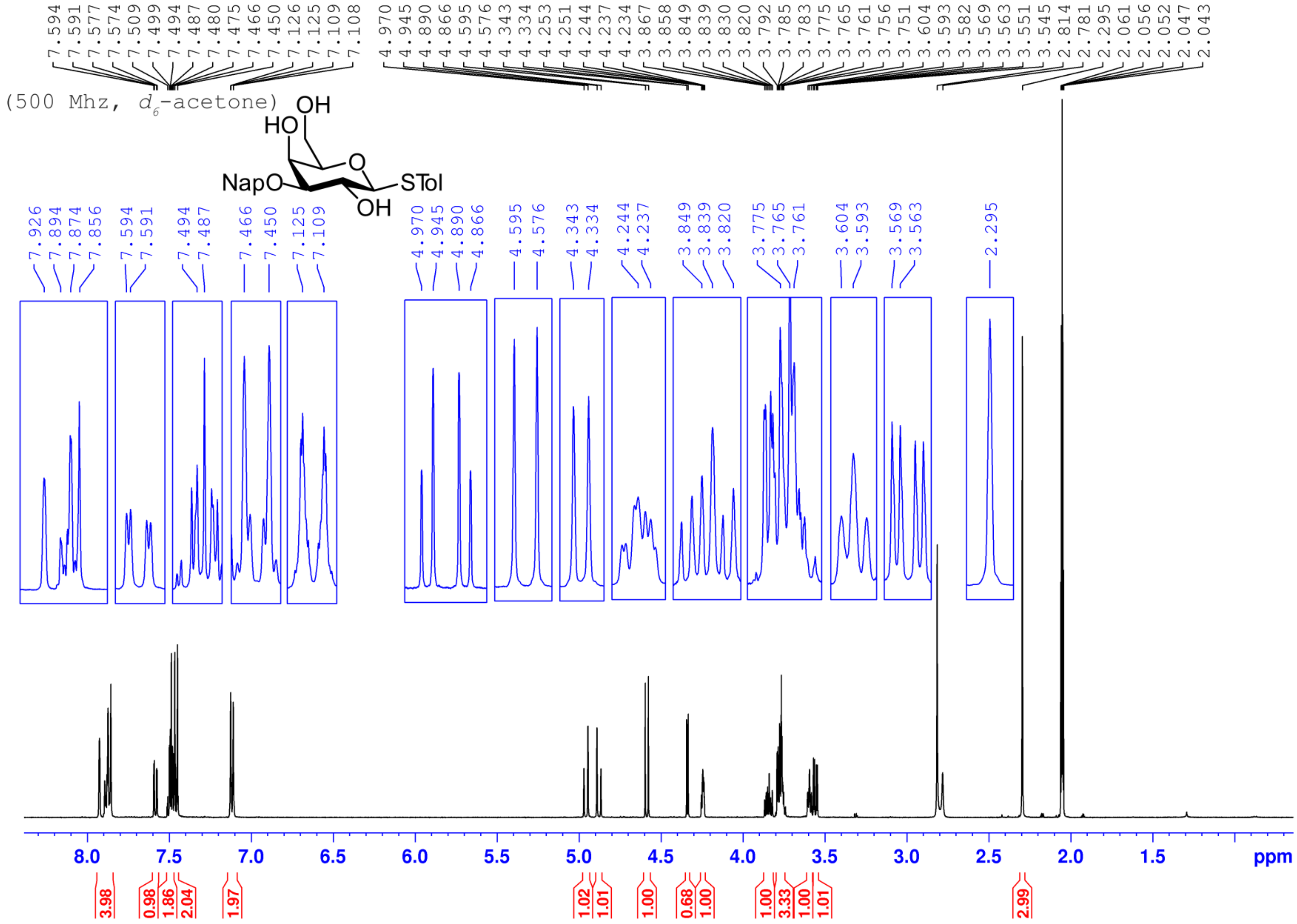

^{1}H-^{1}H COSY

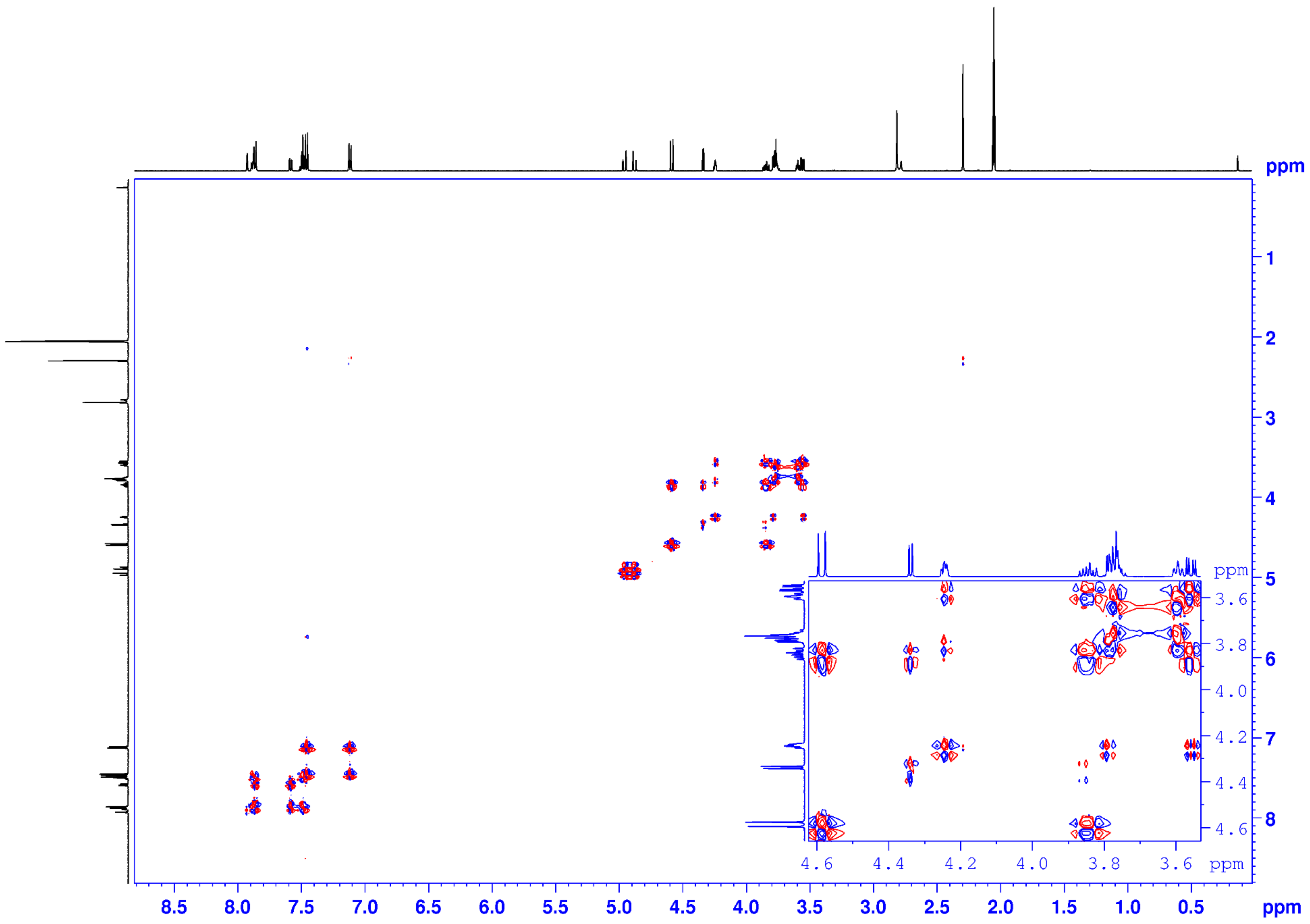

ppm
1
2
3
4
5
6
7
8
8.5
8.0
7.5
7.0
6.5
6.0
5.5
5.0
4.5
4.0
3.5
3.0
2.5
2.0
1.5
1.0
0.5
ppm
3.6
3.8
4.0
4.2
4.4
4.6
4.6
4.4
4.2
4.0
3.8
3.6
ppm

^{1}H-^{13}C HSQC

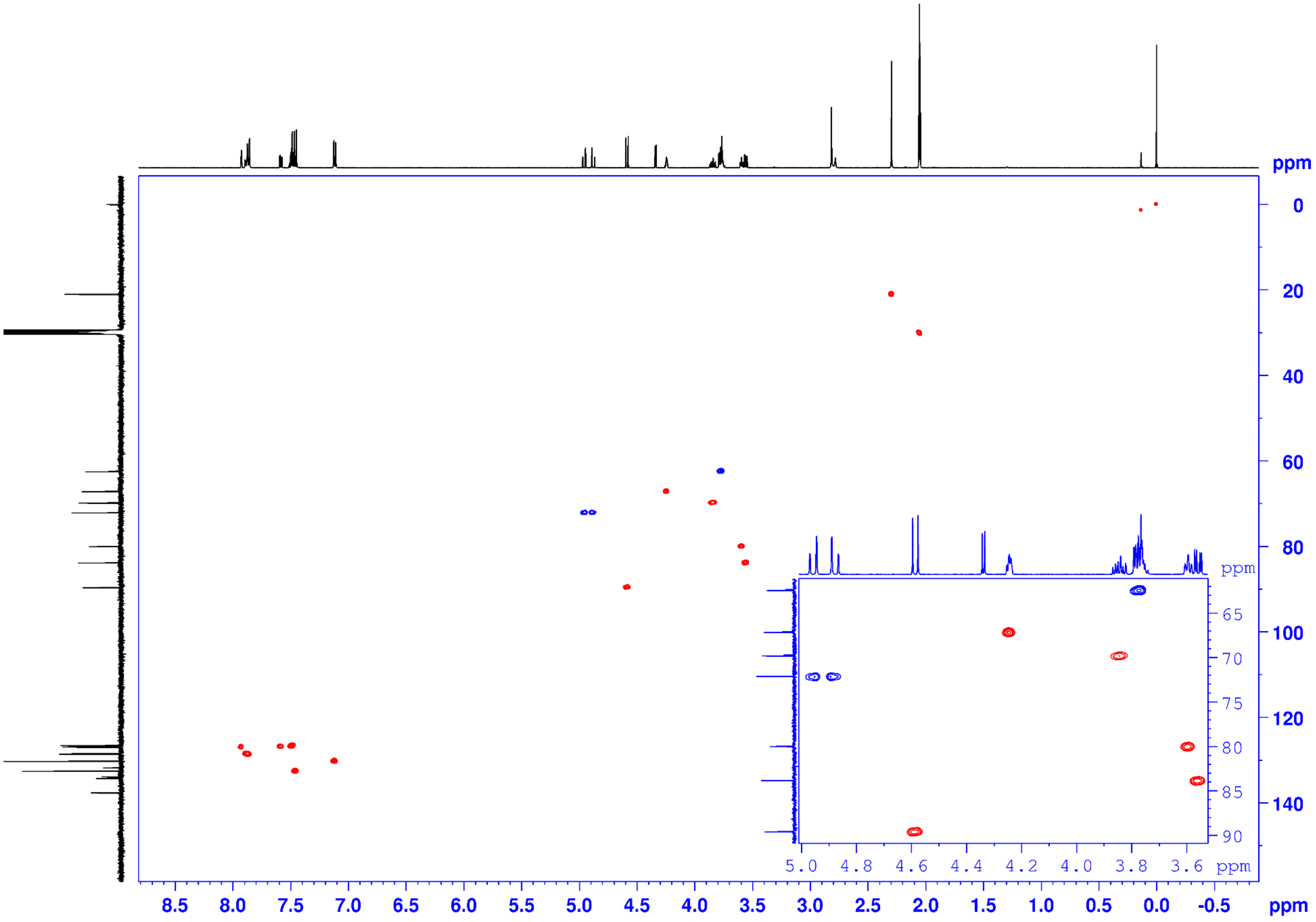

ppm
0
20
40
60
80
100
120
140
8.5
8.0
7.5
7.0
6.5
6.0
5.5
5.0
4.5
4.0
3.5
3.0
2.5
2.0
1.5
1.0
0.5
0.0
-0.5
ppm
65
70
75
80
85
90
5.0
4.8
4.6
4.4
4.2
4.0
3.8
3.6
ppm

$^{13}C\{^1H\}$ NMR
(126 Mhz, d_6-acetone)

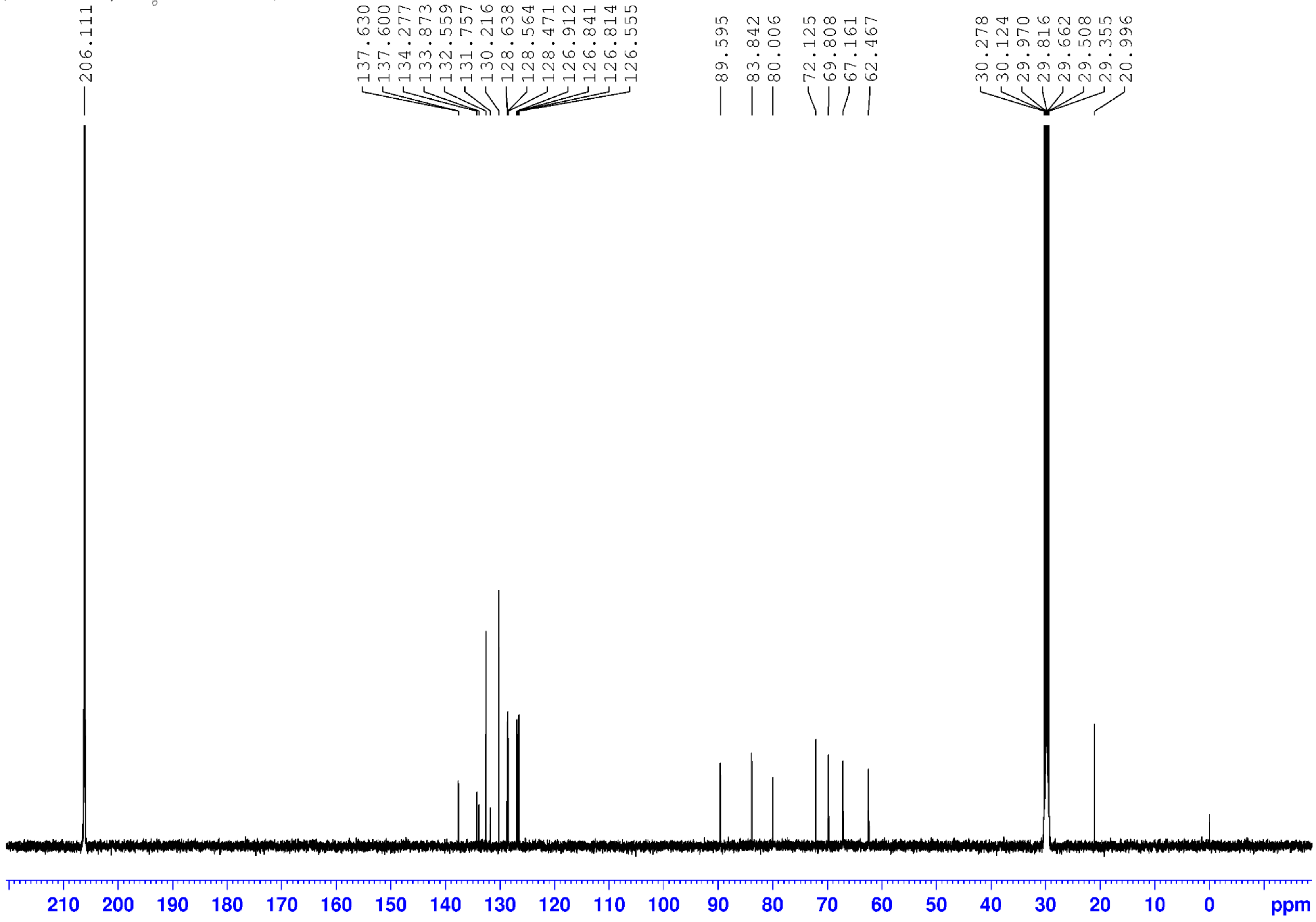

Compound **5**

^{1}H-NMR

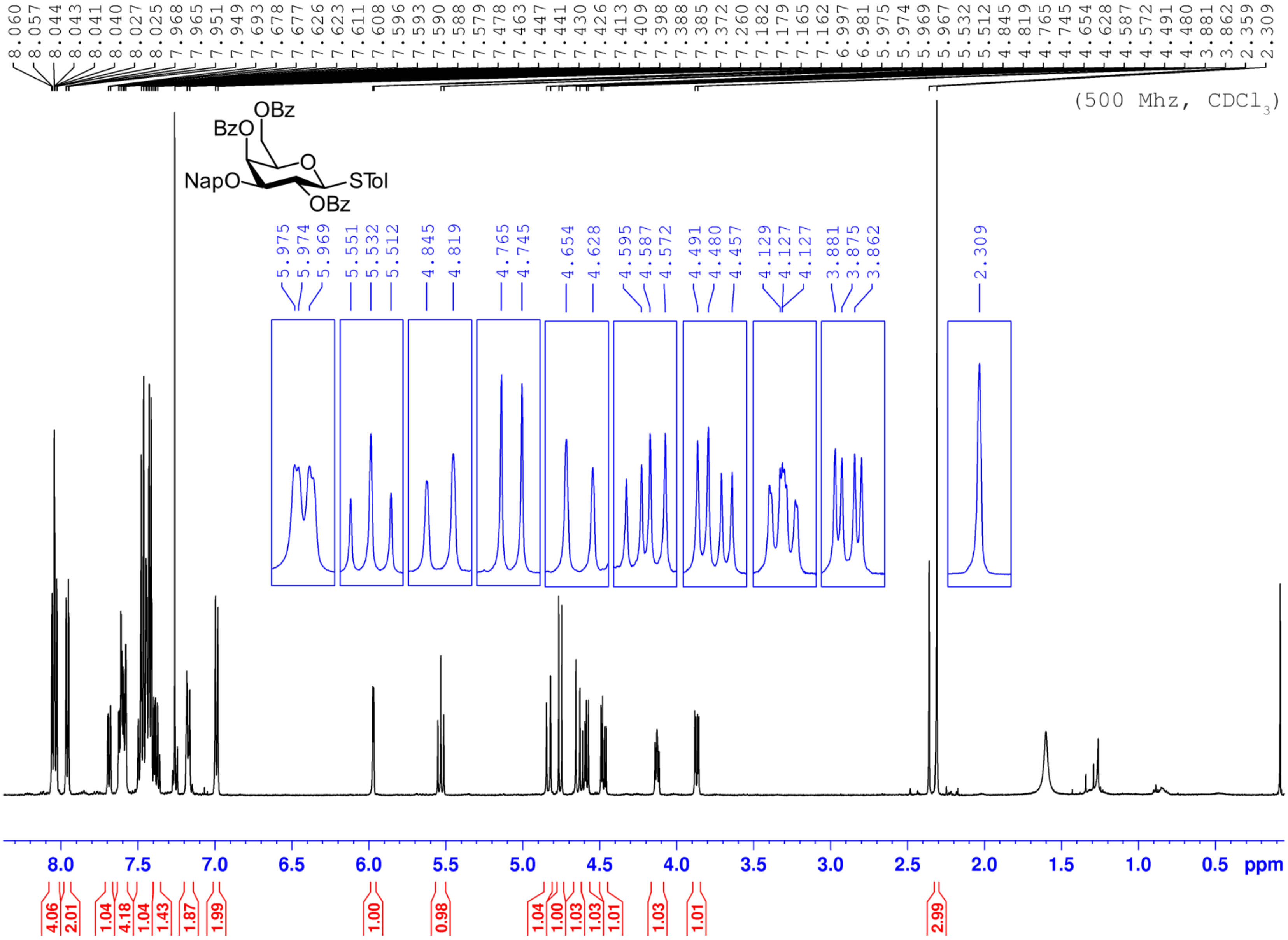

^{1}H-^{1}H COSY

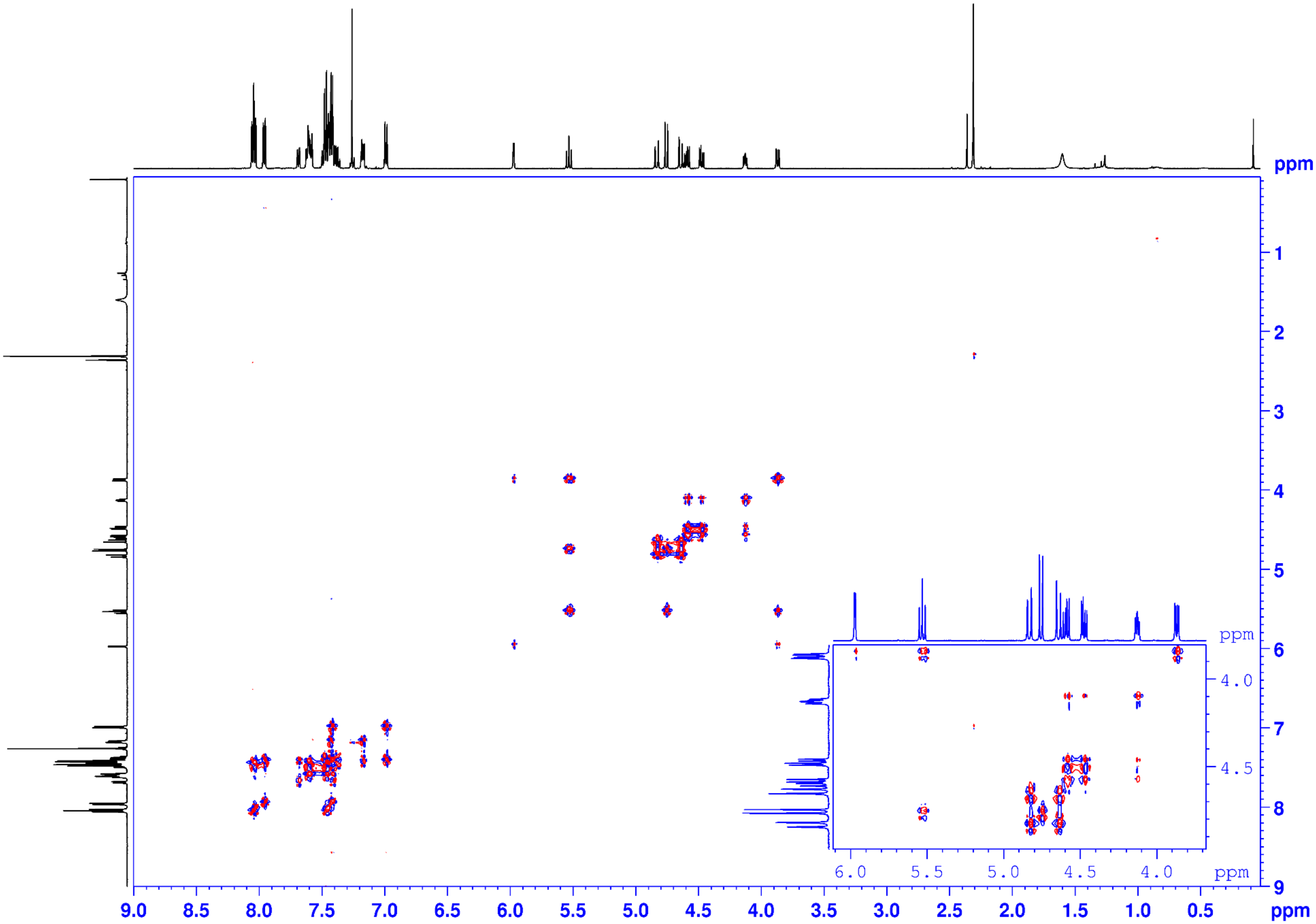
ppm
1
2
3
4
5
6
7
8
9
9.0
8.5
8.0
7.5
7.0
6.5
6.0
5.5
5.0
4.5
4.0
3.5
3.0
2.5
2.0
1.5
1.0
0.5
ppm
4.0
4.5
6.0
5.5
5.0
4.5
4.0
ppm

^{1}H-^{13}C HSQC

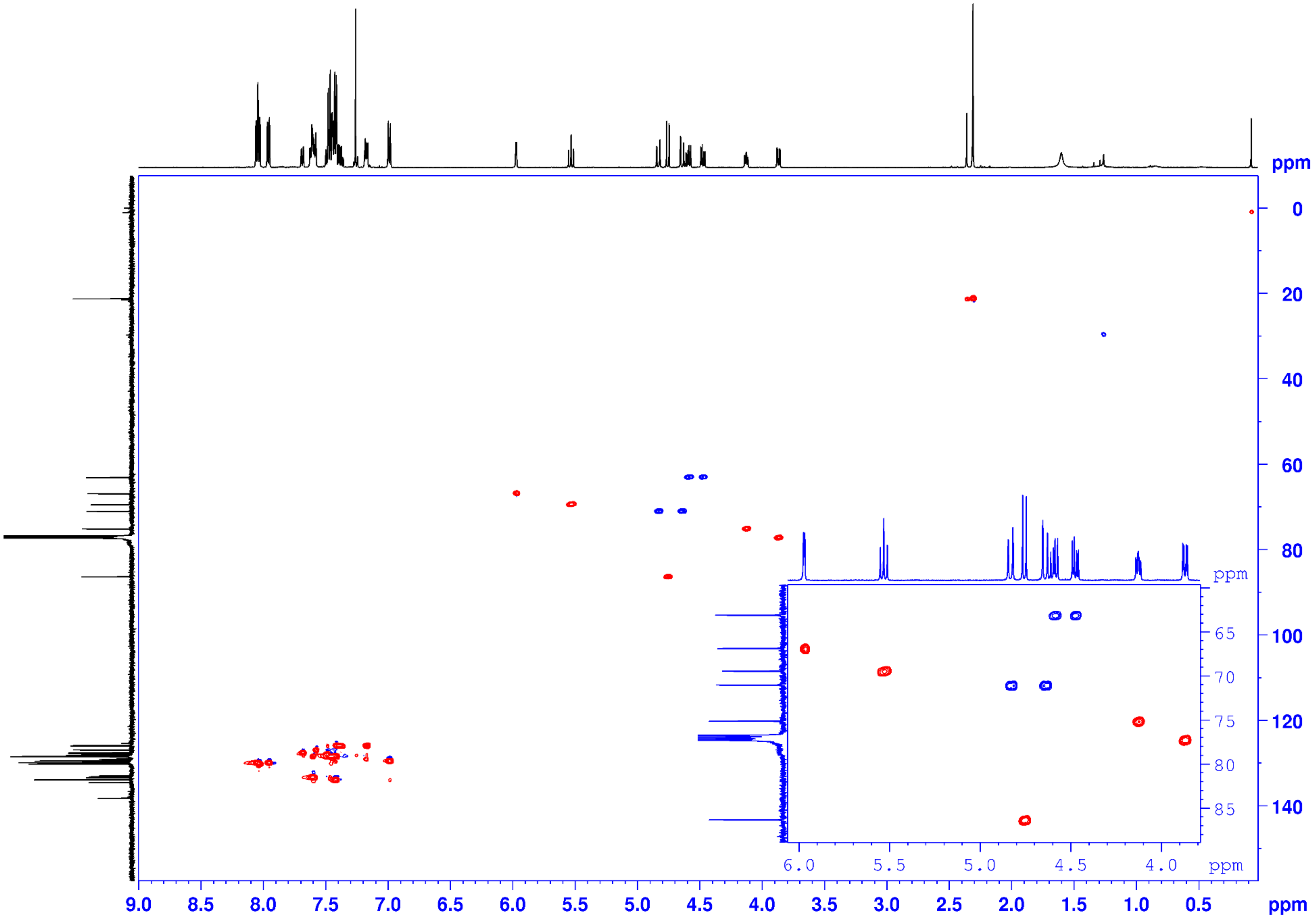

ppm
0
20
40
60
80
100
120
140
9.0
8.5
8.0
7.5
7.0
6.5
6.0
5.5
5.0
4.5
4.0
3.5
3.0
2.5
2.0
1.5
1.0
0.5
ppm
65
70
75
80
85
6.0
5.5
5.0
4.5
4.0
ppm

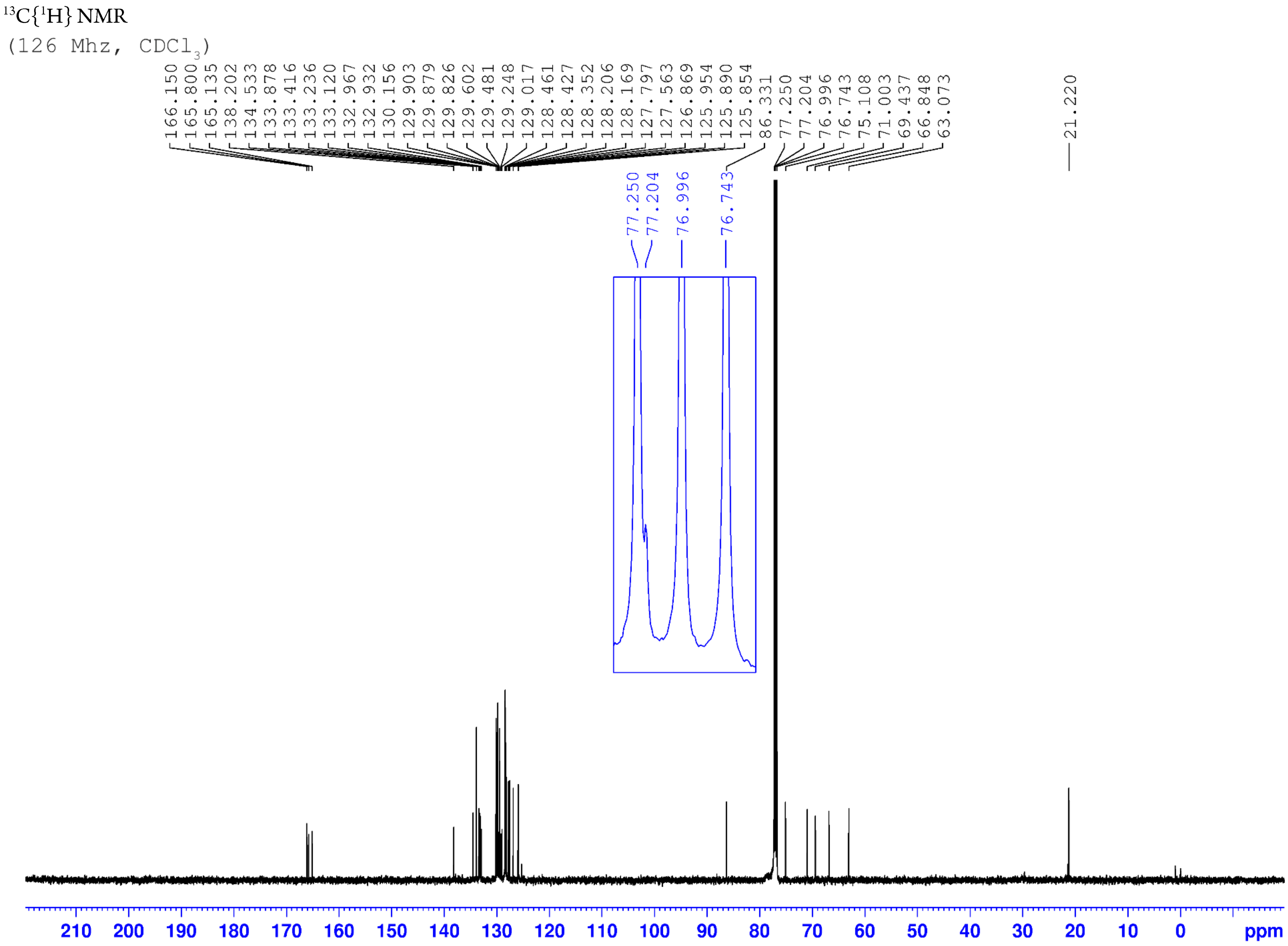

$^{13}C\{^{1}H\}$ NMR
(126 Mhz, $CDCl_3$)
166.150
165.800
165.135
138.202
134.533
133.878
133.416
133.236
133.120
132.967
132.932
130.156
129.903
129.879
129.826
129.602
129.481
129.248
129.017
128.461
128.427
128.352
128.206
128.169
127.797
127.563
126.869
125.954
125.890
125.854
86.331
77.250
77.204
76.996
76.743
75.108
71.003
69.437
66.848
63.073
21.220
210 200 190 180 170 160 150 140 130 120 110 100 90 80 70 60 50 40 30 20 10 0 ppm

Compound **6**

^{1}H-NMR

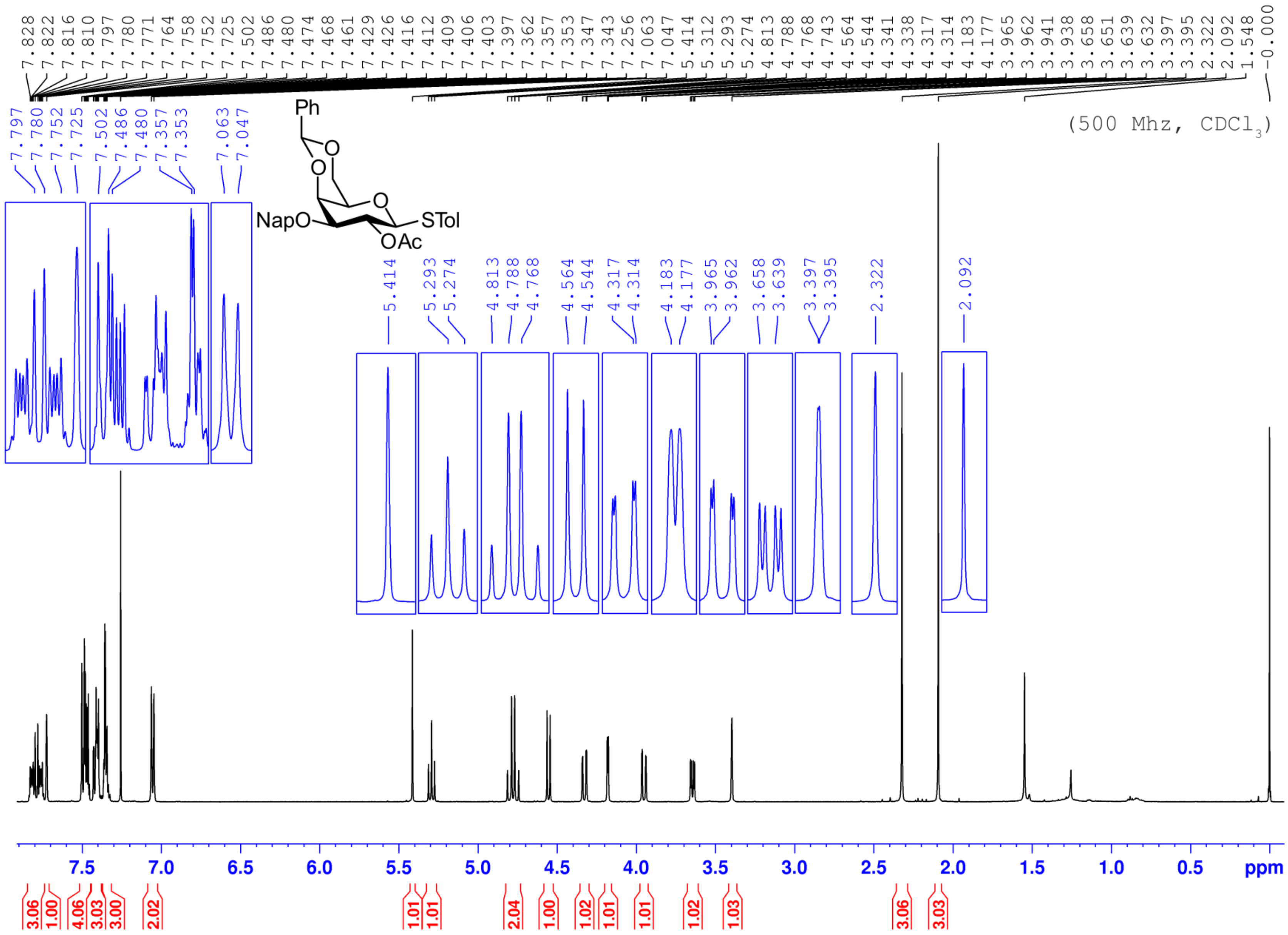

^1H-^1H COSY

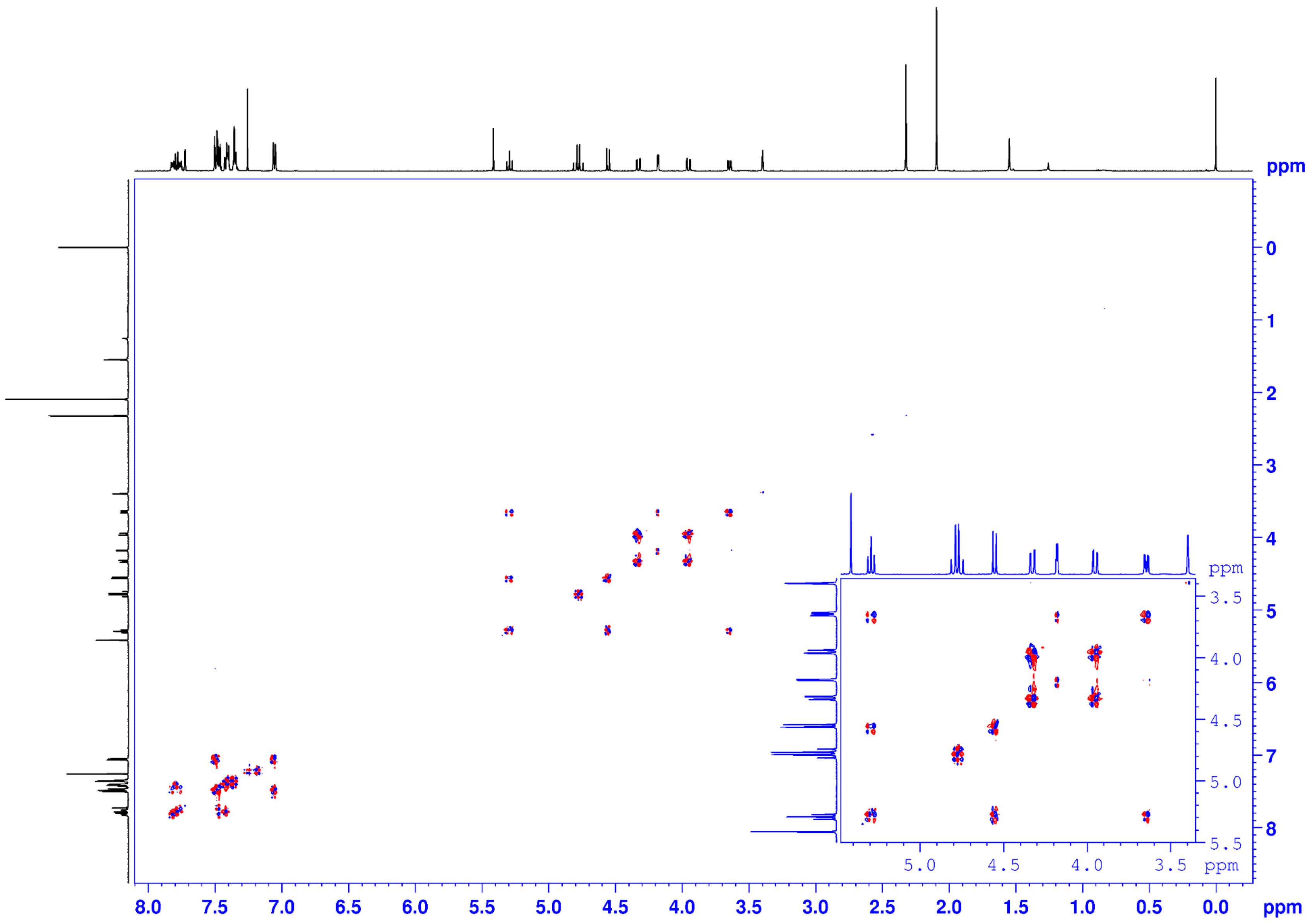
ppm
0
1
2
3
4
5
6
7
8
8.0
7.5
7.0
6.5
6.0
5.5
5.0
4.5
4.0
3.5
3.0
2.5
2.0
1.5
1.0
0.5
0.0
ppm
3.5
4.0
4.5
5.0
5.5
ppm
5.0
4.5
4.0
3.5
ppm

^{1}H-^{13}C HSQC

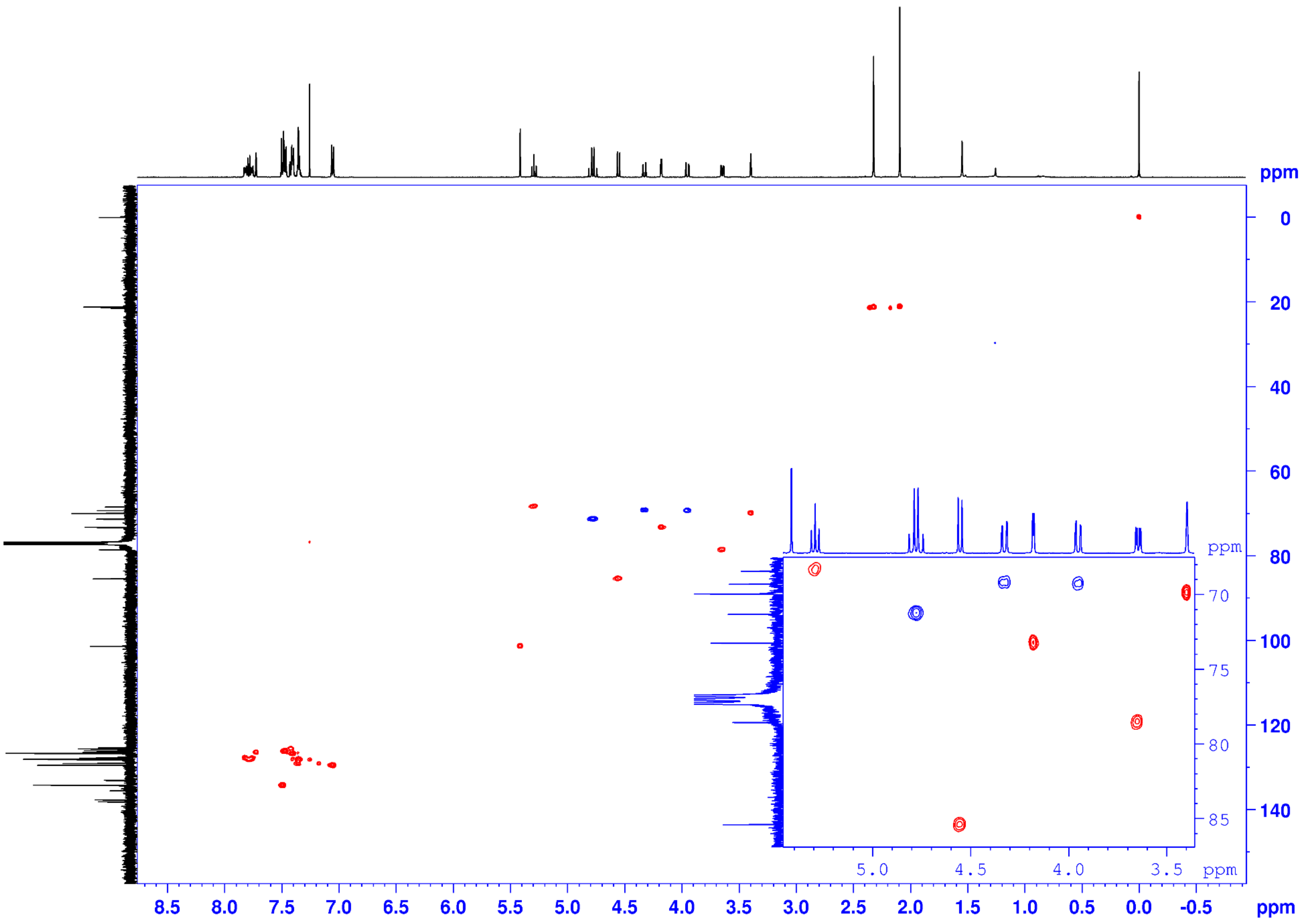
ppm
0
20
40
60
80
100
120
140
8.5
8.0
7.5
7.0
6.5
6.0
5.5
5.0
4.5
4.0
3.5
3.0
2.5
2.0
1.5
1.0
0.5
0.0
-0.5
ppm
70
75
80
85
5.0
4.5
4.0
3.5
ppm

$^{13}C\{^{1}H\}$ NMR

(126 Mhz, $CDCl_3$)

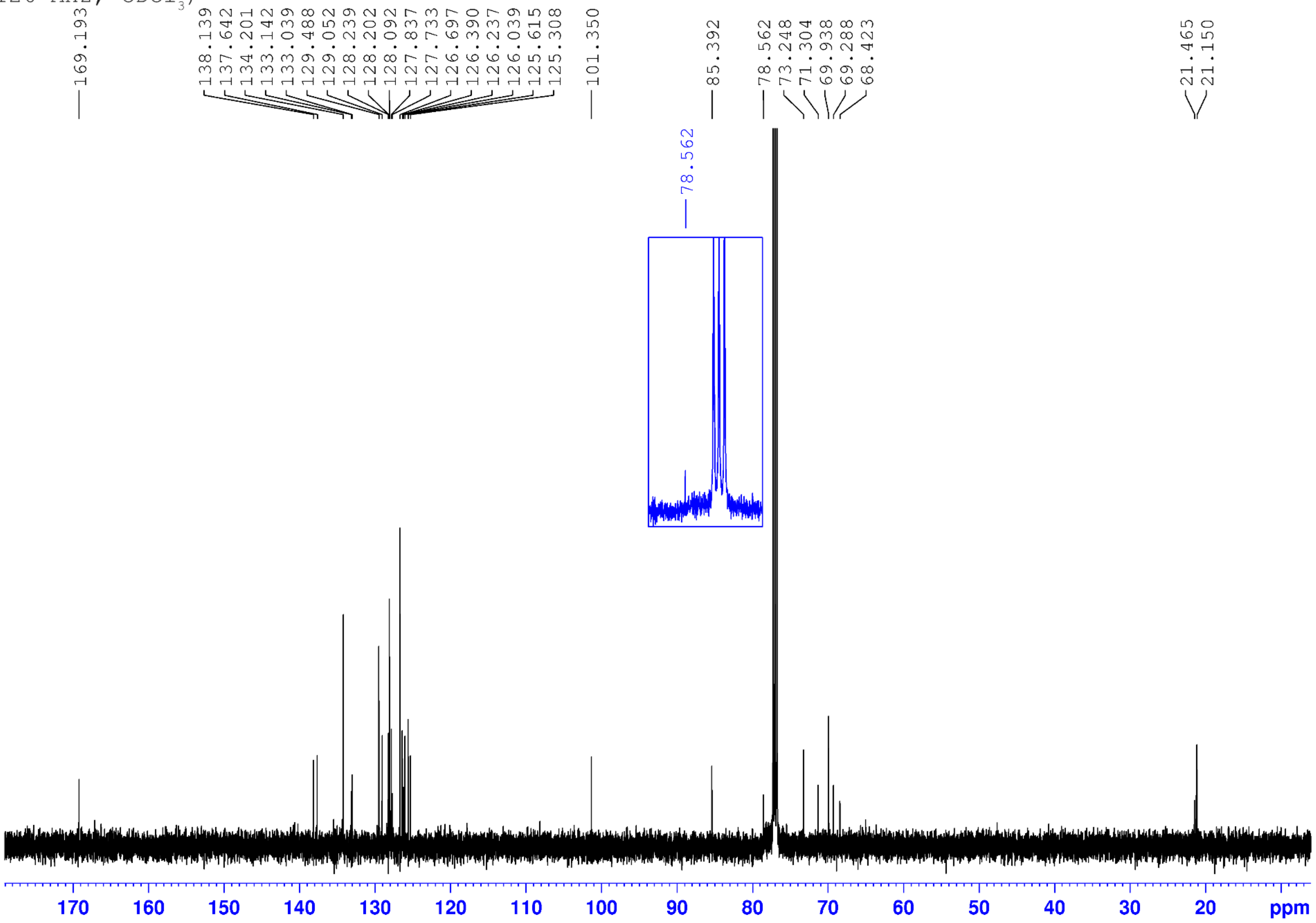

References

1. Chen, C. T., Weng, S. S., Kao, J. Q., Lin, C. C. & Jan, M. D. Stripping off water at ambient temperature: Direct atom-efficient acetal formation between aldehydes and diols catalyzed by water-tolerant and recoverable vanadyl triflate. *Org. Lett.* **7**, 3343–3346 (2005).

2. Verma, N. *et al.* Threshold of Thioglycoside Reactivity Difference Is Critical for Efficient Synthesis of Type i Oligosaccharides by Chemoselective Glycosylation. *J. Org. Chem.* **86**, 892–916 (2021).

3. Ding, N., Li, C., Liu, Y., Zhang, Z. & Li, Y. Concise synthesis of clarhamnoside, a novel glycosphingolipid isolated from the marine sponge Agela clathrodes. *Carbohydr. Res.* **342**, 2003–2013 (2007).

4. Lv, J., Liu, C. Y., Guo, Y. F., Feng, G. J. & Dong, H. SnCl2-Catalyzed Acetalation/Selective Benzoylation Sequence for the Synthesis of Orthogonally Protected Glycosyl Acceptors. *European J. Org. Chem.* **2022**, e202101565 (2022).

5. Dolomanov, O. V., Bourhis, L. J., Gildea, R. J., Howard, J. A. K. & Puschmann, H. OLEX2: a complete structure solution, refinement and analysis program. *urn:issn:0021-8898* **42**, 339–341 (2009).

6. Sheldrick, G. M. & IUCr. SHELXT – Integrated space-group and crystal-structure determination. *urn:issn:2053-2733* **71**, 3–8 (2015).

7. Sheldrick, G. M. & IUCr. Crystal structure refinement with SHELXL. *urn:issn:2053-2296* **71**, 3–8 (2015).

8. Zhang, Z. *et al.* Programmable one-pot oligosaccharide synthesis. *J. Am. Chem. Soc.* **121**, 734–753 (1999).

9. Frisch, M. J. *et al.* Gaussian 16 Revision C.01. *Gaussian, Inc., Wallingford CT* (2016).
